# Regional Climate Drives Spatial Variation in Species Richness in the Most Diverse Family of Neotropical Snakes (Colubroidea: Dipsadidae)

**DOI:** 10.1002/ece3.71716

**Published:** 2025-07-07

**Authors:** Juan P. Ramírez, Julián A. Velasco, Tod W. Reeder

**Affiliations:** ^1^ Department of Biology San Diego State University San Diego California USA; ^2^ Instituto de Ciencias de la Atmósfera y Cambio Climático Universidad Nacional Autónoma de México Ciudad de México México; ^3^ Department of Evolution, Ecology, and Organismal Biology University of California, Riverside Riverside California USA

**Keywords:** climate, dispersal, diversification, evolutionary time, macroecology, Neotropics, species diversity

## Abstract

Species richness gradients are frequently associated with spatial variation of environmental conditions. However, understanding how the regional environment influences species assemblages is an ongoing topic of discussion, with three non‐exclusive explanations being proposed. Under these hypotheses, climate can determine the richness of regions in two main ways: (1) directly, by determining their carrying capacity, or (2) indirectly, through its effects on either (a) lineage diversification (i.e., the rates of speciation and/or extinction), or (b) the duration over which regions have been accumulating species. Recently, some studies have started to simultaneously evaluate and compare the role of these mechanisms in determining richness gradients. For this work, we set out to identify the factors that determine the spatial variation in richness of the hyperdiverse snake family Dipsadidae. To achieve this goal, we first calculated the number of species of the group in 100 × 100 km cells throughout its distribution. Then, using piecewise structural equations, we evaluated and compared the ability to predict the richness of these cells by several environmental (current climatic seasonality and past climatic stability) and evolutionary variables (speciation rates and time for speciation). We found that the richness gradient of Dipsadidae is directly explained by current seasonality and productivity, with a limited role of macroevolutionary processes. Therefore, our results support the idea that current climate has a primary role in determining the geographic patterns of the richness of the group, with the influence of diversification and time on regional diversity potentially obscured by the elevated levels of dispersal within the group. Such a possibility needs to be evaluated in future studies that explicitly assess the impact of dispersal on regional richness, both for the Dipsadidae and other groups where similar results have been reported.

## Graphical abstract

Our study investigated the relative importance of the macroecological and macroevolutionary factors driving the patterns of species richness of the hyperdiverse snake family Dipsadidae across the Americas. By utilizing piecewise structural equations, we discovered that current climatic seasonality and productivity are the main drivers of species richness, with macroevolutionary mechanisms such as regional speciation and evolutionary time playing a limited role. However, the influence of diversification and time on regional diversity may be obscured by the high dispersal levels within the group — a possibility that needs to be explored in future studies, although our ability to do so is constrained by the limitations of current methodological approaches.

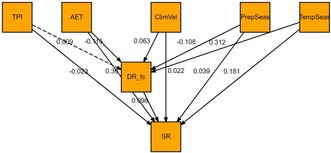

## 1 Introduction

Understanding how the observed variation of species richness through space originated and is maintained through time has been the focus of much interest for the last two centuries (von Humboldt 1808; Hawkins 2001). As a result of this interest, plenty of hypotheses to explain the origin of species richness gradients have been proposed (at least 120; Palmer 1994), each invoking the role of a different ecological and/or evolutionary process (Palmer 1994). We now recognize that many of these hypotheses are not independent of each other, or they cannot explain species richness by themselves (Rohde 1992; Palmer 1994). Despite this, there is still an ongoing debate about which processes have a more important role in determining the existence of species richness gradients (Wiens 2011; Marin et al. 2018; Brodie and Mannion 2023; Saupe 2023).

Traditionally, spatial variation in species richness has been considered as resulting from the influence of climatic variables (e.g., temperature, precipitation, productivity, and their short‐ and long‐term variation) on the geographic range limits of individual species, on the niche space or the carrying capacity of regions, or both (Roy and Goldberg 2007; Stevens 2011; Storch and Okie 2019). In support of this idea, many studies have found strong, significant relationships between values of environmental variables and species richness (Ricklefs 2006; Wiens 2007; Tello and Stevens 2010). However, the direction and strength of climate‐richness relationships exhibit considerable variation across regions and taxa (Roy and Goldberg 2007). That this is the case suggests that even geographical patterns shared among many lineages (e.g., the latitudinal and altitudinal diversity gradients) likely do not result from a single mechanism (Tello and Stevens 2010; Cerezer et al. 2022).

Further, finding associations between species richness gradients and the spatial variation in one or more climatic variables does not necessarily indicate that such a relationship is causal (Kozak and Wiens 2012). Instead, it is now recognized that associations between species richness and climatic variables are mediated by the only processes that can directly change the number of species existing in each region: speciation, extinction, and dispersal (Wiens and Donoghue 2004; Wiens 2011; Saupe 2023). As a result, understanding the mechanisms responsible for large‐scale species richness patterns requires integrating these three evolutionary processes with the environmental variables correlating with species richness (Wiens 2007; Saupe 2023). Despite this, it is not uncommon for studies to consider that diversity gradients result from climate directly determining the carrying capacity of regions, with some of them finding evidence supporting this possibility (Machac 2020; García‐Andrade et al. 2021). However, other studies have not found this to be the case (Qian et al. 2015; Gamisch and Comes 2019; see also Cerezer et al. 2022). In general, apart from the possibility of the direct influence of regional climate, there are two other non‐exclusive explanations for the existence of patterns of spatial variation in species richness: the *diversification rates* and *time* hypotheses (Stephens and Wiens 2003; Wiens 2011). Under these hypotheses, regional climatic conditions either do not influence species richness gradients or do so indirectly. Indeed, under the diversification rate hypothesis, regions harboring more species are expected to have higher net diversification (=speciation‐extinction) rates among their members, with these rates themselves influenced by factors such as region area or climate (Kozak and Wiens 2012; Qian et al. 2015). Thus, regions with conditions that promote lineage diversification are expected to exhibit increased species richness values.

On the other hand, under the time hypothesis, the increased species richness observed in some regions results from them having accumulated species for longer timespans, potentially resulting from characteristics they exhibit, such as their area or climate (Kozak and Wiens 2012; Qian et al. 2015; Cai et al. 2020). Thus, regions containing more species are expected to have been colonized earlier or to harbor older taxa than elsewhere, as they had more time for speciation events to occur (Stephens and Wiens 2003; Qian et al. 2015; Kozak 2017; Cai et al. 2020). However, limited dispersal between different regions can also cause variation in the evolutionary times (=time for speciation) they exhibit and, as a result, in their species richness (Mittelbach et al. 2007; Kozak 2017). That could be the case if taxa have a strong tendency to conserve their ancestral climatic niches, which limits their ability to disperse to regions outside their center of origin (i.e., if they exhibit niche conservatism; Wiens and Donoghue 2004; Wiens et al. 2010).

Most studies exploring the causes of richness gradients do not simultaneously evaluate the roles of past and current climatic conditions, regional species diversification, or evolutionary time, even when it is increasingly recognized that these factors act in combination (Qian et al. 2015; Marin et al. 2018; García‐Andrade et al. 2021, 2023). Instead, many of these works focus on exploring whether regional diversification can explain species richness gradients (independently if driven by climate or not) (Li and Wiens 2019). In contrast, few also consider the influence of evolutionary time or the potential direct role of regional environmental conditions (Qian et al. 2015; Li and Wiens 2019; García‐Andrade et al. 2021; Wu and Wiens 2022). The limited number of studies recognizing these considerations has restricted our ability to reach broader conclusions about how often climate determines regional species richness through its influence on diversification rates or evolutionary times. As a result, our knowledge also remains limited about the factors potentially determining when this occurs, such as the different events shaping the evolutionary history of lineages and their dispersal abilities.

Here, we simultaneously evaluated six hypotheses (listed and detailed in Table 1) explaining the influence of environmental and evolutionary factors shaping the observed patterns of species richness of the snake family Dipsadidae in the Americas. With their > 800 species and 97 genera inhabiting a wide variety of environments from Canada to Argentina and two small genera in Eastern Asia (Uetz et al. 2024), snakes of this family are a good model for answering questions in evolutionary macroecology (Diniz‐Filho 2023). We evaluated whether spatial variation in species richness across the distribution of the Dipsadidae results from the direct impact of regional climate or through its influence on either species accumulation or origination, in agreement with the time and diversification hypotheses, respectively. We implemented a spatial piecewise structural equation model (pSEM) framework combining distributional ranges and a new time‐calibrating phylogeny to test whether environmental and evolutionary factors have left the same signature on the observed pattern of geographical species richness of Dipsadidae in the Americas.

**TABLE 1:** List and description of the main ecological and evolutionary explanations of spatial patterns of species richness.

	Hypothesis	Explanation	Variables	Sources of data or R codes
1	Climate seasonality	The species richness of a region is expected to be inversely proportional to the seasonal change in the temperature or precipitation seasonality it exhibits (Klopfer 1959; Velasco et al. 2018; García‐Andrade et al. 2021)	Temperature seasonality (BIO4) or Precipitation seasonality (BIO15); considered separately	WorldClim 2.0 (Fick and Hijmans 2017)
2	Environmental heterogeneity	The species richness of a region is predicted to be directly proportional to the climatic or spatial heterogeneity it exhibits (Pianka 1966; Velasco et al. 2018; García‐Andrade et al. 2021)	Topographic complexity (Tcomp), as measured with the topographic heterogeneity index (TH8), was used as a proxy for environmental heterogeneity. This variable describes the differences in the topography of a cell compared to that of its neighbors, considering their elevation, slope, and slope aspect (Pelayo‐Villamil et al. 2015)	www.ipez.es/ModestR/
3	Historic climate stability	Regions with more stable climatic conditions in the long term should have increased values of species richness (Araújo et al. 2008; Velasco et al. 2018; García‐Andrade et al. 2021)	The velocity of climate change (rate of climate change over time for the last ~3.3 million years divided by the local rate of climate change across space)	García‐Rodríguez et al. (2025)
4	Environmental productivity hypothesis	Regions with higher values of primary productivity are expected to contain more species (Pianka 1966; Hawkins et al. 2003; Gouveia et al. 2013)	Actual evapotranspiration rate (AET), used as proxy of primary productivity	Trabucco and Zomer (2019)
5	Evolutionary time	Regions accumulating species for longer timespans are predicted to harbor more species (Stephens and Wiens 2003; Wiens 2011; García‐Andrade et al. 2021)	Clade tip ages estimator of Calderón del Cid et al. (2024)	This work
6	Diversification rate	Regions with higher diversification rates are expected to be more species‐rich (Ricklefs 2004; Mittelbach et al. 2007; Kozak and Wiens 2012)	DR metric of Jetz et al. (2012)	This work

## 2 Materials and Methods

### 2.1 Estimation of Species Richness Values Per Cell

We obtained the distribution maps of the species of the Dipsadidae compiled by the Global Assessment of Reptile Distributions (GARD) from Roll et al. (2017) and then reprojected them to the equal‐earth map projection of Šavrič et al. (2019). To generate the geographical gradient of species richness of Dipsadidae across the Americas, we calculated the number of species inhabiting each cell of a 100 km × 100 km grid, which we had overlayed into the distribution maps. We also removed cells with less than 25% land coverage. Per‐cell species richness values were calculated with the package letsR 5.0 (Vilela and Villalobos 2015) of the statistical software R 4.4.2 (R Development Core Team 2024). The resulting presence‐absence matrix contains a total of 2830 cells and 481 species, corresponding to 56.72% of the total species richness of the Dipsadidae (up to October 2024, Uetz et al. 2024).

### 2.2 Estimation of Ecological and Evolutionary Drivers of Regional Richness

We used proxy variables corresponding to each one of the hypotheses to be tested (listed and detailed in Table 1). Temperature seasonality (BIO4) and precipitation seasonality (BIO15) were obtained from the WorldClim 2.0 repository (Fick and Hijmans 2017). Topographic complexity was estimated as the differences in the topography of a cell compared to that of its neighbors, considering their elevation, slope, and slope aspect (Pelayo‐Villamil et al. 2015). Actual evapotranspiration rate (AET) was used as a proxy of primary productivity and was acquired from Trabucco and Zomer (2019). Historical climate stability was estimated as the velocity of past climate change over the past 3 million years calculated using the 11 time horizons from the PaleoClim database, which include key abrupt climate change events over the past 3 million years (e.g., the Younger Dryas Stadial marking the transition between the Pleistocene and Holocene) and current conditions using a set of 14 bioclimatic variables from the WorldClim database. We followed Sandel et al. (2011) and calculated this metric for each period vs. current conditions. Next, we calculated the average climate change velocity for all periods. We then aggregated (and reprojected) the values of the variables corresponding to each hypothesis to the resolution of the grid cells used to calculate regional species richness.

### 2.3 Phylogeny of the Dipsadidae

We first inferred a Bayesian time‐calibrated phylogenetic tree of the Dipsadidae to calculate the metrics employed in this study. To do so, we compiled all sequences of five mitochondrial (12S, 16S, cyt *b*, ND2, and ND4) and four nuclear (BDNF, c‐*mos*, NT3, and RAG2) genes deposited in GenBank (up until March 7, 2021). We also incorporated sequences from Zaher et al. (2018) that were not uploaded into GenBank. We also included sequences from 25 outgroup species of 14 different families of Alethinophidia. Detailed information on all samples used for this work is available in Table S1. Next, we aligned sequences with the online version of MAFFT v7 (Katoh and Standley 2013) under default parameters, except for the 12S and 16S ribosomal genes, for which we used the Q‐INS‐i algorithm (which considers the secondary structure of RNA sequences, Katoh and Toh 2008).

**FIGURE A1:**
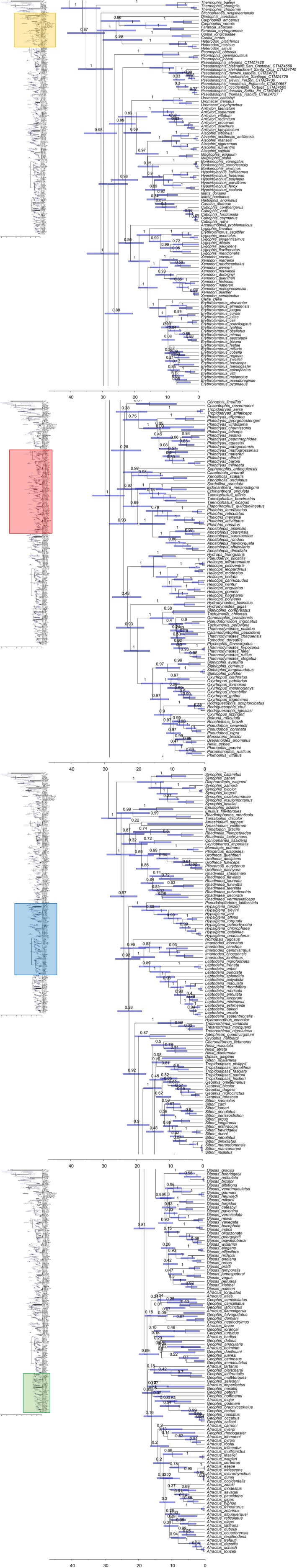
Timetree of the family Dipsadidae derived from a relaxed‐clock MCMC Bayesian analysis using the software BEAST 2, calibrated with the fossils presented in Table A2. The numbers above branches correspond to the posterior probabilities of adjacent nodes. 95% credibility intervals of the age of each node are indicated with blue bars. The units of the scale correspond to millions of years before the present time.

We performed Bayesian phylogenetic analysis (BA) with BEAST 2 v6.3 (Bouckaert et al. 2014) using the gene partition scheme we obtained with the software PartitionFinder 2 (Lanfear et al. 2016; see Appendix A for further methodological details). The sampling of the BEAST 2 analysis used two independent MCMC chains of 100 million generations each, saving one sampled tree per 1000 generations and with the first 50% of trees discarded as burn‐in. The posterior distribution of trees was summarized as a Maximum Clade Credibility (MCC) timetree using TreeAnnotator v2.4.8, part of BEAST 2. The resulting calibrated phylogeny is shown in Figure A1. See Appendix A and Tables A1 and A2 for further details.

Finally, we incorporated the 422 species not included in this phylogeny with the software TACT (Taxonomic Addition for Complete Trees, Chang et al. 2020) following the methodology described in Appendix A (see Table S2 for further details). The resulting 100 fully imputed phylogenies were then used to account for the incomplete taxonomic sampling of our original Bayesian timetree. In the end, both distribution and phylogenetic data were available for 272 species (32.07%).

### 2.4 Calculation of Estimates of Evolutionary Times and Speciation Rates

To estimate the per‐species speciation rate of the taxa included in this study, we used the DR metric of Jetz et al. (2012), also known as the DivRate (Oliveira et al. 2016) or the *λ*_DR_ metric (Title and Rabosky 2019). For a given terminal in a phylogenetic tree, the DR metric measures the rate of lineage origination across all the branches leading to it from the root, giving greater weight to more recent branches (Jetz et al. 2012; Title and Rabosky 2019). As a result, high DR values are observed in species from clades that branch more frequently (Jetz et al. 2012). We used the R function DivRate from Oliveira et al. (2016) to calculate the per‐species DR metric using each of the 100 phylogenies we had obtained with TACT and then averaged the resulting values. Per‐cell values of the DR metric were then obtained by calculating the mean per‐species values across all the species inhabiting each cell of the study area. Although originally considered to measure per‐species net diversification rates, it has been shown that the DR metric performs better as a speciation rate estimator (Quintero and Jetz 2018; Title and Rabosky 2019). In this regard, the accuracy of the DR metric in predicting simulated values of either per‐species diversification or speciation rates is better than other phylogenetic metrics (Oliveira et al. 2016; Title and Rabosky 2019). Further, the DR metric has a similar performance to the tip‐diversification or speciation rates estimated with model‐based approaches implemented in software like BAMM (Rabosky 2014), especially when extinction rates are low (Title and Rabosky 2019). However, we decided to use the DR metric for the analyses performed in this study, as the accuracy of the diversification rates obtained with software like BAMM is limited by the inherent difficulty of estimating the extinction rates used for their calculation (Rabosky 2016; Louca and Pennell 2020). In any case, we repeated all analyses we performed with the DR metric with per‐species speciation, extinction, and net diversification rates we calculated using BAMM v. 2.5.0 and either the original phylogeny we inferred or one of the trees we obtained using TACT selected randomly. See Appendix A for further details regarding the methods used for the BAMM runs and the results obtained.

On the other hand, to obtain an approximation of the time at which members of the Dipsadidae started to inhabit each of the geographic cells covering the study area, we used the clade tip ages estimator of Calderón del Cid et al. (2024). We calculated the clade age values across all the species using each of the 100 completely sampled phylogenies we generated and then averaged the resulting values. The per‐cell clade ages were then obtained by averaging the tip clade ages across all the species present in each cell. Our choice of the clade tip ages estimator to represent evolutionary time follows the results of Calderón del Cid et al. (2024), which found that it was the most adequate for measuring clade age. Clade tip ages values were calculated with the function calculateTipAges of the R package SpeciesAge 0.1.0 (Hauffe et al. 2024). In general, it is important to recognize that the use of phylogenetic metrics in macroevolutionary studies has faced criticism due to their limitations in distinguishing between in situ speciation and colonization (Velasco and Pinto‐Ledezma 2022). For instance, the use of evolutionary time metrics assumes that species reach the total extent of their current distribution immediately after their formation, which is unrealistic. Nonetheless, phylogenetic metrics remain the best approach for assessing the influence of factors driving species richness at the spatial scale of studies such as ours (=grid cells covering an entire continent). That is the case, as inferring dispersal events becomes computationally prohibitive when large numbers of areas and/or species are considered (Matzke 2013; Velasco and Pinto‐Ledezma 2022).

### 2.5 Testing the Drivers of Regional Species Richness Variation

To investigate the potential direct and indirect influences that past and present climatic conditions, regional speciation, and evolutionary time have on cell species richness, we used a spatial piecewise structural equation modeling (pSEM) approach (Skeels et al. 2020). We evaluated a total of 93 pSEM models (listed in Tables A3 and A4, for the models based on trees with taxa imputed with TACT or not, respectively) and selected the one with the lowest corrected Akaike Information Criteria (AICc) value as the best explanation of our data. For all these pSEM models, we treated evolutionary times and speciation rates as endogenous variables (i.e., those whose values can be determined by the other variables of the model). All other variables were considered exogenous (i.e., those known not to result from the remaining variables). Specifically, we considered that each exogenous variable or their combination influenced regional species richness directly or through their action on either one or both endogenous variables. The relationships between species richness and the exogenous variables, as well as between the latter and one or both of the endogenous variables, correspond to the first, second, and third equations of the pSEM models we tested, respectively.

To prevent collinearity, we disregarded variables strongly correlated (=absolute correlation coefficient greater than 0.8) with others from the same pathway of the pSEM models we tested. To perform these correlations, we used the modified version of the *t*‐test proposed by Dutilleul (1993), as implemented in the command modified.ttest of the R package SpatialPack 0.4 (Vallejos et al. 2020). The modified test accounts for the spatial autocorrelation between two spatial processes by adjusting the number of effective degrees of freedom (Dutilleul 1993). Prior to performing these correlations, we normalized the values of all variables (i.e., changed their mean to zero and standard deviation to one) to make the coefficients of the equations of the final pSEM model comparable. Of all the potential predictors of regional species richness of the Dipsadidae we considered, the only strong and significant relationship we recovered was between the values of the DR metric and evolutionary times (Table A5). As a result, we opted to implement and test a set of SEMs that included both per‐cell speciation rates and evolutionary times as endogenous variables, in addition to sets of models that feature each of these variables separately.

For each equation of the pSEM models, we used simultaneous autoregressive models (SARs) to account for spatial autocorrelation between the variables. SARs expand upon Ordinary Least Squares Regressions (OLS) by incorporating an error term that accounts for spatial dependency with a spatial weight matrix. Such a matrix indicates the influence on the residuals corresponding to each site by those of its neighbors (Dormann et al. 2007; Skeels et al. 2020). We inferred the spatial weights matrix corresponding to each equation of the pSEM models using the function nb2listw of the R package spdep (v. 1.3.6; Bivand and Wong 2018), as described in more detail in Appendix A and Table A6. We then incorporated these spatial weight matrices into SARs with the function errorsarlm of the spatialreg package (v. 1.3.5, Bivand et al. 2013) when implementing the pSEM approach.

Finally, to evaluate the goodness of fit of the piecewise structural equation models we defined, we used the command summary.psem from the R package piecewiseSEM (v. 2.3.0., Lefcheck 2016). From the output of this command, we proceeded to sort these models by their values of the corrected Akaike Information Criteria (AICc) (as shown in Tables A7 and A8, for the models based on trees with taxa imputed with TACT or not, respectively). We then selected the model with the lowest AICc, which we proceeded to interpret and graph. All analyses described above were repeated using the original phylogeny of the Dipsadidae we inferred based only on the taxa with phylogenetic data.

## 3 Results

**FIGURE 1:**
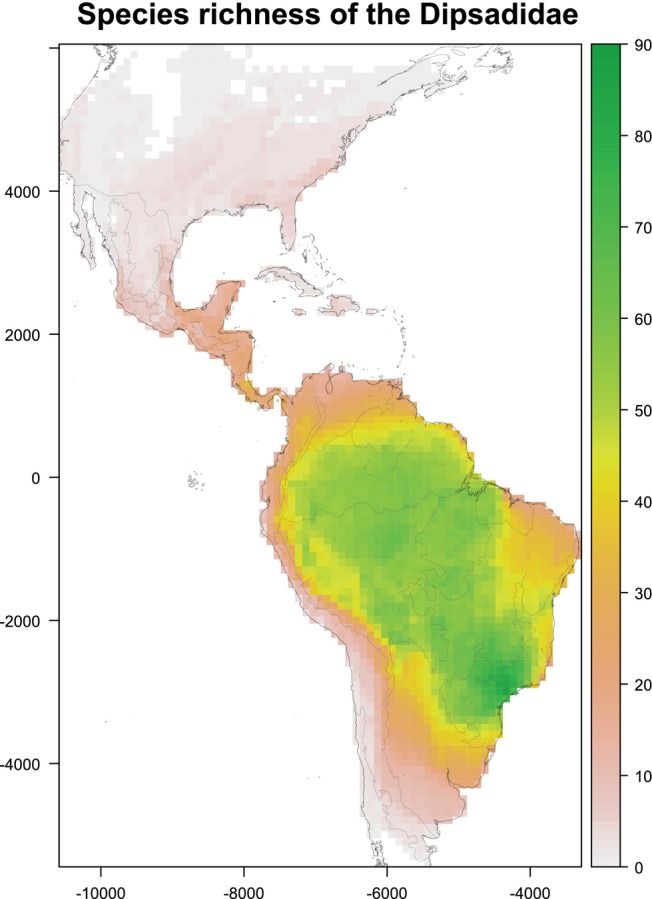
Patterns of species richness of the snake family Dipsadidae across the New World, as estimated from the range maps compiled by Roll et al. (2017). Higher and lower values of species richness of the Dipsadidae are represented with green and white colors, with intermediate values being orange. The map is projected on an equal‐earth projection and overlaid on a 100 km × 100 km grid. Cells with less than 25% land surface are not mapped. Shaded lines represent the biogeographic regions defined by Morrone (2014).

**FIGURE A2:**
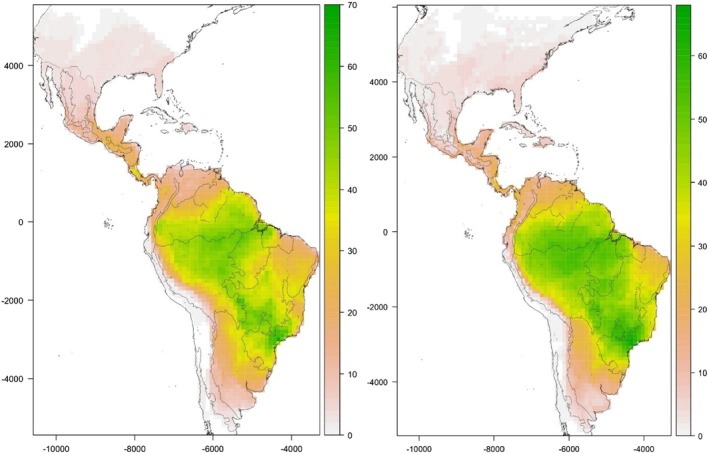
Patterns of species richness of the snake family Dipsadidae across the New World, as estimated from the range maps compiled by Azevedo et al. (2019) and IUCN (2025); to the left and right, respectively. Higher and lower values of species richness of the Dipsadidae are represented with green and white colors, with intermediate values being orange. The map is projected on an equal‐earth projection and overlaid on a 100 km × 100 km grid. Cells with less than 25% land surface are not mapped. Shaded lines represent the biogeographic regions defined by Morrone (2014).

Species richness hotspots in the Dipsadidae concentrate in central and southeastern South America (Figure 1). More specifically, the highest richness values (over 60 species per cell to a maximum of 86) were observed in the Parana dominion of Morrone (2014), followed by most of the Amazonian region (especially in its southern part), where cells have between 50 and 70 species (Figure 1). Many cells between the Cerrado and Rondônia provinces of Morrone (2014) also have richness values of 50–60 species. The remaining parts of the dipsadid distribution have less than 30 species (and fewer than ten species in the cells from the Nearctic and the Prepuna and Monte provinces of Morrone 2014). The only exception is a few cells in northern Costa Rica and the southwestern Andes of Colombia, which have up to 40 species (Figure 1). Similar species richness patterns were observed when using the distribution maps of Azevedo et al. (2019) and IUCN (2025) (Figure A2).

**FIGURE 2:**
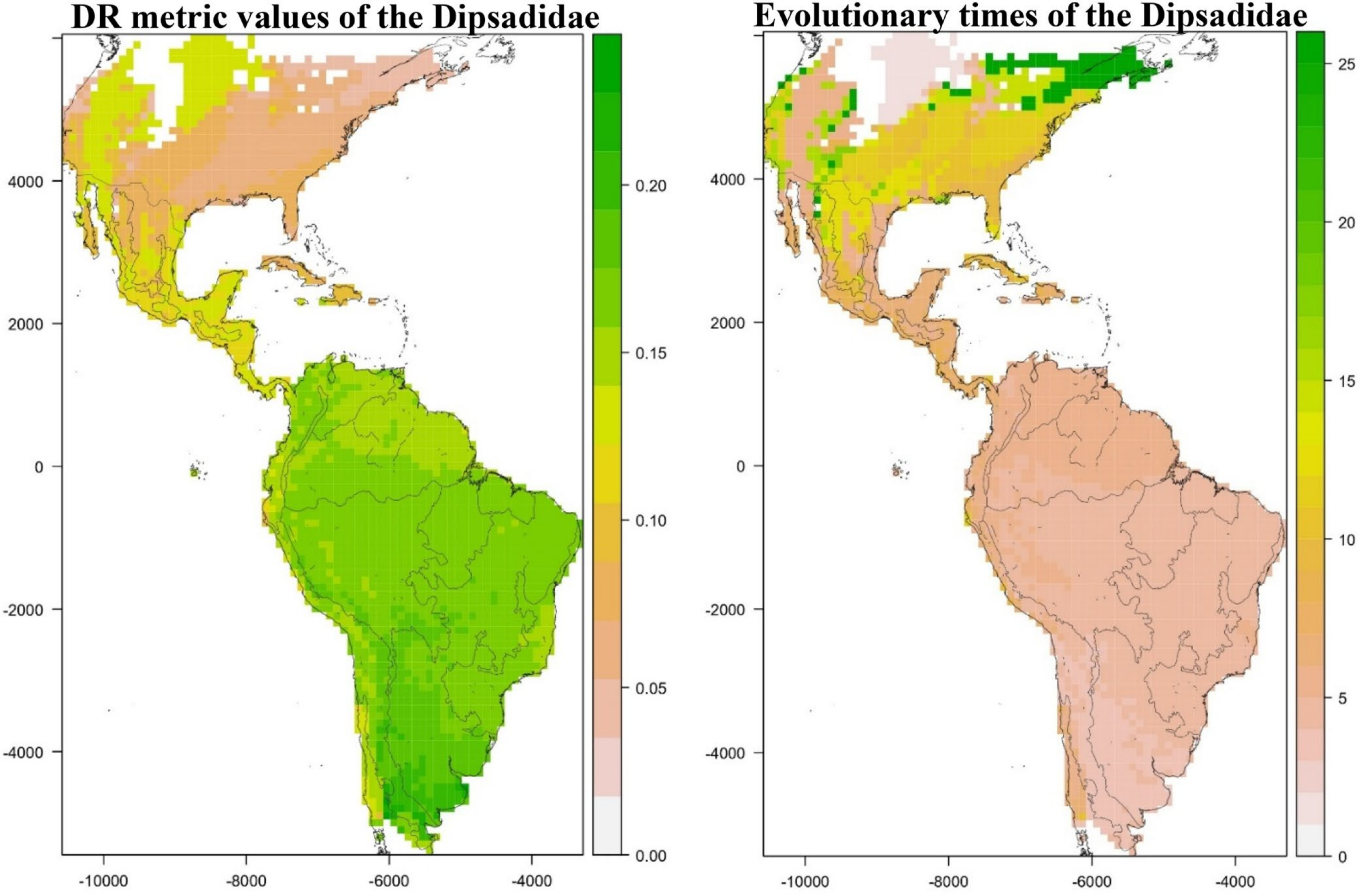
Patterns of geographic variation of the values of per‐cell DR metric (left) and evolutionary times (right) across the distribution of the family Dipsadidae in the New World, as obtained with the 100 fully sampled phylogenies we generated with the stochastic polytomy resolving software TACT. To calculate these fvalues, we used the range maps compiled by Roll et al. (2017). Higher and lower values of species richness of the Dipsadidae are represented with green and white colors, with intermediate values being orange. The map is projected on an equal‐earth projection and overlaid on a 100 km × 100 km grid. Cells with less than 25% of land surface are not mapped. Shaded lines represent the biogeographic regions defined by Morrone (2014).

The mean per‐cell speciation rates do not vary much across the distribution of the group in South America, with the highest values concentrating in the southwest (i.e., in the Chacoan and Pampean provinces of Morrone 2014) (Figure 2). The only exception is a diagonal of low values in Chile and a few cells in the Pacific Coast of Peru. Per‐cell speciation rates are lower across Central America and neotropical Mexico, with the lowest values overall found in most of the Nearctic and the West Indies. However, two areas in North America exhibit speciation rates only slightly lower than those in South America (Figure 2). These hotspots correspond to a diagonal stretching from western Canada to northwestern Mexico (including the northern part of the Baja California peninsula) and another in central Canada and adjacent areas of the United States (Figure 2).

Values of evolutionary times for the cells across the study area are shown in Figure 2. Cells exhibited low evolutionary times over the entire neotropical region, including the West Indies and Baja California peninsula. The Nearctic region generally shows longer evolutionary times than the Neotropics. The highest values are found in southwestern Canada and the adjacent United States, the Pacific Northwest, and isolated areas within the central parts of the United States and northern Mexico (Figure 2). These areas are surrounded by cells with slightly lower values. The remainder of the western United States and Mexico have even lower evolutionary times (although not as low as in the Neotropics). Finally, the diagonal located in western Canada to the United States described above has even lower values, about the same as in the Neotropics (Figure 2).

**FIGURE 3:**
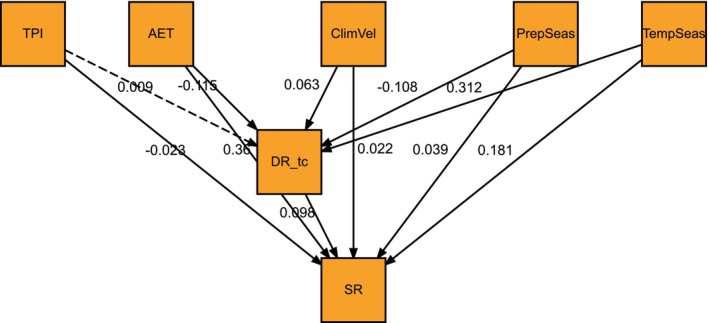
The structural equation model recovered in this work as the best explains the direct and indirect influences of past and current climate, evolutionary times, and speciation rates on the regional species richness of the snake family Dipsadidae. For this model, we used the values of the DR metric obtained using the 100 completely sampled phylogenies we generated with the stochastic polytomy resolving software TACT. Exogenous and endogenous variables of the model are indicated with rectangles and ovals, respectively. Dashed arrows correspond to paths whose coefficient of determination is not significantly different from zero. Abbreviations as follows: *SR* = Species richness, *DR_tc* = speciation rate (as measured with the DR metric and estimated with the phylogenies generated with TACT), *PrepSeas* = current precipitation seasonality, *TempSeas* = current temperature seasonality, TPI = topographic position index, AET = average evapotranspiration, ClimVel = velocity of climate change (=historic climate change).

**FIGURE A3:**
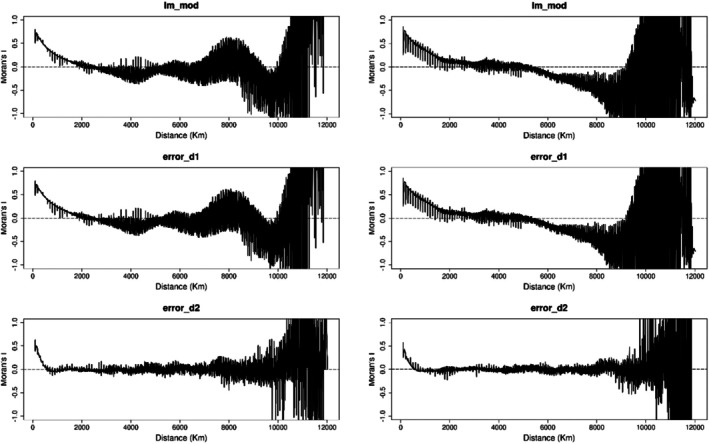
Moran's *I* correlograms indicating spatial autocorrelation in the residuals of the equations of the piecewise structural equation model we evaluated herein. For these equations, we used the values of the DR metric and evolutionary times obtained using one of the 100 completely sampled phylogenies we generated with the stochastic polytomy resolving software TACT and the range maps compiled by Roll et al. (2017). For each of the equations of the model (the first equation at the left and the second at the right), we present the correlograms corresponding to the original Ordinary Least Squares (OLS) model and a spatial autoregressive model (SAR), performed using two weighted schemes (“error_d1” and “error_d2”) to account for the effect of spatial autocorrelation. To obtain these results, we used neighboring distances of 100 and 1000 km, respectively (d1 and d2).

Among the pSEM models we tested, the one recovered as having the best fit to the data is shown in Figure 3 and Appendix B. According to this final model, the following variables had the highest absolute values of both direct standardized regression coefficients: actual evapotranspiration (0.360), current temperature seasonality (0.181), and speciation rates (0.098). These results indicate that actual evapotranspiration is the best predictor of regional species richness values. Indeed, the direct association between these variables is nearly twice as strong as that between current temperature seasonality and species richness. The absolute value of the direct effects of the other explanatory variables was smaller than 0.04 (Figure 3, Table A9). In addition to species richness, the only other endogenous variable included in the best‐fitting model is the speciation rates (as measured by the DR metric). The final pSEM model we evaluated accounted for spatial autocorrelation between the variables it included (Figure A3).

**FIGURE A4:**
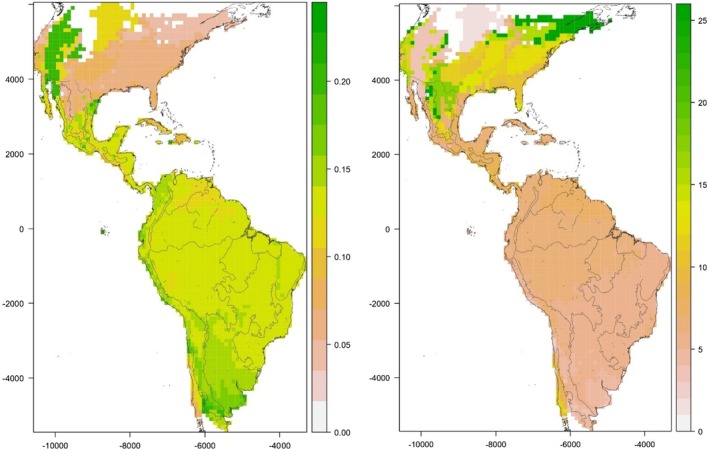
Patterns of geographic variation of the values of per‐cell DR metric (left) and evolutionary times (right) across the distribution of the family Dipsadidae in the New World, obtained using the calibrated Bayesian calibrated phylogeny we inferred herein. To calculate these values, we used the range maps compiled by Roll et al. (2017). Higher and lower values of species richness of the Dipsadidae are represented with green and white colors, with intermediate values being orange. The map is projected on an equal‐earth projection and overlaid on a 100 km × 100 km grid. Cells with less than 25% land surface are not mapped. Shaded lines represent the biogeographic regions defined by Morrone (2014).

**FIGURE A5:**
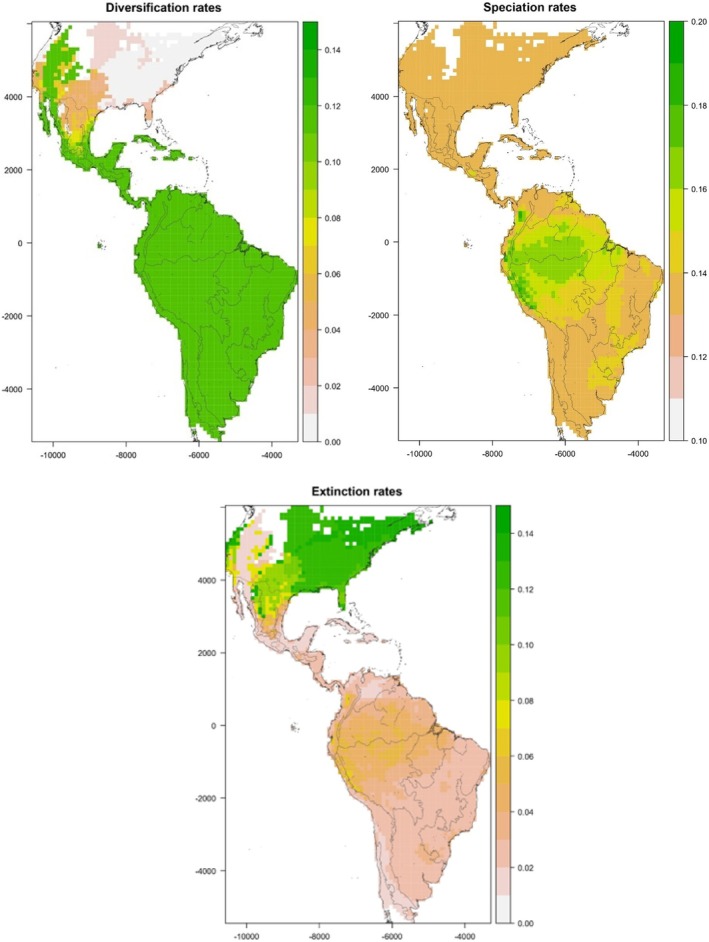
Patterns of geographic variation of the values of per‐cell net diversification, speciation, and extinction rates (left, center, and right, respectively) across the distribution of the family Dipsadidae in the New World, as obtained with the software BAMM. Values were obtained using one of the 100 completely sampled phylogenies we generated with the stochastic polytomy resolving software TACT and the range maps compiled by Roll et al. (2017). Higher and lower values of species richness of the Dipsadidae are represented with green and white colors, with intermediate values being orange. The map is projected on an equal‐earth projection and overlaid on a 100 km × 100 km grid. Cells with less than 25% land surface are not mapped. Shaded lines represent the biogeographic regions defined by Morrone (2014).

**FIGURE A6:**
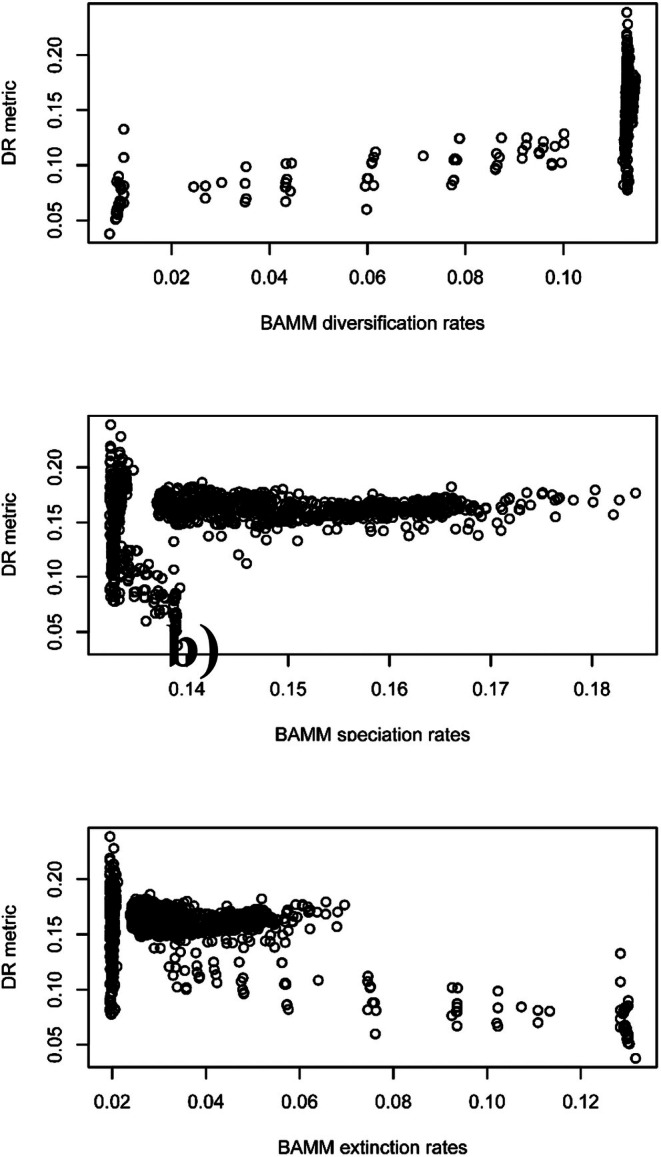
Relationships between the estimated values of the DR metric and the per‐cell net diversification, speciation, and extinction rates, as obtained with the software BAMM and the range maps compiled by Roll et al. (2017). Values of the DR metric were obtained with the 100 completely sampled phylogenies we generated with the stochastic polytomy resolving software TACT. The per‐cell rates we had inferred with BAMM were obtained using one randomly selected tree from those obtained with TACT. After accounting for spatial autocorrelation, the DR metric is only correlated with the per‐cell diversification rates (for net diversification rates: Correlation: 0.846, *F*‐statistic: 5.687 on 1 and 2.251° of freedom, *p*‐value: 0.126; for speciation rates: Dutilleul's modified t‐test, correlation: 0.224, *F*‐statistic: 0.579 on 1 and 10.986° of freedom, *p*‐value: 0.463; for extinction rates: Dutilleul's modified *t*‐test, correlation: −0.807, *F*‐statistic: 5.4747 on 1 and 2.9322° of freedom, *p*‐value: 0.1033).

**FIGURE A7:**
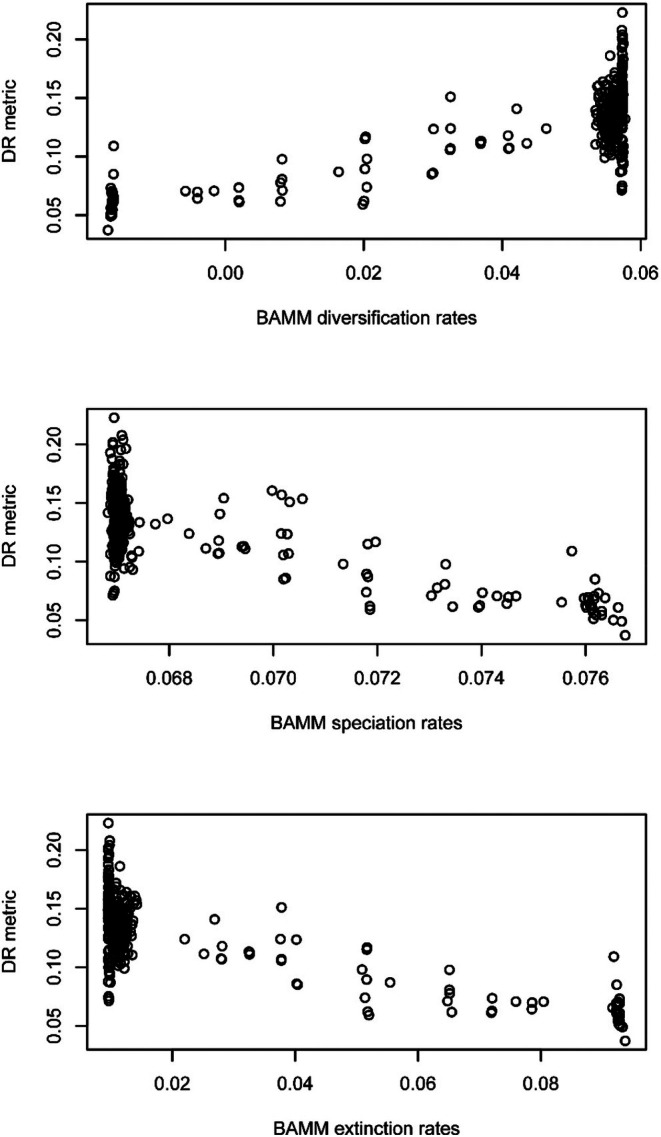
Relationships between the estimated values of the DR metric and those of the per‐cell diversification, speciation, and extinction rates obtained with BAMM. In both cases, we used the Bayesian calibrated phylogeny we inferred herein. To calculate these values, we used the range maps compiled by Roll et al. (2017). After accounting for spatial autocorrelation, the DR metric is only positively correlated with the per‐cell diversification rates (for net diversification rates: Dutilleul's modified *t*‐test, correlation: 0.833, *F*‐statistic: 12.947 on 1 and 5.688° of freedom, *p*‐value: 0.012; for speciation rates: Dutilleul's modified t‐test, correlation: −0.842, *F*‐statistic: 13.981 on 1 and 5.742° of freedom, *p*‐value: 0.010; for extinction rates: Dutilleul's modified t‐test, correlation: −0.8346, *F*‐statistic: 13.0672 on 1 and 5.6926° of freedom, *p*‐value: 0.0122).

Unfortunately, we were unable to estimate Fisher's C global goodness of fit for the selected model or calculate the corresponding d‐separation test values (which indicate if additional variables need to be included in a pathway of the original pSEM model, Shipley 2000; Lefcheck 2016). Such a limitation arises because these metrics cannot be calculated for saturated models (i.e., those assuming there are no missing relationships among the variables of interest, Douma and Shipley 2021), as in the SEM we identified as the best fit for the data. However, we found that the coefficients of determination (*R* ^2^, as in a multiple regression) of the dependent variables of each equation of this model are very high (Species richness with *R* ^2^ = 0.96, speciation rates with *R* ^2^ = 0.89), supporting the idea that it represents the best explanation possible of the data with the variables at hand. Further supporting this assertion, we also observed that the difference in AICc values between the best and second‐best models is 12.1. Values of ΔAICc > 2 between two given models generally indicate that the model with the higher AICc value has substantially worse explanatory potential than the other (Richards 2005; Darriba and Posada 2014). The confirmatory analyses we performed revealed that our results changed only slightly if using the phylogeny we had originally inferred or the rates obtained with BAMM (see Figures A4, A5, A6, A7 and Tables A8 and A10).

## 4 Discussion

We found that actual evapotranspiration and temperature seasonality were the variables that best predicted the geographical species richness pattern in the Dipsadidae, supporting the seasonality and productivity hypotheses over the evolutionary hypotheses for this family of snakes. Indeed, the best‐fitting models indicated a direct causal relationship between these variables and regional species richness. In contrast, all the other environmental variables we considered (=past climate stability, precipitation seasonality, and topographic complexity) had a minimal influence on regional species richness. Similarly, the best‐fitting model showed that the impact of evolutionary factors on species richness was negligible, so neither the diversification rates nor the evolutionary time hypotheses were supported.

**FIGURE A8:**
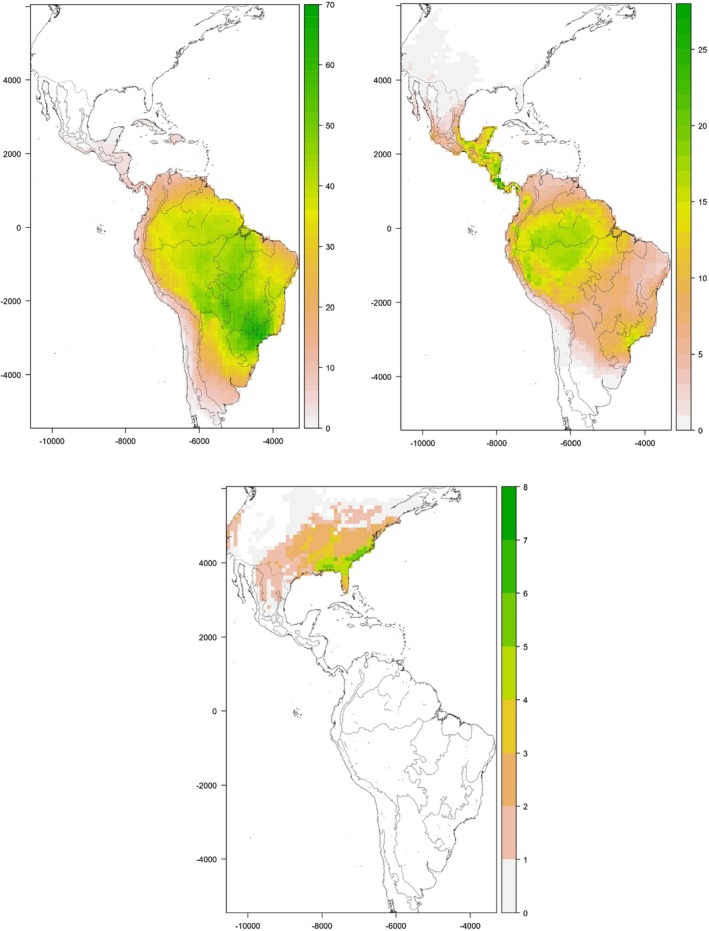
Patterns of species richness of the subfamilies of the snake family Dipsadidae across the New World, as estimated from the range maps compiled by Roll et al. (2017). The left panel shows the distribution of the subfamily Carphophiinae and the mdde and right panels (respectively) to the distribution of the subfamilies Dipsadinae and Xenodontinae, respectively. Higher and lower values of species richness of the Dipsadidae are represented with green and white colors, with intermediate values being orange. The map is projected on an equal‐earth projection and overlaid on a 100 km × 100 km grid. Cells with less than 25% land surface are not mapped. Shaded lines represent the biogeographic regions defined by Morrone (2014).

These results are expected considering the geographic mismatches between the taxonomic and phylogenetic diversity, which we measured using speciation rates and evolutionary times (Ochoa‐Ochoa et al. 2020). Indeed, in contrast to the pattern where species richness peaks in southeastern Brazil and parts of the Amazonian region, speciation rates and evolutionary times are relatively constant across South America and most of the Neotropics. The limited spatial variation of these metrics likely results from the rapid radiation of the predominantly South American subfamily of Dipsadidae (i.e., the Xenodontinae; see Figure A8 for a range map of this group). This is evident in the very short branches and low support values observed in the high‐level relationships within this clade (see Figure A1). While this can be thought of as a limitation of the phylogeny that we inferred here, resulting from the reduced number of loci used, a similar lack of resolution has been obtained in other phylogenies of the Dipsadidae, even when using hundreds of genes or species (Singhal et al. 2017; Ramírez et al. 2023; Serrano et al. 2024).

It is not surprising that the productivity hypothesis had a high explanatory power for the species richness gradients in the Dipsadidae, as it has been frequently reported, both at regional and global scales, in other snake groups (Terribile et al. 2009; Lewin et al. 2016), and in squamates or vertebrates in general (Pianka 1986; Hawkins et al. 2003; Costa et al. 2007; Mouchet et al. 2015; Coops et al. 2018; Barreto et al. 2021). In contrast, previous findings about the relationships between seasonality and species richness in snakes and other vertebrates are mixed, thus limiting our ability to compare them to our results. Indeed, studies based on entire vertebrate groups at continental or global scales do not support the seasonality hypothesis (Rodríguez et al. 2005; Qian and Ricklefs 2008; Howard et al. 2020). In contrast, other works based on the snakes of a given region recover precipitation seasonality as one of the most important predictors of species richness (Costa et al. 2007; de Oliveira and Diniz‐Filho 2010; Moura, Argôlo, and Costa 2017). A potential explanation for this association with rainfall seasonality in snakes can be due to the apparent necessity of ectotherms to be properly hydrated to subsist in environments with elevated temperature variability (Kearney et al. 2013; Moura, Argôlo, and Costa 2017; Moura, Costa, et al. 2017). However, our ability to reach meaningful conclusions about the role of seasonality in driving species richness patterns is limited by the few studies performed to test this hypothesis. Such a reduced number of studies is probably due to the tendency of precipitation and temperature to correlate with their seasonal variation, as at least one of these works has been criticized for not recognizing this limitation (see Raz et al. 2024). It is interesting to note that our finding that temperature seasonality, but not precipitation seasonality, is closely associated with regional species richness was also found in another study on the tribe Lampropeltini of the Colubridae (Pyron and Burbrink 2009). The reason for this discrepancy with previous works is unclear and requires further investigation. A possible explanation could be that Dipsadidae and Lampropeltini have lower water requirements than other snakes, but we are unaware of evidence supporting this possibility.

In general, our findings indicate that the taxonomic diversity of Dipsadidae is mainly influenced by regional climate, with a minimal contribution of diversification and time. Such a result aligns with other studies with similar methodologies focused on different clades and geographical areas (Machac 2020; García‐Andrade et al. 2021). Conversely, the regional species richness of other organisms is primarily determined by evolutionary processes instead (Qian et al. 2015; Gamisch and Comes 2019, and multiple taxa analyzed by Cerezer et al. 2022 and García‐Andrade et al. 2023). Such variation indicates that the drivers of species richness are most likely clade‐specific. In this regard, it has been proposed that finding support for climate as the main factor driving regional richness may be more frequent among taxa with broad distribution ranges, higher dispersion abilities, or reduced niche conservatism (Cerezer et al. 2022). Support for this pattern has been found in several groups of neotropical birds, freshwater fish, and marine mammals (Machac 2020; García‐Andrade et al. 2021, 2023; Cerezer et al. 2022). Our results are consistent with this proposed tendency, as the Dipsadidae is a widespread clade with a distribution range comparable to that of the groups of neotropical birds considered by Machac (2020). However, evidence in other groups is not conclusive. Indeed, the lack of support for the direct role of climate on regional species richness has been observed among lineages with dispersal abilities both high (such as angiosperms and aquatic birds; Qian et al. 2015; Cerezer et al. 2022) and low (as in salamanders and lagomorphs; Cerezer et al. 2022). Further studies in lesser‐known lineages (e.g., marine invertebrates, insects, and plants) are needed to reach more informed general conclusions about the roles of climate and evolutionary processes in driving richness gradients. Hopefully, this will be complemented by investigating how the relative importance of the drivers of regional richness varies within the subgroups of a given clade, known as a deconstructive approach (Marquet et al. 2004; Vásquez‐Restrepo et al. 2023).

However, the idea that climatic conditions can directly determine regional richness is controversial. Some authors argue that environmental conditions alone cannot change regional richness and, therefore, that their influence does not correspond to an alternative to the diversification rate and time hypotheses (Wiens 2011; Qian et al. 2015; Pontarp and Wiens 2017; Wu and Wiens 2022). Simulations and theoretical considerations support the notion that the carrying capacities of the number of species that regions can harbor can only be determined by the processes of speciation, dispersal, and extinction (Wiens 2011; Pontarp and Wiens 2017; Saupe 2023). Conversely, other authors argue that recovering evidence supporting climatic conditions as the main drivers of regional richness (as found by Belmaker and Jetz 2015; Oliveira et al. 2016; and the works cited above) indicates that these conditions directly determine the differences in species carrying capacities between regions (Storch and Okie 2019; Machac 2020; Cerezer et al. 2022). However, it needs to be recognized that results consistent with climate directly driving richness patterns can instead result from the influence of dispersal. Frequent and extensive dispersal events may lead to the rapid assortment of species in regions with different climatic conditions, resulting in the homogenization of their diversification rates and evolutionary times (Qian et al. 2015; Velasco et al. 2018). Unfortunately, determining whether species richness patterns result from a potential direct influence of climatic variables or the role of rapid dispersal is challenging. That is due to the increasing difficulty of reconstructing dispersal events when more regions are considered (Sanmartín 2012; Matzke 2013), particularly for study designs involving hundreds or thousands of areas (=cells) and species, such as ours (Velasco and Pinto‐Ledezma 2022).

Specifically, for the Dipsadidae, the results of previous biogeographic analyses suggest that the lack of support for the diversification rate and time hypotheses may be due to extensive dispersal among its members. Indeed, Ramírez et al. (2023) and Serrano et al. (2024) reported frequent dispersal events throughout the relatively short history of the group in the Americas (~45 mya), with as many as 24 movements between Central and South America and vice versa, for example. Such a result can be observed in that several dipsadid species have extremely broad distributions (e.g., *Clelia clelia* , *Oxyrhopus petola* , *Imantodes cenchoa* , or *Xenodon rabdocephalus;* Köhler 2008; Guedes et al. 2018), which they most likely colonized over very short timespans (less than five mya; Ramírez et al. 2023). In contrast, the dispersal of several dipsadid groups has been limited, as they have a restricted distribution that appears not to have changed in millions of years. Examples of this correspond to the tribes Psomophini, Echinantherini, and Elapomorphini of the Xenodontinae, which are restricted to central and eastern South America (Uetz et al. 2024), and the tribe Diaphorolepini of the Dipsadinae, which is found only in the northern Andes and Darien region of Panama (Pyron et al. 2016). Speciose genera such as *Rhadinaea*, *Rhadinella*, or the polyphyletic *Geophis* are also practically or completely restricted to Central America (Köhler 2008; Guedes et al. 2018). As a result, many cells in this study contain a mix of recent colonizers and species inhabiting them for long timespans and of taxa with high and low diversification rates, as reflected in the reduced variation in evolutionary times and diversification rates across the study area. Even the regions containing taxa from the only diversification rate shift we recovered within the Dipsadidae (i.e., the genus *Atractus*) tend to have similar diversification rates, times, and species richness to elsewhere. These regions correspond mainly to the northern Andes, home to many micro‐endemic *Atractus* species (Pomar‐Gómez et al. 2021).

In summary, the finding that the regional species richness of the Dipsadidae is primarily explained by environmental conditions, with minimal influence from evolutionary processes, provides further evidence consistent with the idea that climate can directly determine richness patterns. Such a result highlights the importance of simultaneously testing the roles of ecological and evolutionary drivers in generating richness gradients (as allowed by methods like structural equation models and variation partitioning), an approach that needs to be implemented more widely. Future studies also need to recognize how the influence of dispersal and extinction on richness gradients may affect their results. Although accounting for these factors remains challenging, we hope this will change soon with the development of new methodologies or with better estimates of extinction rates from fossil evidence. In general, performing similar studies to ours in other taxa and geographical settings would allow us to evaluate how commonly regional climate may have a direct role in determining richness patterns and to identify potential tendencies about the systems where this occurs. These generalities include the possibility that clades for which climate is the primary driver of regional richness are more widespread and possess higher dispersal abilities, as previously mentioned. Another proposed tendency suggests that diversification rates predict regional richness better than evolutionary time among older and more species‐rich clades (Li and Wiens 2019). Finding support for these or other tendencies can provide valuable insights into the origins of shared distribution patterns, such as the latitudinal or altitudinal diversity gradients. Doing so would also allow us to draw broader conclusions about how richness patterns could be affected by the impacts of human activities on future climate.

## Author Contributions

**Juan P. Ramírez:** conceptualization (lead), formal analysis (lead), investigation (lead), methodology (equal), writing – original draft (lead), writing – review and editing (equal). **Julián A. Velasco:** conceptualization (supporting), investigation (supporting), methodology (equal), supervision (equal), writing – original draft (supporting), writing – review and editing (equal). **Tod W. Reeder:** conceptualization (supporting), formal analysis (supporting), investigation (supporting), methodology (supporting), supervision (equal), writing – original draft (supporting), writing – review and editing (supporting).

## Conflicts of Interest

The authors declare no conflicts of interest.

## Supporting information


**Table S1.** Accession numbers of the sequences used for constructing the timetree employed for this work.
**Table S2.** Taxonomic arrangement of the Dipsadidae used for the TACT analyses performed for this work, following Uetz et al. (2024); References used for constructing Table S2.

## Figures and Tables

**FIGURE A9:**
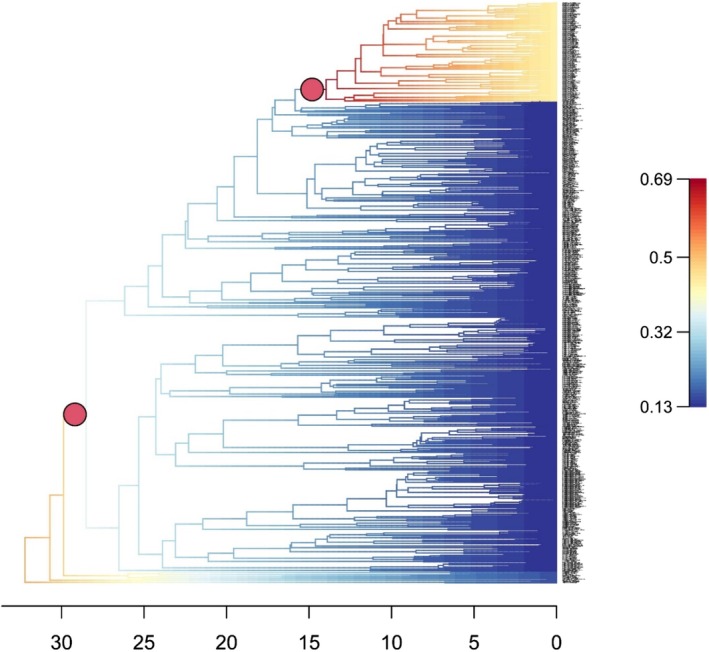
Timetree of the family Dipsadidae with branches colored by the values of net diversification rates (in units of new lineages per million years) estimated with the software BAMM. For this analysis, we accounted for the effect of incomplete taxa sampling by using a randomly selected phylogeny of the 100 completely sampled timetrees of the Dipsadidae we obtained with the software TACT. The red circle indicates the most supported shift in the diversification rates within the Dipsadidae. Warmer and colder colors correspond to higher and lower diversification rates, respectively.

**FIGURE A10:**
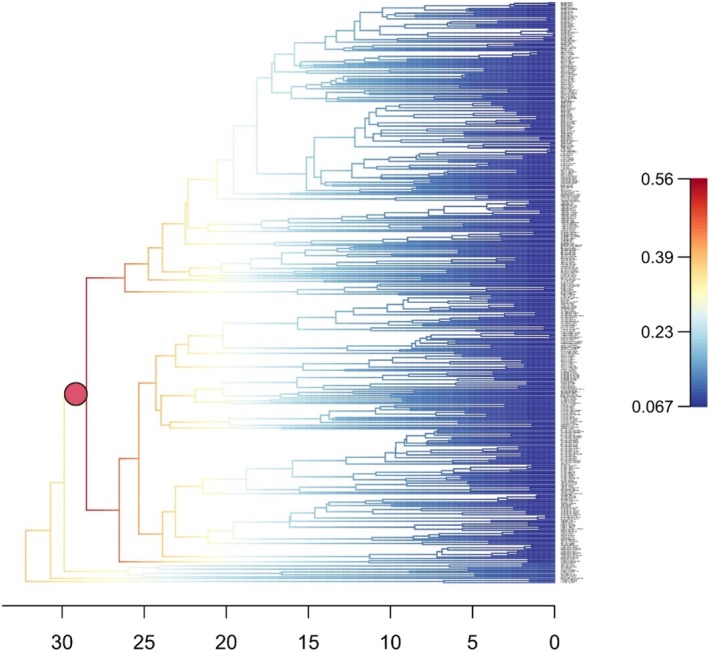
Timetree of the family Dipsadidae with branches colored by the values of net diversification rates (in units of new lineages per million years) estimated with the software BAMM. For this analysis, we did not account for the effect of incomplete taxa sampling. The red circle indicates the most supported shift in the diversification rates within the Dipsadidae. Warmer and colder colors correspond to higher and lower diversification rates, respectively.

## Data Availability

The authors confirm that the data supporting the findings of this study are available within its appendices and Supporting Information. The code used in this work is available at: https://github.com/jpramirez10/Files_Ramirez_et_al_EcoEvol_SRic_Dipsadidae.git.

## Appendix A Extended Methods

### Phylogeny of the Dipsadidae and Incorporation of Taxa Lacking Genetic Data

We first inferred a Bayesian time‐calibrated phylogenetic tree of the Dipsadidae to calculate the metrics employed in this study. To do so, we compiled all sequences of five mitochondrial (12S, 16S, cyt *b*, ND2, and ND4) and four nuclear (BDNF, c‐*mos*, NT3, and RAG2) genes deposited in GenBank (up until March 7, 2021). We also incorporated sequences from Zaher et al. (2018) that were not uploaded into GenBank. We also included sequences from 25 outgroup species of 14 different families of Alethinophidia. Sequence curation involved the deletion of ambiguously aligned positions of the 12S and 16S ribosomal genes. Further, sequences of terminals in phylogenetically unexpected places (or associated with very long branch lengths) were compared with GenBank sequences using the Basic Local Alignment Search Tool (BLAST) (Altschul et al. 1990), and those erroneously assigned to species were filtered. After deleting incorrectly identified sequences, we concatenated all remaining sequences with Sequence Matrix v1.7.6 (Vaidya et al. 2011). Detailed information on all samples used for this work is available in Table S1.

We then aligned sequences with the online version of MAFFT v7 (Katoh and Standley 2013) under default parameters, except for the 12S and 16S ribosomal genes, for which we used the Q‐INS‐i algorithm (which considers the secondary structure of RNA sequences, Katoh and Toh 2008). The final aligned matrix consists of 6038 nucleotide positions and a total of 437 species, of which 413 are dipsadids. These species represent 48.7% of the total richness of the group (Uetz et al. 2024). The software PartitionFinder 2 (Lanfear et al. 2016) was used to select the best‐fitting partition scheme for the alignment according to the corrected Akaike Information Criterion (AICc) and the greedy algorithm of Lanfear et al. (2012). Partitions were defined a priori by gene identity and codon position for protein‐coding genes (all genes except 12S and 16S). The best partitioning scheme for each subset is shown in Table A1.

We performed BA with BEAST 2 v6.3 (Bouckaert et al. 2014) using the merged partitions and models of sequence evolution we obtained with PartitionFinder 2. The posterior distribution of trees, including dates and rates, was obtained by assuming a pure‐birth or Yule model tree prior (a diversification model considering extinction rates negligible compared to speciation rates; Yule 1924; Ritchie et al. 2017). A relaxed normal clock model of evolution was used and calibrated with five fossil calibration points (detailed in Table A2), as well as with the nuclear and mitochondrial rates of Daza et al. (2009). As a starting tree for this analysis, we used the best Maximum Likelihood (ML) phylogeny we obtained on the concatenated dataset using the software RAXML‐HPC V.8.2.12 (Stamatakis 2014), as implemented in the CIPRES Science Gateway v. 3.3 (Miller et al. 2010). To obtain this phylogeny, we performed 20 alternative ML runs, each on a different starting tree, using the GTRCAT model of sequence evolution for all partitions. Node support was established with 1000 bootstrap replicates. The resulting phylogeny was then converted to an ultrametric tree using the previously mentioned calibration points and the nonparametric rate smoothing method implemented in the function ‘chronos’ of the R package ape 5.5 (Paradis and Schliep 2019). The sampling of the BEAST 2 analysis used two independent MCMC chains of 100 million generations each, saving one sampled tree per 1000 generations and with the first 50% of trees discarded as burn‐in. Stationarity and mixing of the log‐likelihood values and parameter estimates were evaluated with Tracer v1.6 (Rambaut et al. 2018) and corroborated by verifying all parameters of the post‐burn‐in set of trees had effective sample sizes > 200. The posterior distribution of trees was summarized as a MCC timetree using TreeAnnotator v2.4.8, part of the BEAST 2 package. The resulting calibrated phylogeny is shown in Figure A1.

We incorporated the 422 species not included in this phylogeny with the software TACT (Taxonomic Addition for Complete Trees, Chang et al. 2020). TACT is a stochastic polytomy resolution algorithm that first evaluates whether user‐defined taxonomic groups are recovered as monophyletic in a phylogeny. Then, it sorts taxa without phylogenetic data to the least‐inclusive monophyletic group they are thought to belong to and assigns them to a random position within that clade (Chang et al. 2020). TACT also estimates the diversification rates of these monophyletic groups, which are used to generate waiting times for the unsampled species (Chang et al. 2020). To account for the stochastic variation in the position of unsampled taxa, we generated 100 completely sampled phylogenies of the Dipsadidae with TACT. To do so, we followed the taxonomy of Uetz et al. (2024) and assigned taxa to subfamily, tribe, and genera. However, we changed the taxonomic assignment of a few taxa recovered in unexpected positions (i.e., conflicting with all previous phylogenies) in the original calibrated Bayesian phylogeny we had inferred (see Table S2 for further details). In the end, both distribution and phylogenetic data were available for 272 species (32.07%).

### Additional Methodological Details About Testing the Drivers of Regional Species Richness Variation

To prevent collinearity, for each equation of the pSEM models we tested, we disregarded variables found to be strongly correlated (=absolute correlation coefficient greater than 0.8) to other variables from the same pathway. To perform these correlations, we used the modified version of the *t*‐test proposed by Dutilleul (1993), as implemented in the command modified.ttest of the R package SpatialPack 0.4 (Vallejos et al. 2020). The modified test accounts for the spatial autocorrelation between two spatial processes by adjusting the number of effective degrees of freedom (Dutilleul 1993). Prior to performing these correlations, we normalized the values of all variables (i.e., changed their mean to zero and standard deviation to one) to make the coefficients of the equations of the final pSEM model comparable. Of all the potential predictors of regional species richness of the Dipsadidae we considered, the only strong and significant relationship we recovered was between the values of the DR metric and evolutionary times (Table A5). As a result, we opted to implement and test a set of SEMs that included both per‐cell speciation rates and evolutionary times as endogenous variables, in addition to sets of models that feature each of these variables separately. The correlations between all variables considered for inclusion in the models we tested are shown in Table A5.

For each equation of the pSEM models, we used simultaneous autoregressive models (SARs) to account for spatial autocorrelation between the variables. SARs expand upon Ordinary Least Squares Regressions (OLS) by incorporating an error term that accounts for spatial dependency with a spatial weight matrix. Such a matrix indicates the influence on the residuals corresponding to each site by those of its neighbors (Dormann et al. 2007; Skeels et al. 2020). We inferred the spatial weights matrix corresponding to each equation of the pSEM models by employing three weighted schemes: Row standardized (W), globally standardized (C), and variance‐stabilizing (S), and using a distance of 100 km between the centroids of grid cells. We also repeated the estimation of the spatial weight matrices using these three weighted schemes but using 1000 km instead (=the maximum distance between centroids). Finally, we determined which of the six resulting spatial weights matrices (plus an OLS regression model) best accounts for the spatial autocorrelation of the variables in each equation of interest by comparing the corresponding values of the Akaike Information Criterion (AIC) and Nagelkerke pseudo‐*R* ^2^. We then used the best‐fitting spatial weights matrices corresponding to each equation (as shown in Table A6) when implementing the pSEM approach. We used the function nb2listw of the R package spdep (v. 1.3.6; Bivand and Wong 2018) for calculating the spatial weight matrices, which we then incorporated into SARs with the function errorsarlm of the spatialreg package (v. 1.3.5, Bivand et al. 2013).

### Methods and Results of the BAMM Analyses We Performed

We used the software BAMM 2.5.0 (Rabosky 2014) to obtain values of the per‐species speciation, extinction, and net diversification rates of the taxa included in this study. To do so, we performed BAMM analyses both with the original Bayesian calibrated timetree we estimated herein and a randomly selected tree of the 100 phylogenies obtained with TACT. The resulting values per‐species rates were then averaged across the taxa inhabiting each cell and compared with the per‐cell DR metric values we had previously obtained (Figures A6 and A7, respectively). In general, BAMM allows the simultaneous inference of the total number of shifts in evolutionary rate regimes found across the dipsadid phylogenetic tree we inferred, as well as the extinction and diversification rates occurring between each shift. The Markov Chain Monte Carlo process implemented in BAMM allows estimating the posterior distribution of likely evolutionary rate regimes (=a mixture of density‐dependent and constant diversification rates) and the number of shifts occurring among them (Rabosky 2014; Seeholzer et al. 2017). The BAMM analysis we performed was run for ten million generations after identifying appropriate priors with the setBAMMpriors function of the R package BAMMtools 2.1.7 (Rabosky et al. 2014). We did not use the sampling fraction option of BAMM for either of the two separate analyses we performed. The convergence of the BAMM analyses was determined by verifying that the effective sample size (ESS) of the log‐likelihood was greater than 200. We also followed the guidelines described in http://bamm‐project.org for ensuring adequate BAMM runs by exploring the credible set of shift configurations and computing marginal odds ratios. We used BAMMtools for visualizing the estimated evolutionary regime shifts and diversification rates across the input tree. Further, we obtained the speciation, extinction, and diversification rates of each species of the input trees by using the getTipRates function of BAMMtools.

The results of the BAMM analysis we performed with the randomly selected phylogeny of the Dipsadidae we had obtained with TACT indicate that the diversification of the group is characterized by two rate regime shifts. The first of these shifts corresponds to a decrease in the diversification rates occurring at the base of a clade formed by the Dipsadinae and Xenodontinae (Figure A9). This shift occurred about 29.87 million years ago (mya) (33.58–24.05, 95% confidence interval) during the Oligocene (Figure A1). The mean speciation rate for the MRCA of the Dipsadinae and the Xenodontinae (0.187 species/millions of years, 0.167–0.213, 95% highest posterior density) is 0.865 times the rate for the remaining members of Dipsadidae (0.217 species/millions of years, 0.107–0.406, 95% highest posterior density). The mean extinction rate for the Dipsadinae + Xenodontinae (0.044 species/millions of years, 0.013–0.083 of 95% highest posterior density) is 0.112 times the corresponding rate for the remaining members of the family (0.135 species/millions of years, 0.002–0.434 of 95% highest posterior density). On the other hand, the other rate shift we recovered in this BAMM analysis corresponds to a clade containing the bulk of the genus *Atractus* (Figure A9). The placement of the few species of this genus not recovered as part of this group most likely corresponds to artifacts due to limited data availability, as is further discussed in Table S2. This shift occurred about 13.67 million years ago (mya) (16.29–10.66, 95% confidence interval) during the Oligocene (Figure A9). In any case, the mean speciation rate of *Atractus* (0.442 species/millions of years, 0.302–0.625, 95% highest posterior density) is 2.72 times the rate for the remaining members of Dipsadidae (0.162 species/millions of years, 0.145–0.185, 95% highest posterior density). The mean extinction rate of *Atractus* (0.259 species/millions of years, 0.036–0.522 of 95% highest posterior density) is 10.1 times the corresponding rate for the remaining members of the family (0.026 species/millions of years, 0.002–0.063 of 95% highest posterior density).

On the other hand, the BAMM analyses we performed with the Bayesian timetree we inferred without including taxa without genetic data only recovered evidence for the same rate regime shift located at the base of a clade formed by the Dipsadinae and Xenodontinae (Figure A10). However, in this case, this shift corresponds to an increase in the diversification rates of the group. The mean speciation rate for the MRCA of the Dipsadinae and the Xenodontinae (0.115 species/millions of years, 0.101–0.132, 95% highest posterior density) is 0.72 times the rate for the remaining members of Dipsadidae (0.160 species/millions of years, 0.067–0.294, 95% highest posterior density). On the other hand, the mean extinction rate for this clade (0.012 species/millions of years, 0.001–0.039 of 95% highest posterior density) is ~1/8 times the corresponding rate for the remaining members of the family (0.098 species/millions of years, 0.001–0.309 of 95% highest posterior density). Finally, for both BAMM analyses, we extracted the values of the per‐species diversification, speciation, and extinction rates of every species included in this study (results not shown).

In general, mapping the per‐cell diversification rates obtained from BAMM results in a geographic pattern similar to the DR metric described in the Results section but with some differences (Figure A4). More specifically, the BAMM diversification rates remain mostly unchanged across the Neotropics, including the West Indies. Much of the western United States and Canada show values comparable to those in the Neotropics (Figure A4). The coastal areas, however, exhibit lower diversification rates, similar to those found in the Central United States and Mexico, as well as in Florida. The western part of the United States has the lowest BAMM diversification rates overall (Figure A4). Surprisingly, the per‐cell speciation rates obtained with BAMM have a very different distribution, with the highest values located in the Amazonian region and parts of the Central and northwestern Andes (Figure A4). The lowest speciation rates are found in Central America, the West Indies, northwestern Colombia (and adjacent Venezuela), the central part of the United States and Canada (except for the coastal region), and the Southern Andes. The remaining regions display intermediate speciation rates (Figure A4). Finally, in the same areas where BAMM speciation rates are at their minimum, the per‐cell extinction rates also reach their lowest values (Figure A4). Higher values can be observed in the rest of South America, with even more elevated extinction rates found in the Central United States and Mexico and along the western coast of the United States and Canada. However, the highest extinction rates overall are concentrated in the eastern United States and Canada, including Florida (Figure A4).

Interestingly, we found that the DR metric and the BAMM speciation rates have a low correlation (when using the phylogeny obtained with TACT) or a strong negative correlation (when using the original tree we had inferred). However, the DR metric and the diversification rates were highly correlated (see Figures A6 and A7, respectively, for more details about these correlations).

## Appendix B Equations Tested in the Best‐Fitted SEM Model

Equations tested in the best‐fitted structural equation model for this work. Abbreviations as in Figure 3:

SR~TPI+AET+ClimVel+PrepSeas+TempSeas+DR

DR~TPI+AET+ClimVel+PrepSeas+TempSeas

**TABLE A1:** Best‐fit partitioning scheme for the protein‐coding genes, and nucleotide substitution models for each partition, based on the corrected Akaike information criterion (AICc) as implemented in PartitionFinder 2 (Lanfear et al. 2016).

Subset	Best subset	Subset partitions	Subset sites
1	GTR + I + G	12S	1–358
2	GTR + I + G	16S	359–871
3	TRNEF+I + G	BDNF_3pos	872–1544/3
4	TVM + I	BDNF_1pos	873–1544/3, 874–1544/3
5	GTR + I + G	ND2_1pos	1545–2288/3
6	GTR + I + G	ND4_2pos	2289–2983/3, 1546–2288/3
7	GTR + I + G	ND2_3pos	1547–2288/3, 3589–4703/3
8	GTR + G	ND4_3pos	2290–2983/3
9	GTR + I + G	ND4_1pos	2291–2983/3
10	TVM + G	cmos_3pos	2984–3586/3, 5327–6038/3
11	TIM + I + G	rag2_1pos	5325–6038/32,985–3586/3
12	TVM + I + G	cmos_2pos	2986–3586/35,326–6038/3
13	GTR + I + G	cytb_1pos	3587–4703/3
14	GTR + I + G	cytb_2pos	3588–4703/3
15	TVM + G	nt3_1pos	4704–5324/3
16	SYM + I + G	nt3_2pos	4705–5324/3
17	TVMEF+G	nt3_3pos	4706–5324/3

*Note:* The abbreviation ‘pos.’ refers to codon position.

**TABLE A2:** Calibration points used for obtaining the timetree used for this work and reported as Figure A1.

	Calibration point	Offset (Ma), minimum age	95% quantile (Ma)	Fossil	References
1	Boidae:Boinae + Erycinae	58	67.5	*Titanoboa cerrejonensis*	Head et al. (2009); Head (2015)
2	stem Caenophidia (= crown Pan‐Caenophidia)	66	88.5	*Krebsophis thobanus*	Rage and Werner (1999); Head et al. (2016)
3	stem Colubroidea (= stem Colubriformes)	50.5	85	*Procerophis sahnii*	Rage et al. (2008) & Head et al. (2016)
4	stem Elapidae	24.95	47.4	Elapidae indeterminate	McCartney et al. (2014); Zaher et al. (2019)
5	crown Viperidae	22.1	31.6	*Vipera cf. V. antiqua*	Zaher et al. (2019)^a^

^a^ According to Head et al. (2016), this fossil cannot be used to calibrate the minimum age of the divergence between Viperidae and its sister group (i.e., Homalopsidae+Colubroidea). Such is the case, as this fossil is of a more recent date than the time of the first appearance of Homalopsidae or Colubroidea in the fossil record. However, the *Vipera* cf. *antiqua* fossil can still be used to calibrate the minimum age of the first divergence within Viperidae (=Azemiopinae vs. Viperinae+Crotalinae), as was done in Zaher et al. (2019) and followed herein.

**TABLE A3:** List of the structural equation models we evaluated in this work. For these models, we used the values of the DR metric and evolutionary times obtained using the 100 completely sampled phylogenies we generated with the stochastic polytomy resolving software TACT. Abbreviations as in Figure 3.

DR_tc_1	SR ~ TPI + DR_tc, DR_tc ~ TPI
DR_tc_2	SR ~ AET + DR_tc, DR_tc ~ AET
DR_tc_3	SR ~ ClimVel + DR_tc, DR_tc ~ ClimVel
DR_tc_4	SR ~ TempSeas + DR_tc, DR_tc ~ TempSeas
DR_tc_5	SR ~ PrepSeas + DR_tc, DR_tc ~ PrepSeas
DR_tc_6	SR ~ TPI + AET + DR_tc, DR_tc ~ TPI + AET
DR_tc_7	SR ~ TPI + ClimVel + DR_tc, DR_tc ~ TPI + ClimVel
DR_tc_8	SR ~ TPI + TempSeas + DR_tc, DR_tc ~ TPI + TempSeas
DR_tc_9	SR ~ TPI + PrepSeas + DR_tc, DR_tc ~ TPI + PrepSeas
DR_tc_10	SR ~ AET + DR_tc + ClimVel, DR_tc ~ AET + ClimVel
DR_tc_11	SR ~ AET + DR_tc + TempSeas, DR_tc ~ AET + TempSeas
DR_tc_12	SR ~ AET + DR_tc + PrepSeas, DR_tc ~ AET + PrepSeas
DR_tc_13	SR ~ ClimVel + DR_tc + TempSeas, DR_tc ~ ClimVel + TempSeas
DR_tc_14	SR ~ ClimVel + DR_tc + PrepSeas, DR_tc ~ ClimVel + PrepSeas
DR_tc_15	SR ~ PrepSeas + DR_tc + TempSeas, DR_tc ~ PrepSeas + TempSeas
DR_tc_16	SR ~ TPI + AET + ClimVel + DR_tc, DR_tc ~ TPI + AET + ClimVel
DR_tc_17	SR ~ TPI + AET + TempSeas + DR_tc, DR_tc ~ TPI + AET + TempSeas
DR_tc_18	SR ~ TPI + AET + PrepSeas + DR_tc, DR_tc ~ TPI + AET + PrepSeas
DR_tc_19	SR ~ TPI + ClimVel + TempSeas + DR_tc, DR_tc ~ TPI + ClimVel + TempSeas
DR_tc_20	SR ~ TPI + ClimVel + PrepSeas + DR_tc, DR_tc ~ TPI + ClimVel + PrepSeas
DR_tc_21	SR ~ TPI + TempSeas + PrepSeas + DR_tc, DR_tc ~ TPI + TempSeas + PrepSeas
DR_tc_22	SR ~ AET + ClimVel + TempSeas + DR_tc, DR_tc ~ AET + ClimVel + TempSeas
DR_tc_23	SR ~ AET + ClimVel + PrepSeas + DR_tc, DR_tc ~ AET + ClimVel + PrepSeas
DR_tc_24	SR ~ AET + TempSeas + PrepSeas + DR_tc, DR_tc ~ AET + TempSeas + PrepSeas
DR_tc_25	SR ~ ClimVel + TempSeas + PrepSeas + DR_tc, DR_tc ~ ClimVel + TempSeas + PrepSeas
DR_tc_26	SR ~ TPI + AET + ClimVel + PrepSeas + DR_tc, DR_tc ~ TPI + AET + ClimVel + PrepSeas
DR_tc_27	SR ~ TPI + AET + ClimVel + TempSeas + DR_tc, DR_tc ~ TPI + AET + ClimVel + TempSeas
DR_tc_28	SR ~ TPI + ClimVel + PrepSeas + TempSeas + DR_tc, DR_tc ~ TPI + ClimVel + PrepSeas + TempSeas
DR_tc_29	SR ~ AET + ClimVel + PrepSeas + TempSeas + DR_tc, DR_tc ~ AET + ClimVel + PrepSeas + TempSeas
DR_tc_30	SR ~ TPI + AET + PrepSeas + TempSeas + DR_tc, DR_tc ~ TPI + AET + PrepSeas + TempSeas
DR_tc_31	SR ~ TPI + AET + ClimVel + PrepSeas + TempSeas + DR_tc, DR_tc ~ TPI + AET + ClimVel + PrepSeas + TempSeas
EvolTime_tc_1	SR ~ TPI + EvolTime_tc, EvolTime_tc ~ TPI
EvolTime_tc_2	SR ~ AET + EvolTime_tc, EvolTime_tc ~ AET
EvolTime_tc_3	SR ~ ClimVel + EvolTime_tc, EvolTime_tc ~ ClimVel
EvolTime_tc_4	SR ~ TempSeas + EvolTime_tc, EvolTime_tc ~ TempSeas
EvolTime_tc_5	SR ~ PrepSeas + EvolTime_tc, EvolTime_tc ~ PrepSeas
EvolTime_tc_6	SR ~ TPI + AET + EvolTime_tc, EvolTime_tc ~ TPI + AET
EvolTime_tc_7	SR ~ TPI + ClimVel+EvolTime_tc, EvolTime_tc ~ TPI + ClimVel
EvolTime_tc_8	SR ~ TPI + TempSeas + EvolTime_tc, EvolTime_tc ~ TPI + TempSeas
EvolTime_tc_9	SR ~ TPI + PrepSeas + EvolTime_tc, EvolTime_tc ~ TPI + PrepSeas
EvolTime_tc_10	SR ~ AET + EvolTime_tc + ClimVel, EvolTime_tc ~ AET + ClimVel
EvolTime_tc_11	SR ~ AET + EvolTime_tc + TempSeas, EvolTime_tc ~ AET + TempSeas
EvolTime_tc_12	SR ~ AET + EvolTime_tc + PrepSeas, EvolTime_tc ~ AET + PrepSeas
EvolTime_tc_13	SR ~ ClimVel + EvolTime_tc + TempSeas, EvolTime_tc ~ ClimVel + TempSeas
EvolTime_tc_14	SR ~ ClimVel + EvolTime_tc + PrepSeas, EvolTime_tc ~ ClimVel + PrepSeas
EvolTime_tc_15	SR ~ PrepSeas + EvolTime_tc + TempSeas, EvolTime_tc ~ PrepSeas + TempSeas
EvolTime_tc_16	SR ~ TPI + AET + ClimVel + EvolTime_tc, EvolTime_tc ~ TPI + AET + ClimVel
EvolTime_tc_17	SR ~ TPI + AET + TempSeas + EvolTime_tc, EvolTime_tc ~ TPI + AET + TempSeas
EvolTime_tc_18	SR ~ TPI + AET + PrepSeas + EvolTime_tc, EvolTime_tc ~ TPI + AET + PrepSeas
EvolTime_tc_19	SR ~ TPI + ClimVel + TempSeas + EvolTime_tc, EvolTime_tc ~ TPI + ClimVel + TempSeas
EvolTime_tc_20	SR ~ TPI + ClimVel + PrepSeas + EvolTime_tc, EvolTime_tc ~ TPI + ClimVel + PrepSeas
EvolTime_tc_21	SR ~ TPI + TempSeas + PrepSeas + EvolTime_tc, EvolTime_tc ~ TPI + TempSeas + PrepSeas
EvolTime_tc_22	SR ~ AET + ClimVel + TempSeas + EvolTime_tc, EvolTime_tc ~ AET + ClimVel + TempSeas
EvolTime_tc_23	SR ~ AET + ClimVel + PrepSeas + EvolTime_tc, EvolTime_tc ~ AET + ClimVel + PrepSeas
EvolTime_tc_24	SR ~ AET + TempSeas + PrepSeas + EvolTime_tc, EvolTime_tc ~ AET + TempSeas + PrepSeas
EvolTime_tc_25	SR ~ ClimVel + TempSeas + PrepSeas + EvolTime_tc, EvolTime_tc ~ ClimVel + TempSeas + PrepSeas
EvolTime_tc_26	SR ~ TPI + AET + ClimVel + PrepSeas + EvolTime_tc, EvolTime_tc ~ TPI + AET + ClimVel + PrepSeas
EvolTime_tc_27	SR ~ TPI + AET + ClimVel + TempSeas + EvolTime_tc, EvolTime_tc ~ TPI + AET + ClimVel + TempSeas
EvolTime_tc_28	SR ~ TPI + ClimVel + PrepSeas + TempSeas + EvolTime_tc, EvolTime_tc ~ TPI + ClimVel + PrepSeas + TempSeas
EvolTime_tc_29	SR ~ AET + ClimVel + PrepSeas + TempSeas + EvolTime_tc, EvolTime_tc ~ AET + ClimVel + PrepSeas + TempSeas
EvolTime_tc_30	SR ~ TPI + AET + PrepSeas + TempSeas + EvolTime_tc, EvolTime_tc ~ TPI + AET + PrepSeas + TempSeas
EvolTime_tc_31	SR ~ TPI + AET + ClimVel + PrepSeas + TempSeas + EvolTime_tc, EvolTime_tc ~ TPI + AET + ClimVel + PrepSeas + TempSeas
DR_ET_tc_1	SR ~ TPI + EvolTime_tc + DR_tc, EvolTime_tc ~ TPI, DR_tc ~ TPI
DR_ET_tc_2	SR ~ AET + EvolTime_tc + DR_tc, EvolTime_tc ~ AET, DR_tc ~ AET
DR_ET_tc_3	SR ~ ClimVel + EvolTime_tc + DR_tc, EvolTime_tc ~ ClimVel, DR_tc ~ ClimVel
DR_ET_tc_4	SR ~ TempSeas + EvolTime_tc + DR_tc, EvolTime_tc ~ TempSeas, DR_tc ~ TempSeas
DR_ET_tc_5	SR ~ PrepSeas + EvolTime_tc + DR_tc, EvolTime_tc ~ PrepSeas, DR_tc ~ PrepSeas
DR_ET_tc_6	SR ~ TPI + AET + EvolTime_tc + DR_tc, EvolTime_tc ~ TPI + AET, DR_tc ~ TPI + AET
DR_ET_tc_7	SR ~ TPI + ClimVel + EvolTime_tc + DR_tc, EvolTime_tc ~ TPI + ClimVel, DR_tc ~ TPI + ClimVel
DR_ET_tc_8	SR ~ TPI + TempSeas + EvolTime_tc + DR_tc, EvolTime_tc ~ TPI + TempSeas, DR_tc ~ TPI + TempSeas
DR_ET_tc_9	SR ~ TPI + PrepSeas+EvolTime_tc + DR_tc, EvolTime_tc ~ TPI + PrepSeas, DR_tc ~ TPI + PrepSeas
DR_ET_tc_10	SR ~ AET + EvolTime_tc + DR_tc + ClimVel, EvolTime_tc ~ AET + ClimVel, DR_tc ~ AET + ClimVel
DR_ET_tc_11	SR ~ AET + EvolTime_tc + DR_tc + TempSeas, EvolTime_tc ~ AET + TempSeas, DR_tc ~ AET + TempSeas
DR_ET_tc_12	SR ~ AET + EvolTime_tc + DR_tc + PrepSeas, EvolTime_tc ~ AET + PrepSeas, DR_tc ~ AET + PrepSeas
DR_ET_tc_13	SR ~ ClimVel + EvolTime_tc + DR_tc + TempSeas, EvolTime_tc ~ ClimVel + TempSeas, DR_tc ~ ClimVel + TempSeas
DR_ET_tc_14	SR ~ ClimVel + EvolTime_tc + DR_tc + PrepSeas, EvolTime_tc ~ ClimVel + PrepSeas, DR_tc ~ ClimVel + PrepSeas
DR_ET_tc_15	SR ~ PrepSeas + EvolTime_tc + DR_tc + TempSeas, EvolTime_tc ~ PrepSeas + TempSeas, DR_tc ~ PrepSeas + TempSeas
DR_ET_tc_16	SR ~ TPI + AET + ClimVel + EvolTime_tc + DR_tc, EvolTime_tc ~ TPI + AET + ClimVel, DR_tc ~ TPI + AET + ClimVel
DR_ET_tc_17	SR ~ TPI + AET + TempSeas + EvolTime_tc + DR_tc, EvolTime_tc ~ TPI + AET + TempSeas, DR_tc ~ TPI + AET + TempSeas
DR_ET_tc_18	SR ~ TPI + AET + PrepSeas + EvolTime_tc + DR_tc, EvolTime_tc ~ TPI + AET + PrepSeas, DR_tc ~ TPI + AET + PrepSeas
DR_ET_tc_19	SR ~ TPI + ClimVel + TempSeas + EvolTime_tc + DR_tc, EvolTime_tc ~ TPI + ClimVel + TempSeas, DR_tc ~ TPI + ClimVel + TempSeas
DR_ET_tc_20	SR ~ TPI + ClimVel + PrepSeas + EvolTime_tc + DR_tc, EvolTime_tc ~ TPI + ClimVel + PrepSeas, DR_tc ~ TPI + ClimVel + PrepSeas
DR_ET_tc_21	SR ~ TPI + TempSeas + PrepSeas + EvolTime_tc + DR_tc, EvolTime_tc ~ TPI + TempSeas + PrepSeas, DR_tc ~ TPI + TempSeas+PrepSeas
DR_ET_tc_22	SR ~ AET + ClimVel + TempSeas + EvolTime_tc + DR_tc, EvolTime_tc ~ AET + ClimVel + TempSeas, DR_tc ~ AET + ClimVel + TempSeas
DR_ET_tc_23	SR ~ AET + ClimVel + PrepSeas + EvolTime_tc + DR_tc, EvolTime_tc ~ AET + ClimVel + PrepSeas, DR_tc ~ AET + ClimVel + PrepSeas
DR_ET_tc_24	SR ~ AET + TempSeas + PrepSeas + EvolTime_tc + DR_tc, EvolTime_tc ~ AET + TempSeas + PrepSeas, DR_tc ~ AET + TempSeas + PrepSeas
DR_ET_tc_25	SR ~ ClimVel + TempSeas + PrepSeas + EvolTime_tc + DR_tc, EvolTime_tc ~ ClimVel + TempSeas + PrepSeas, DR_tc ~ ClimVel + TempSeas + PrepSeas
DR_ET_tc_26	SR ~ TPI + AET + ClimVel + PrepSeas + EvolTime_tc + DR_tc, EvolTime_tc ~ TPI + AET + ClimVel + PrepSeas, DR_tc ~ TPI + AET + ClimVel + PrepSeas
DR_ET_tc_27	SR ~ TPI + AET + ClimVel + TempSeas + EvolTime_tc + DR_tc, EvolTime_tc ~ TPI + AET + ClimVel + TempSeas, DR_tc ~ TPI + AET + ClimVel + TempSeas
DR_ET_tc_28	SR ~ TPI + ClimVel + PrepSeas + TempSeas + EvolTime_tc + DR_tc, EvolTime_tc ~ TPI + ClimVel + PrepSeas + TempSeas, DR_tc ~ TPI + ClimVel + PrepSeas + TempSeas
DR_ET_tc_29	SR ~ AET + ClimVel + PrepSeas + TempSeas + EvolTime_tc + DR_tc, EvolTime_tc ~ AET + ClimVel + PrepSeas + TempSeas, DR_tc ~ AET + ClimVel + PrepSeas + TempSeas
DR_ET_tc_30	SR ~ TPI + AET + PrepSeas + TempSeas + EvolTime_tc + DR_tc, EvolTime_tc ~ TPI + AET + PrepSeas + TempSeas, DR_tc ~ TPI + AET + PrepSeas + TempSeas
DR_ET_tc_31	SR ~ TPI + AET + ClimVel + PrepSeas + TempSeas + EvolTime_tc + DR_tc, EvolTime_tc ~ TPI + AET + ClimVel + PrepSeas + TempSeas, DR_tc ~ TPI + AET + ClimVel + PrepSeas + TempSeas

**TABLE A4:** List of the structural equation models we evaluated in this work. For these models, we used the values of the DR and evolutionary time metrics obtained using the calibrated Bayesian timetree we inferred herein. Abbreviations as in Figure 3, except for *DR* = speciation rate (as measured with the DR metric).

DR_1	SR ~ TPI + DR, DR ~ TPI
DR_2	SR ~ AET + DR, DR ~ AET
DR_3	SR ~ ClimVel + DR, DR ~ ClimVel
DR_4	SR ~ TempSeas + DR, DR ~ TempSeas
DR_5	SR ~ PrepSeas + DR, DR ~ PrepSeas
DR_6	SR ~ TPI + AET + DR, DR ~ TPI + AET
DR_7	SR ~ TPI + ClimVel+DR, DR ~ TPI + ClimVel
DR_8	SR ~ TPI + TempSeas+DR, DR ~ TPI + TempSeas
DR_9	SR ~ TPI + PrepSeas+DR, DR ~ TPI + PrepSeas
DR_10	SR ~ AET + DR + ClimVel, DR ~ AET + ClimVel
DR_11	SR ~ AET+ DR + TempSeas, DR ~ AET + TempSeas
DR_12	SR ~ AET + DR + PrepSeas, DR ~ AET + PrepSeas
DR_13	SR ~ ClimVel + DR + TempSeas, DR ~ ClimVel + TempSeas
DR_14	SR ~ ClimVel + DR + PrepSeas, DR ~ ClimVel + PrepSeas
DR_15	SR ~ PrepSeas + DR + TempSeas, DR ~ PrepSeas + TempSeas
DR_16	SR ~ TPI + AET + ClimVel + DR, DR ~ TPI + AET + ClimVel
DR_17	SR ~ TPI + AET + TempSeas + DR, DR ~ TPI + AET + TempSeas
DR_18	SR ~ TPI + AET + PrepSeas + DR, DR ~ TPI + AET + PrepSeas
DR_19	SR ~ TPI + ClimVel + TempSeas + DR, DR ~ TPI + ClimVel + TempSeas
DR_20	SR ~ TPI + ClimVel + PrepSeas + DR, DR ~ TPI + ClimVel + PrepSeas
DR_21	SR ~ TPI + TempSeas + PrepSeas + DR, DR ~ TPI + TempSeas + PrepSeas
DR_22	SR ~ AET + ClimVel + TempSeas + DR, DR ~ AET + ClimVel + TempSeas
DR_23	SR ~ AET + ClimVel + PrepSeas + DR, DR ~ AET + ClimVel + PrepSeas
DR_24	SR ~ AET + TempSeas + PrepSeas + DR, DR ~ AET + TempSeas + PrepSeas
DR_25	SR ~ ClimVel + TempSeas + PrepSeas + DR, DR ~ ClimVel + TempSeas + PrepSeas
DR_26	SR ~ TPI + AET + ClimVel + PrepSeas + DR, DR ~ TPI + AET + ClimVel + PrepSeas
DR_27	SR ~ TPI + AET + ClimVel + TempSeas + DR, DR ~ TPI + AET + ClimVel + TempSeas
DR_28	SR ~ TPI + ClimVel + PrepSeas + TempSeas + DR, DR ~ TPI + ClimVel + PrepSeas + TempSeas
DR_29	SR ~ AET + ClimVel + PrepSeas + TempSeas + DR, DR ~ AET + ClimVel + PrepSeas + TempSeas
DR_30	SR ~ TPI + AET + PrepSeas + TempSeas + DR, DR ~ TPI + AET + PrepSeas + TempSeas
DR_31	SR ~ TPI + AET + ClimVel + PrepSeas + TempSeas + DR, DR ~ TPI + AET + ClimVel + PrepSeas + TempSeas
EvolTime_1	SR ~ TPI + EvolTime, EvolTime~TPI
EvolTime_2	SR ~ AET + EvolTime, EvolTime~AET
EvolTime_3	SR ~ ClimVel + EvolTime, EvolTime~ClimVel
EvolTime_4	SR ~ TempSeas + EvolTime, EvolTime~TempSeas
EvolTime_5	SR ~ PrepSeas + EvolTime, EvolTime~PrepSeas
EvolTime_6	SR ~ TPI + AET + EvolTime, EvolTime~TPI + AET
EvolTime_7	SR ~ TPI + ClimVel + EvolTime, EvolTime~TPI + ClimVel
EvolTime_8	SR ~ TPI + TempSeas + EvolTime, EvolTime~TPI + TempSeas
EvolTime_9	SR ~ TPI + PrepSeas + EvolTime, EvolTime~TPI + PrepSeas
EvolTime_10	SR ~ AET + EvolTime + ClimVel, EvolTime~AET + ClimVel
EvolTime_11	SR ~ AET + EvolTime + TempSeas, EvolTime~AET + TempSeas
EvolTime_12	SR ~ AET + EvolTime + PrepSeas, EvolTime~AET + PrepSeas
EvolTime_13	SR ~ ClimVel + EvolTime + TempSeas, EvolTime~ClimVel + TempSeas
EvolTime_14	SR ~ ClimVel + EvolTime + PrepSeas, EvolTime~ClimVel + PrepSeas
EvolTime_15	SR ~ PrepSeas + EvolTime + TempSeas, EvolTime~PrepSeas + TempSeas
EvolTime_16	SR ~ TPI + AET + ClimVel + EvolTime, EvolTime~TPI + AET + ClimVel
EvolTime_17	SR ~ TPI + AET + TempSeas + EvolTime, EvolTime~TPI + AET + TempSeas
EvolTime_18	SR ~ TPI + AET + PrepSeas + EvolTime, EvolTime~TPI + AET + PrepSeas
EvolTime_19	SR ~ TPI + ClimVel + TempSeas + EvolTime, EvolTime~TPI + ClimVel + TempSeas
EvolTime_20	SR ~ TPI + ClimVel + PrepSeas + EvolTime, EvolTime~TPI + ClimVel + PrepSeas
EvolTime_21	SR ~ TPI + TempSeas + PrepSeas + EvolTime, EvolTime~TPI + TempSeas + PrepSeas
EvolTime_22	SR ~ AET + ClimVel + TempSeas + EvolTime, EvolTime~AET + ClimVel + TempSeas
EvolTime_23	SR ~ AET + ClimVel + PrepSeas + EvolTime, EvolTime~AET + ClimVel + PrepSeas
EvolTime_24	SR ~ AET + TempSeas + PrepSeas + EvolTime, EvolTime~AET + TempSeas + PrepSeas
EvolTime_25	SR ~ ClimVel + TempSeas + PrepSeas + EvolTime, EvolTime~ClimVel + TempSeas + PrepSeas
EvolTime_26	SR ~ TPI + AET + ClimVel + PrepSeas + EvolTime, EvolTime~TPI + AET + ClimVel + PrepSeas
EvolTime_27	SR ~ TPI + AET + ClimVel + TempSeas + EvolTime, EvolTime~TPI + AET + ClimVel + TempSeas
EvolTime_28	SR ~ TPI + ClimVel + PrepSeas + TempSeas + EvolTime, EvolTime~TPI + ClimVel + PrepSeas + TempSeas
EvolTime_29	SR ~ AET + ClimVel + PrepSeas + TempSeas + EvolTime, EvolTime~AET + ClimVel + PrepSeas + TempSeas
EvolTime_30	SR ~ TPI + AET + PrepSeas + TempSeas + EvolTime, EvolTime~TPI + AET + PrepSeas + TempSeas
EvolTime_31	SR ~ TPI + AET + ClimVel + PrepSeas + TempSeas + EvolTime, EvolTime~TPI + AET + ClimVel + PrepSeas + TempSeas
DR_ET_1	SR ~ TPI + EvolTime + DR, EvolTime~TPI, DR ~ TPI
DR_ET_2	SR ~ AET + EvolTime + DR, EvolTime~AET, DR ~ AET
DR_ET_3	SR ~ ClimVel + EvolTime + DR, EvolTime~ClimVel, DR ~ ClimVel
DR_ET_4	SR ~ TempSeas + EvolTime + DR, EvolTime~TempSeas,DR ~ TempSeas
DR_ET_5	SR ~ PrepSeas + EvolTime + DR, EvolTime~PrepSeas, DR ~ PrepSeas
DR_ET_6	SR ~ TPI + AET + EvolTime + DR, EvolTime ~ TPI + AET, DR ~ TPI + AET
DR_ET_7	SR ~ TPI + ClimVel + EvolTime + DR, EvolTime ~ TPI + ClimVel, DR ~ TPI + ClimVel
DR_ET_8	SR ~ TPI + TempSeas + EvolTime + DR, EvolTime ~ TPI + TempSeas, DR ~ TPI + TempSeas
DR_ET_9	SR ~ TPI + PrepSeas + EvolTime + DR, EvolTime ~ TPI + PrepSeas, DR ~ TPI + PrepSeas
DR_ET_10	SR ~ AET + EvolTime + DR + ClimVel, EvolTime ~ AET + ClimVel, DR ~ AET + ClimVel
DR_ET_11	SR ~ AET + EvolTime + DR + TempSeas, EvolTime ~ AET + TempSeas, DR ~ AET + TempSeas
DR_ET_12	SR ~ AET + EvolTime + DR + PrepSeas, EvolTime ~ AET + PrepSeas, DR ~ AET + PrepSeas
DR_ET_13	SR ~ ClimVel + EvolTime + DR + TempSeas, EvolTime ~ ClimVel + TempSeas, DR ~ ClimVel + TempSeas
DR_ET_14	SR ~ ClimVel + EvolTime + DR + PrepSeas, EvolTime ~ ClimVel + PrepSeas, DR ~ ClimVel + PrepSeas
DR_ET_15	SR ~ PrepSeas + EvolTime + DR + TempSeas, EvolTime ~ PrepSeas + TempSeas, DR ~ PrepSeas + TempSeas
DR_ET_16	SR ~ TPI+ AET + ClimVel+EvolTime + DR, EvolTime~TPI + AET + ClimVel, DR ~ TPI + AET + ClimVel
DR_ET_17	SR ~ TPI+ AET + TempSeas+EvolTime + DR, EvolTime~TPI + AET + TempSeas, DR ~ TPI + AET + TempSeas
DR_ET_18	SR ~ TPI+ AET + PrepSeas+EvolTime + DR, EvolTime~TPI + AET + PrepSeas, DR ~ TPI + AET + PrepSeas
DR_ET_19	SR ~ TPI + ClimVel + TempSeas + EvolTime + DR, EvolTime ~ TPI + ClimVel + TempSeas, DR ~ TPI + ClimVel + TempSeas
DR_ET_20	SR ~ TPI + ClimVel + PrepSeas + EvolTime + DR, EvolTime ~ TPI + ClimVel + PrepSeas, DR ~ TPI + ClimVel + PrepSeas
DR_ET_21	SR ~ TPI + TempSeas + PrepSeas + EvolTime + DR, EvolTime~TPI + TempSeas + PrepSeas, DR ~ TPI + TempSeas + PrepSeas
DR_ET_22	SR ~ AET + ClimVel + TempSeas + EvolTime + DR, EvolTime ~ AET + ClimVel+TempSeas, DR ~ AET + ClimVel + TempSeas
DR_ET_23	SR ~ AET + ClimVel + PrepSeas + EvolTime + DR, EvolTime ~ AET + ClimVel + PrepSeas, DR ~ AET + ClimVel + PrepSeas
DR_ET_24	SR ~ AET + TempSeas + PrepSeas + EvolTime + DR, EvolTime ~ AET + TempSeas + PrepSeas, DR ~ AET + TempSeas + PrepSeas
DR_ET_25	SR ~ ClimVel + TempSeas + PrepSeas + EvolTime + DR, EvolTime ~ ClimVel + TempSeas + PrepSeas, DR ~ ClimVel + TempSeas + PrepSeas
DR_ET_26	SR ~ TPI + AET + ClimVel + PrepSeas + EvolTime + DR, EvolTime ~ TPI + AET + ClimVel + PrepSeas, DR ~ TPI + AET + ClimVel + PrepSeas
DR_ET_27	SR ~ TPI + AET + ClimVel + TempSeas + EvolTime + DR, EvolTime ~ TPI + AET + ClimVel + TempSeas, DR ~ TPI + AET + ClimVel + TempSeas
DR_ET_28	SR ~ TPI + ClimVel + PrepSeas + TempSeas + EvolTime + DR, EvolTime ~ TPI + ClimVel + PrepSeas + TempSeas, DR ~ TPI + ClimVel + PrepSeas + TempSeas
DR_ET_29	SR ~ AET + ClimVel + PrepSeas + TempSeas + EvolTime + DR, EvolTime ~ AET + ClimVel + PrepSeas + TempSeas, DR ~ AET + ClimVel + PrepSeas + TempSeas
DR_ET_30	SR ~ TPI + AET + PrepSeas + TempSeas + EvolTime + DR, EvolTime ~ TPI + AET + PrepSeas + TempSeas, DR ~ TPI + AET + PrepSeas + TempSeas
DR_ET_31	SR ~ TPI + AET + ClimVel + PrepSeas + TempSeas + EvolTime + DR, EvolTime ~ TPI + AET + ClimVel + PrepSeas + TempSeas, DR ~ TPI + AET + ClimVel + PrepSeas + TempSeas

**TABLE A5:** Dutilleul's modified *t*‐test between all explanatory variables considered for the theoretical structural equation model tested for this study.

	Temperature seasonality	Precipitation seasonality	Evolutionary time	Speciation rates (DR metric)	Average evapotranspiration	Velocity of climate change (=historic climate change)
Precipitation seasonality	−0.354 (0.132)	0	0	0	0	0
Evolutionary time	0.526 (0.218)	−0.281 (0.069)	0	0	0	0
Speciation rates (DR metric)	−0.668 (0.322)	0.279 (0.165)	**−0.851 (0.022)**	0	0	0
Average evapotranspiration (AET)	−0.629 (0.212)	−0.239 (0.234)	−0.213 (0.464)	0.266 (0.537)	0	0
Velocity of climate change (=historic climate change)	−0.084 (0.681)	−0.189 (0.281)	−0.04 (0.735)	0.046 (0.754)	0.453 (0.016)	0
Topographic position index (TPI)	−0.051 (0.722)	0.341 (0.009)	0.023 (0.829)	−0.019 (0.883)	−0.36 (0.008)	−0.615 (0)

*Note:* For this model, we used the values of the DR and evolutionary time metrics obtained using the 100 completely sampled phylogenies we generated with the stochastic polytomy resolving software TACT and the range maps compiled by Roll et al. (2017). Values on each cell correspond to the slope of the correlation between each combination of variables, with the corresponding *p*‐values shown in parentheses. Strong correlations (=slopes > 0.8) are indicated in bold.

**TABLE A6:** Model selection of the spatial weight matrices associated with each of the equations of the best‐fitted structural equation model tested herein using the range maps compiled by Roll et al. (2017).

Equation	Scheme	Model	*R* ^2^	AIC value	Degrees of freedom
Equation 1	W	lm_mod	0.875	3570.709	8
	W	error_d1	0.877	3533.042	9
	W	error_d2	0.958	457.216	9
	C	lm_mod	0.875	3570.709	8
	C	error_d1	0.877	3533.042	9
	C	error_d2	0.944	1306.378	9
	S	lm_mod	0.875	3570.709	8
	S	error_d1	0.877	3533.042	9
	S	error_d2	0.955	696.089	9
Equation 2	W	lm_mod	0.528	5913.093	7
	W	error_d1	0.530	5911.933	8
	W	error_d2	0.892	1754.083	8
	C	lm_mod	0.528	5913.093	7
	C	error_d1	0.530	5911.933	8
	C	error_d2	0.832	2999.798	8
	S	lm_mod	0.528	5913.093	7
	S	error_d1	0.530	5911.933	8
	S	error_d2	0.869	2290.101	8

*Note:* For each equation, we compared the model fit of the spatial autoregressive analyses (SARs) we performed using six spatial weight matrices. These matrices correspond to all possible combinations between two neighborhood distances (d1 = 100 km, d2 = 1000 km) and three weight coding schemes (row standardized [“W”], globally standardized [“C”], and variance‐stabilizing [“S”]). For this procedure, we used the values of the DR metric and evolutionary time obtained using the 100 completely sampled phylogenies we generated with the stochastic polytomy resolving software TACT.

**TABLE A7:** List of the structural equation models we evaluated in this work, sorted by their values of the corrected Akaike Information Criteria (AICc). For this model selection procedure, we used the values of the DR metric and evolutionary times obtained using the 100 completely sampled phylogenies we generated with the stochastic polytomy resolving software TACT. Abbreviations as in Figure 3.

	AICc	Equations
DR_ET_tc_3	10,873.57	SR ~ ClimVel + EvolTime_tc + DR_tc, EvolTime_tc ~ ClimVel, DR_tc ~ ClimVel
DR_ET_tc_9	10,685.31	SR ~ TPI + PrepSeas + EvolTime_tc + DR_tc, EvolTime_tc ~ TPI + PrepSeas, DR_tc ~ TPI + PrepSeas
DR_ET_tc_15	10,631.65	SR ~ PrepSeas + EvolTime_tc + DR_tc + TempSeas, EvolTime_tc ~ PrepSeas + TempSeas, DR_tc ~ PrepSeas + TempSeas
DR_ET_tc_21	10,393.25	SR ~ TPI + TempSeas + PrepSeas + EvolTime_tc + DR_tc, EvolTime_tc ~ TPI + TempSeas + PrepSeas, DR_tc ~ TPI + TempSeas + PrepSeas
DR_ET_tc_18	9899.161	SR ~ TPI + AET + PrepSeas + EvolTime_tc + DR_tc, EvolTime_tc ~ TPI + AET + PrepSeas, DR_tc ~ TPI + AET + PrepSeas
DR_ET_tc_12	9719.094	SR ~ AET + EvolTime_tc + DR_tc + PrepSeas, EvolTime_tc ~ AET + PrepSeas, DR_tc ~ AET + PrepSeas
DR_ET_tc_6	9688.536	SR ~ TPI + AET + EvolTime_tc + DR_tc, EvolTime_tc ~ TPI + AET, DR_tc ~ TPI + AET
DR_ET_tc_5	9482.664	SR ~ PrepSeas + EvolTime_tc + DR_tc, EvolTime_tc ~ PrepSeas, DR_tc ~ PrepSeas
DR_ET_tc_27	9412.943	SR ~ TPI + AET + ClimVel + TempSeas + EvolTime_tc + DR_tc, EvolTime_tc ~ TPI + AET + ClimVel + TempSeas, DR_tc ~ TPI + AET + ClimVel + TempSeas
DR_ET_tc_24	9342.45	SR ~ AET + TempSeas + PrepSeas + EvolTime_tc + DR_tc, EvolTime_tc ~ AET + TempSeas + PrepSeas, DR_tc ~ AET + TempSeas + PrepSeas
DR_ET_tc_30	9337.724	SR ~ TPI + AET + PrepSeas + TempSeas + EvolTime_tc + DR_tc, EvolTime_tc ~ TPI + AET + PrepSeas + TempSeas, DR_tc ~ TPI + AET + PrepSeas + TempSeas
DR_ET_tc_14	9267.028	SR ~ ClimVel + EvolTime_tc + DR_tc + PrepSeas, EvolTime_tc ~ ClimVel + PrepSeas, DR_tc ~ ClimVel + PrepSeas
DR_ET_tc_8	9235.958	SR ~ TPI + TempSeas + EvolTime_tc + DR_tc, EvolTime_tc ~ TPI + TempSeas, DR_tc ~ TPI + TempSeas
DR_ET_tc_20	9167.001	SR ~ TPI + ClimVel + PrepSeas + EvolTime_tc + DR_tc, EvolTime_tc ~ TPI + ClimVel + PrepSeas, DR_tc ~ TPI + ClimVel + PrepSeas
DR_ET_tc_11	8293.863	SR ~ AET + EvolTime_tc + DR_tc + TempSeas, EvolTime_tc ~ AET + TempSeas, DR_tc ~ AET + TempSeas
DR_ET_tc_4	8274.158	SR ~ TempSeas + EvolTime_tc + DR_tc, EvolTime_tc ~ TempSeas,DR_tc ~ TempSeas
DR_ET_tc_1	8253.855	SR ~ TPI + EvolTime_tc + DR_tc, EvolTime_tc ~ TPI, DR_tc ~ TPI
DR_ET_tc_2	8207.841	SR ~ AET + EvolTime_tc + DR_tc, EvolTime_tc ~ AET, DR_tc ~ AET
DR_ET_tc_7	8143.136	SR ~ TPI + ClimVel + EvolTime_tc + DR_tc, EvolTime_tc ~ TPI + ClimVel, DR_tc ~ TPI + ClimVel
DR_ET_tc_23	8074.137	SR ~ AET + ClimVel+PrepSeas + EvolTime_tc + DR_tc, EvolTime_tc ~ AET + ClimVel + PrepSeas, DR_tc ~ AET + ClimVel + PrepSeas
DR_ET_tc_13	8071.38	SR ~ ClimVel + EvolTime_tc + DR_tc + TempSeas, EvolTime_tc ~ ClimVel + TempSeas, DR_tc ~ ClimVel + TempSeas
DR_ET_tc_17	8063.186	SR ~ TPI + AET + TempSeas + EvolTime_tc + DR_tc, EvolTime_tc ~ TPI + AET + TempSeas, DR_tc ~ TPI + AET + TempSeas
DR_ET_tc_26	8057.159	SR ~ TPI + AET + ClimVel + PrepSeas + EvolTime_tc + DR_tc, EvolTime_tc ~ TPI + AET + ClimVel + PrepSeas, DR_tc ~ TPI + AET + ClimVel + PrepSeas
DR_ET_tc_19	7986.265	SR ~ TPI + ClimVel + TempSeas + EvolTime_tc + DR_tc, EvolTime_tc ~ TPI + ClimVel + TempSeas, DR_tc ~ TPI + ClimVel + TempSeas
DR_ET_tc_29	7928.106	SR ~ AET + ClimVel+PrepSeas+TempSeas+EvolTime_tc + DR_tc, EvolTime_tc ~ AET + ClimVel+PrepSeas+TempSeas, DR_tc ~ AET + ClimVel+PrepSeas+TempSeas
DR_ET_tc_25	7921.567	SR ~ ClimVel+TempSeas+PrepSeas+EvolTime_tc + DR_tc, EvolTime_tc ~ ClimVel+TempSeas+PrepSeas, DR_tc ~ ClimVel+TempSeas+PrepSeas
DR_ET_tc_28	7846.322	SR ~ TPI+ ClimVel+PrepSeas+TempSeas+EvolTime_tc + DR_tc, EvolTime_tc ~ TPI + ClimVel+PrepSeas+TempSeas, DR_tc ~ TPI + ClimVel+PrepSeas+TempSeas
DR_ET_tc_10	7396.857	SR ~ AET+ EvolTime_tc + DR_tc + ClimVel, EvolTime_tc ~ AET + ClimVel, DR_tc ~ AET + ClimVel
DR_ET_tc_16	7106.743	SR ~ TPI+ AET + ClimVel+EvolTime_tc + DR_tc, EvolTime_tc ~ TPI + AET + ClimVel, DR_tc ~ TPI + AET + ClimVel
DR_ET_tc_22	6999.569	SR ~ AET + ClimVel+TempSeas+EvolTime_tc + DR_tc, EvolTime_tc ~ AET + ClimVel+TempSeas, DR_tc ~ AET + ClimVel+TempSeas
DR_ET_tc_31	6829.612	SR ~ TPI+ AET + ClimVel+PrepSeas+TempSeas+EvolTime_tc + DR_tc, EvolTime_tc ~ TPI + AET + ClimVel+PrepSeas+TempSeas, DR_tc ~ TPI + AET + ClimVel+PrepSeas+TempSeas
EvolTime_tc_5	6325.876	SR ~ PrepSeas+ EvolTime_tc, EvolTime_tc ~ PrepSeas
EvolTime_tc_3	6287.886	SR ~ ClimVel+ EvolTime_tc, EvolTime_tc ~ ClimVel
EvolTime_tc_1	6260.427	SR ~ TPI+ EvolTime_tc, EvolTime_tc ~ TPI
EvolTime_tc_4	6222.962	SR ~ TempSeas+ EvolTime_tc, EvolTime_tc ~ TempSeas
EvolTime_tc_7	6203.362	SR ~ TPI+ ClimVel+EvolTime_tc, EvolTime_tc ~ TPI + ClimVel
EvolTime_tc_13	6191.032	SR ~ ClimVel+ EvolTime_tc + TempSeas, EvolTime_tc ~ ClimVel+TempSeas
EvolTime_tc_14	6176.307	SR ~ ClimVel+ EvolTime_tc + PrepSeas, EvolTime_tc ~ ClimVel+PrepSeas
EvolTime_tc_8	6153.461	SR ~ TPI+ TempSeas+EvolTime_tc, EvolTime_tc ~ TPI + TempSeas
EvolTime_tc_9	6152.984	SR ~ TPI+ PrepSeas+EvolTime_tc, EvolTime_tc ~ TPI + PrepSeas
EvolTime_tc_19	6145.72	SR ~ TPI+ ClimVel+TempSeas+EvolTime_tc, EvolTime_tc ~ TPI + ClimVel+TempSeas
EvolTime_tc_15	6122.98	SR ~ PrepSeas+ EvolTime_tc + TempSeas, EvolTime_tc ~ PrepSeas+TempSeas
EvolTime_tc_20	6103.955	SR ~ TPI+ ClimVel+PrepSeas+EvolTime_tc, EvolTime_tc ~ TPI + ClimVel+PrepSeas
EvolTime_tc_25	6100.117	SR ~ ClimVel+TempSeas+PrepSeas+EvolTime_tc, EvolTime_tc ~ ClimVel+TempSeas+PrepSeas
EvolTime_tc_21	6071.63	SR ~ TPI + TempSeas+PrepSeas+EvolTime_tc, EvolTime_tc ~ TPI + TempSeas+PrepSeas
EvolTime_tc_28	6065.724	SR ~ TPI+ ClimVel+PrepSeas+TempSeas+EvolTime_tc, EvolTime_tc ~ TPI + ClimVel+PrepSeas+TempSeas
EvolTime_tc_2	5394.446	SR ~ AET+ EvolTime_tc, EvolTime_tc ~ AET
EvolTime_tc_12	5346.043	SR ~ AET+ EvolTime_tc + PrepSeas, EvolTime_tc ~ AET + PrepSeas
EvolTime_tc_6	5335.99	SR ~ TPI+ AET + EvolTime_tc, EvolTime_tc ~ TPI + AET
EvolTime_tc_10	5292.094	SR ~ AET+ EvolTime_tc + ClimVel, EvolTime_tc ~ AET + ClimVel
EvolTime_tc_18	5287.379	SR ~ TPI+ AET + PrepSeas+EvolTime_tc, EvolTime_tc ~ TPI + AET + PrepSeas
EvolTime_tc_16	5282.686	SR ~ TPI+ AET + ClimVel+EvolTime_tc, EvolTime_tc ~ TPI + AET + ClimVel
EvolTime_tc_11	5282.602	SR ~ AET+ EvolTime_tc + TempSeas, EvolTime_tc ~ AET + TempSeas
EvolTime_tc_17	5245.822	SR ~ TPI+ AET + TempSeas+EvolTime_tc, EvolTime_tc ~ TPI + AET + TempSeas
EvolTime_tc_23	5244.583	SR ~ AET + ClimVel+PrepSeas+EvolTime_tc, EvolTime_tc ~ AET + ClimVel+PrepSeas
EvolTime_tc_26	5235.131	SR ~ TPI+ AET + ClimVel+PrepSeas+EvolTime_tc, EvolTime_tc ~ TPI + AET + ClimVel+PrepSeas
EvolTime_tc_22	5232.219	SR ~ AET + ClimVel+TempSeas+EvolTime_tc, EvolTime_tc ~ AET + ClimVel+TempSeas
EvolTime_tc_24	5227.435	SR ~ AET + TempSeas+PrepSeas+EvolTime_tc, EvolTime_tc ~ AET + TempSeas+PrepSeas
EvolTime_tc_27	5222.562	SR ~ TPI+ AET + ClimVel+TempSeas+EvolTime_tc, EvolTime_tc ~ TPI + AET + ClimVel+TempSeas
EvolTime_tc_30	5190.575	SR ~ TPI+ AET + PrepSeas+TempSeas+EvolTime_tc, EvolTime_tc ~ TPI + AET + PrepSeas+TempSeas
EvolTime_tc_29	5177.044	SR ~ AET + ClimVel+PrepSeas+TempSeas+EvolTime_tc, EvolTime_tc ~ AET + ClimVel+PrepSeas+TempSeas
EvolTime_tc_31	5167.294	SR ~ TPI+ AET + ClimVel+PrepSeas+TempSeas+EvolTime_tc, EvolTime_tc ~ TPI + AET + ClimVel+PrepSeas+TempSeas
DR_tc_5	3593.464	SR ~ PrepSeas+ DR_tc, DR_tc ~ PrepSeas
DR_tc_1	3556.764	SR ~ TPI+ DR_tc, DR_tc ~ TPI
DR_tc_3	3551.322	SR ~ ClimVel+ DR_tc, DR_tc ~ ClimVel
DR_tc_7	3458.439	SR ~ TPI+ ClimVel+DR_tc, DR_tc ~ TPI + ClimVel
DR_tc_9	3421.287	SR ~ TPI+ PrepSeas+DR_tc, DR_tc ~ TPI + PrepSeas
DR_tc_14	3416.026	SR ~ ClimVel+ DR_tc + PrepSeas, DR_tc ~ ClimVel+PrepSeas
DR_tc_4	3381.636	SR ~ TempSeas+ DR_tc, DR_tc ~ TempSeas
DR_tc_13	3336.002	SR ~ ClimVel+ DR_tc + TempSeas, DR_tc ~ ClimVel+TempSeas
DR_tc_20	3333.117	SR ~ TPI+ ClimVel+PrepSeas+DR_tc, DR_tc ~ TPI + ClimVel+PrepSeas
DR_tc_8	3307.162	SR ~ TPI+ TempSeas+DR_tc, DR_tc ~ TPI + TempSeas
DR_tc_19	3283.952	SR ~ TPI+ ClimVel+TempSeas+DR_tc, DR_tc ~ TPI + ClimVel+TempSeas
DR_tc_15	3247.102	SR ~ PrepSeas+ DR_tc + TempSeas, DR_tc ~ PrepSeas+TempSeas
DR_tc_25	3214.982	SR ~ ClimVel+TempSeas+PrepSeas+DR_tc, DR_tc ~ ClimVel+TempSeas+PrepSeas
DR_tc_21	3191.755	SR ~ TPI + TempSeas+PrepSeas+DR_tc, DR_tc ~ TPI + TempSeas+PrepSeas
DR_tc_28	3172.692	SR ~ TPI+ ClimVel+PrepSeas+TempSeas+DR_tc, DR_tc ~ TPI + ClimVel+PrepSeas+TempSeas
DR_tc_2	2612.035	SR ~ AET+ DR_tc, DR_tc ~ AET
DR_tc_6	2552.212	SR ~ TPI+ AET + DR_tc, DR_tc ~ TPI + AET
DR_tc_10	2470.393	SR ~ AET+ DR_tc + ClimVel, DR_tc ~ AET + ClimVel
DR_tc_12	2469.547	SR ~ AET+ DR_tc + PrepSeas, DR_tc ~ AET + PrepSeas
DR_tc_16	2458.702	SR ~ TPI+ AET + ClimVel+DR_tc, DR_tc ~ TPI + AET + ClimVel
DR_tc_18	2410.244	SR ~ TPI+ AET + PrepSeas+DR_tc, DR_tc ~ TPI + AET + PrepSeas
DR_tc_11	2400.77	SR ~ AET+ DR_tc + TempSeas, DR_tc ~ AET + TempSeas
DR_tc_17	2365.76	SR ~ TPI+ AET + TempSeas+DR_tc, DR_tc ~ TPI + AET + TempSeas
DR_tc_22	2341.3	SR ~ AET + ClimVel+TempSeas+DR_tc, DR_tc ~ AET + ClimVel+TempSeas
DR_tc_23	2334.304	SR ~ AET + ClimVel+PrepSeas+DR_tc, DR_tc ~ AET + ClimVel+PrepSeas
DR_tc_27	2329.515	SR ~ TPI+ AET + ClimVel+TempSeas+DR_tc, DR_tc ~ TPI + AET + ClimVel+TempSeas
DR_tc_26	2322.325	SR ~ TPI+ AET + ClimVel+PrepSeas+DR_tc, DR_tc ~ TPI + AET + ClimVel+PrepSeas
DR_tc_24	2283.237	SR ~ AET + TempSeas+PrepSeas+DR_tc, DR_tc ~ AET + TempSeas+PrepSeas
DR_tc_30	2248.133	SR ~ TPI+ AET + PrepSeas+TempSeas+DR_tc, DR_tc ~ TPI + AET + PrepSeas+TempSeas
DR_tc_29	2223.53	SR ~ AET + ClimVel+PrepSeas+TempSeas+DR_tc, DR_tc ~ AET + ClimVel+PrepSeas+TempSeas
DR_tc_31	2211.414	SR ~ TPI+ AET + ClimVel+PrepSeas+TempSeas+DR_tc, DR_tc ~ TPI + AET + ClimVel+PrepSeas+TempSeas

**TABLE A8:** List of the structural equation models we evaluated in this work, sorted by their values of the corrected Akaike Information Criteria (AICc). For this model selection procedure, we used the values of the DR and evolutionary time metrics obtained using the calibrated Bayesian timetree we inferred herein. Abbreviations as in Figure 3, except for *DR* = speciation rate (as measured with the DR metric).

	AICc	Equations
DR_ET_3	13,234.28	SR ~ ClimVel+ EvolTime + DR, EvolTime~ClimVel, DR ~ ClimVel
DR_ET_15	13,115.35	SR ~ PrepSeas+ EvolTime + DR + TempSeas, EvolTime~PrepSeas+TempSeas, DR ~ PrepSeas+TempSeas
DR_ET_9	13,033.3	SR ~ TPI+ PrepSeas+EvolTime + DR, EvolTime~TPI + PrepSeas, DR ~ TPI + PrepSeas
DR_ET_21	12,848.31	SR ~ TPI + TempSeas+PrepSeas+EvolTime + DR, EvolTime~TPI + TempSeas+PrepSeas, DR ~ TPI + TempSeas+PrepSeas
DR_ET_5	12,411.89	SR ~ PrepSeas+ EvolTime + DR, EvolTime~PrepSeas, DR ~ PrepSeas
DR_ET_8	12,329.21	SR ~ TPI+ TempSeas+EvolTime+DR, EvolTime~TPI + TempSeas, DR ~ TPI + TempSeas
DR_ET_14	12,201.5	SR ~ ClimVel+ EvolTime + DR + PrepSeas, EvolTime~ClimVel+PrepSeas, DR ~ ClimVel+PrepSeas
DR_ET_18	12,152.48	SR ~ TPI+ AET + PrepSeas+EvolTime + DR, EvolTime~TPI + AET + PrepSeas, DR ~ TPI + AET + PrepSeas
DR_ET_20	12,102.26	SR ~ TPI+ ClimVel+PrepSeas+EvolTime + DR, EvolTime~TPI + ClimVel+PrepSeas, DR ~ TPI + ClimVel+PrepSeas
DR_ET_12	12,082.66	SR ~ AET+ EvolTime + DR + PrepSeas, EvolTime~AET + PrepSeas, DR ~ AET + PrepSeas
DR_ET_6	12,043.99	SR ~ TPI+ AET + EvolTime + DR, EvolTime~TPI + AET, DR ~ TPI + AET
DR_ET_27	11,862.79	SR ~ TPI+ AET + ClimVel+TempSeas+EvolTime + DR, EvolTime~TPI + AET + ClimVel+TempSeas, DR ~ TPI + AET + ClimVel+TempSeas
DR_ET_24	11,749.89	SR ~ AET + TempSeas+PrepSeas+EvolTime + DR, EvolTime~AET + TempSeas+PrepSeas, DR ~ AET + TempSeas+PrepSeas
DR_ET_30	11,746.1	SR ~ TPI+ AET + PrepSeas+TempSeas+EvolTime + DR, EvolTime~TPI + AET + PrepSeas+TempSeas, DR ~ TPI + AET + PrepSeas+TempSeas
DR_ET_1	11,706.06	SR ~ TPI+ EvolTime + DR, EvolTime~TPI, DR ~ TPI
DR_ET_4	11,645.09	SR ~ TempSeas+ EvolTime + DR, EvolTime~TempSeas,DR ~ TempSeas
DR_ET_7	11,587.16	SR ~ TPI+ ClimVel+EvolTime + DR, EvolTime~TPI + ClimVel, DR ~ TPI + ClimVel
DR_ET_13	11,434.35	SR ~ ClimVel+ EvolTime + DR + TempSeas, EvolTime~ClimVel+TempSeas, DR ~ ClimVel+TempSeas
DR_ET_19	11,348.07	SR ~ TPI+ ClimVel+TempSeas+EvolTime + DR, EvolTime~TPI + ClimVel+TempSeas, DR ~ TPI + ClimVel+TempSeas
DR_ET_11	11,269.36	SR ~ AET+ EvolTime + DR + TempSeas, EvolTime~AET + TempSeas, DR ~ AET + TempSeas
DR_ET_2	11,240.14	SR ~ AET+ EvolTime + DR, EvolTime~AET, DR ~ AET
DR_ET_25	11,184.45	SR ~ ClimVel+TempSeas+PrepSeas+EvolTime + DR, EvolTime~ClimVel+TempSeas+PrepSeas, DR ~ ClimVel+TempSeas+PrepSeas
DR_ET_28	11,108.53	SR ~ TPI+ ClimVel+PrepSeas+TempSeas+EvolTime + DR, EvolTime~TPI + ClimVel+PrepSeas+TempSeas, DR ~ TPI + ClimVel+PrepSeas+TempSeas
DR_ET_17	11,044.71	SR ~ TPI+ AET + TempSeas+EvolTime + DR, EvolTime~TPI + AET + TempSeas, DR ~ TPI + AET + TempSeas
DR_ET_23	10,981.99	SR ~ AET + ClimVel+PrepSeas+EvolTime + DR, EvolTime~AET + ClimVel+PrepSeas, DR ~ AET + ClimVel+PrepSeas
DR_ET_26	10,964.05	SR ~ TPI+ AET + ClimVel+PrepSeas+EvolTime + DR, EvolTime~TPI + AET + ClimVel+PrepSeas, DR ~ TPI + AET + ClimVel+PrepSeas
DR_ET_29	10,813.71	SR ~ AET + ClimVel+PrepSeas+TempSeas+EvolTime + DR, EvolTime~AET + ClimVel+PrepSeas+TempSeas, DR ~ AET + ClimVel+PrepSeas+TempSeas
DR_ET_10	10,638.41	SR ~ AET+ EvolTime + DR + ClimVel, EvolTime~AET + ClimVel, DR ~ AET + ClimVel
DR_ET_16	10,423.3	SR ~ TPI+ AET + ClimVel+EvolTime + DR, EvolTime~TPI + AET + ClimVel, DR ~ TPI + AET + ClimVel
DR_ET_22	10,222.77	SR ~ AET + ClimVel+TempSeas+EvolTime + DR, EvolTime~AET + ClimVel+TempSeas, DR ~ AET + ClimVel+TempSeas
DR_ET_31	9927.269	SR ~ TPI+ AET + ClimVel+PrepSeas+TempSeas+EvolTime+DR, EvolTime~TPI + AET + ClimVel+PrepSeas+TempSeas, DR ~ TPI + AET + ClimVel+PrepSeas+TempSeas
EvolTime_5	6869.648	SR ~ PrepSeas+ EvolTime, EvolTime~PrepSeas
EvolTime_3	6834.235	SR ~ ClimVel+ EvolTime, EvolTime~ClimVel
EvolTime_1	6799.097	SR ~ TPI+ EvolTime, EvolTime~TPI
EvolTime_7	6738.326	SR ~ TPI+ ClimVel+EvolTime, EvolTime~TPI + ClimVel
EvolTime_14	6709.325	SR ~ ClimVel+ EvolTime+PrepSeas, EvolTime~ClimVel+PrepSeas
EvolTime_4	6694.051	SR ~ TempSeas+ EvolTime, EvolTime~TempSeas
EvolTime_9	6678.322	SR ~ TPI+ PrepSeas+EvolTime, EvolTime~TPI + PrepSeas
EvolTime_13	6658.689	SR ~ ClimVel+ EvolTime+TempSeas, EvolTime~ClimVel+TempSeas
EvolTime_20	6626.122	SR ~ TPI+ ClimVel+PrepSeas+EvolTime, EvolTime~TPI + ClimVel+PrepSeas
EvolTime_8	6604.689	SR ~ TPI+ TempSeas+EvolTime, EvolTime~TPI + TempSeas
EvolTime_19	6599.493	SR ~ TPI+ ClimVel+TempSeas+EvolTime, EvolTime~TPI + ClimVel+TempSeas
EvolTime_15	6574.683	SR ~ PrepSeas+ EvolTime+TempSeas, EvolTime~PrepSeas+TempSeas
EvolTime_25	6548.983	SR ~ ClimVel+TempSeas+PrepSeas+EvolTime, EvolTime~ClimVel+TempSeas+PrepSeas
EvolTime_21	6505.965	SR ~ TPI + TempSeas+PrepSeas+EvolTime, EvolTime~TPI + TempSeas+PrepSeas
EvolTime_28	6502.555	SR ~ TPI+ ClimVel+PrepSeas+TempSeas+EvolTime, EvolTime~TPI + ClimVel+PrepSeas+TempSeas
DR_3	6399.639	SR ~ ClimVel+ DR, DR ~ ClimVel
DR_1	6398.111	SR ~ TPI+ DR, DR ~ TPI
DR_5	6378.334	SR ~ PrepSeas+ DR, DR ~ PrepSeas
DR_7	6298.883	SR ~ TPI+ ClimVel+DR, DR ~ TPI + ClimVel
DR_14	6189.847	SR ~ ClimVel+ DR + PrepSeas, DR ~ ClimVel+PrepSeas
DR_9	6186.844	SR ~ TPI+ PrepSeas+DR, DR ~ TPI + PrepSeas
DR_4	6112.11	SR ~ TempSeas+ DR, DR ~ TempSeas
DR_20	6098.9	SR ~ TPI+ ClimVel+PrepSeas+DR, DR ~ TPI + ClimVel+PrepSeas
DR_13	6079.227	SR ~ ClimVel+ DR + TempSeas, DR ~ ClimVel+TempSeas
DR_8	6024.39	SR ~ TPI+ TempSeas+DR, DR ~ TPI + TempSeas
DR_19	6018.169	SR ~ TPI+ ClimVel+TempSeas+DR, DR ~ TPI + ClimVel+TempSeas
DR_15	5925.309	SR ~ PrepSeas+ DR + TempSeas, DR ~ PrepSeas+TempSeas
DR_25	5901.665	SR ~ ClimVel+TempSeas+PrepSeas+DR, DR ~ ClimVel+TempSeas+PrepSeas
DR_21	5853.286	SR ~ TPI + TempSeas+PrepSeas+DR, DR ~ TPI + TempSeas+PrepSeas
DR_28	5849.843	SR ~ TPI+ ClimVel+PrepSeas+TempSeas+DR, DR ~ TPI + ClimVel+PrepSeas+TempSeas
EvolTime_2	5762.814	SR ~ AET+ EvolTime, EvolTime~AET
EvolTime_6	5696.741	SR ~ TPI+ AET + EvolTime, EvolTime~TPI + AET
EvolTime_12	5675.796	SR ~ AET+ EvolTime+PrepSeas, EvolTime~AET + PrepSeas
EvolTime_10	5650.862	SR ~ AET+ EvolTime+ClimVel, EvolTime~AET + ClimVel
EvolTime_11	5648.063	SR ~ AET+ EvolTime+TempSeas, EvolTime~AET + TempSeas
EvolTime_16	5639.26	SR ~ TPI+ AET + ClimVel+EvolTime, EvolTime~TPI + AET + ClimVel
EvolTime_18	5609.317	SR ~ TPI+ AET + PrepSeas+EvolTime, EvolTime~TPI + AET + PrepSeas
EvolTime_17	5604.966	SR ~ TPI+ AET + TempSeas+EvolTime, EvolTime~TPI + AET + TempSeas
EvolTime_22	5592.164	SR ~ AET + ClimVel+TempSeas+EvolTime, EvolTime~AET + ClimVel+TempSeas
EvolTime_27	5580.309	SR ~ TPI+ AET + ClimVel+TempSeas+EvolTime, EvolTime~TPI + AET + ClimVel+TempSeas
EvolTime_23	5564.801	SR ~ AET + ClimVel+PrepSeas+EvolTime, EvolTime~AET + ClimVel+PrepSeas
EvolTime_24	5556.511	SR ~ AET + TempSeas+PrepSeas+EvolTime, EvolTime~AET + TempSeas+PrepSeas
EvolTime_26	5553.113	SR ~ TPI+ AET + ClimVel+PrepSeas+EvolTime, EvolTime~TPI + AET + ClimVel+PrepSeas
EvolTime_30	5513.272	SR ~ TPI+ AET + PrepSeas+TempSeas+EvolTime, EvolTime~TPI + AET + PrepSeas+TempSeas
EvolTime_29	5500.593	SR ~ AET + ClimVel+PrepSeas+TempSeas+EvolTime, EvolTime~AET + ClimVel+PrepSeas+TempSeas
EvolTime_31	5488.587	SR ~ TPI+ AET + ClimVel+PrepSeas+TempSeas+EvolTime, EvolTime~TPI + AET + ClimVel+PrepSeas+TempSeas
DR_2	5318.972	SR ~ AET+ DR, DR ~ AET
DR_6	5250.328	SR ~ TPI+ AET + DR, DR ~ TPI + AET
DR_10	5168.47	SR ~ AET+ DR + ClimVel, DR ~ AET + ClimVel
DR_16	5155.665	SR ~ TPI+ AET + ClimVel+DR, DR ~ TPI + AET + ClimVel
DR_12	5048.427	SR ~ AET+ DR + PrepSeas, DR ~ AET + PrepSeas
DR_11	5042.895	SR ~ AET+ DR + TempSeas, DR ~ AET + TempSeas
DR_17	5001.467	SR ~ TPI+ AET + TempSeas+DR, DR ~ TPI + AET + TempSeas
DR_22	4983.583	SR ~ AET + ClimVel+TempSeas+DR, DR ~ AET + ClimVel+TempSeas
DR_18	4980.552	SR ~ TPI+ AET + PrepSeas+DR, DR ~ TPI + AET + PrepSeas
DR_27	4970.713	SR ~ TPI+ AET + ClimVel+TempSeas+DR, DR ~ TPI + AET + ClimVel+TempSeas
DR_23	4905.603	SR ~ AET + ClimVel+PrepSeas+DR, DR ~ AET + ClimVel+PrepSeas
DR_26	4892.41	SR ~ TPI+ AET + ClimVel+PrepSeas+DR, DR ~ TPI + AET + ClimVel+PrepSeas
DR_24	4818.157	SR ~ AET + TempSeas+PrepSeas+DR, DR ~ AET + TempSeas+PrepSeas
DR_30	4776.585	SR ~ TPI+ AET + PrepSeas+TempSeas+DR, DR ~ TPI + AET + PrepSeas+TempSeas
DR_29	4758.615	SR ~ AET + ClimVel+PrepSeas+TempSeas+DR, DR ~ AET + ClimVel+PrepSeas+TempSeas
DR_31	4745.312	SR ~ TPI+ AET + ClimVel+PrepSeas+TempSeas+DR, DR ~ TPI + AET + ClimVel+PrepSeas+TempSeas

**TABLE A9:** Coefficient estimates of the best‐fitted structural equation model of the ecological and evolutionary factors driving species richness patterns in the Dipsadidae. For this model, we used the values of the DR and evolutionary time metrics obtained using the calibrated Bayesian timetree we inferred herein after including taxa lacking genetic data with the software TACT and the range maps compiled by Roll et al. (2017). Abbreviations as in Figure 3.

Response variable	Predictor variable	Estimate	*p*	Standardized estimate
SR	TPI	−0.029	0.000	−0.023
SR	AET	0.461	0.000	0.360
SR	ClimVel	0.028	0.002	0.022
SR	PrepSeas	0.051	0.000	0.039
SR	TempSeas	0.232	0.000	0.181
SR	DR_tc	0.125	0.000	0.098
DR_tc	TPI	0.009	0.334	0.009
DR_tc	AET	−0.115	0.000	−0.115
DR_tc	ClimVel	0.063	0.000	0.063
DR_tc	PrepSeas	−0.108	0.000	−0.108
DR_tc	TempSeas	0.312	0.000	0.312

**TABLE A10:** Coefficient estimates of the best‐fitted structural equation model of the ecological and evolutionary factors driving species richness patterns in the Dipsadidae. For this model, we used the values of the DR and evolutionary time metrics obtained using the calibrated Bayesian timetree we inferred herein and the range maps compiled by Roll et al. (2017). Abbreviations as in Figure 3, except for *DR* = speciation rate (as measured with the DR metric).

Response variable	Predictor variable	Estimate	*p*	Standardized estimate
SR	TPI	−0.029	0.0001	−0.0236
SR	AET	0.4618	0	0.3755
SR	ClimVel	0.0382	0	0.031
SR	PrepSeas	0.0394	0	0.0321
SR	TempSeas	0.2589	0	0.2105
SR	DR	−0.0141	0.1164	−0.0114
DR	TPI	0.0194	0.2067	0.0194
DR	AET	−0.3765	0	−0.3765
DR	ClimVel	0.0739	0.0001	0.0739
DR	PrepSeas	−0.2684	0	−0.2684
DR	TempSeas	0.5826	0	0.5826
