# Supplementary material for: Regional Climate Drives Spatial Variation in Species Richness in the Most Diverse Family of Neotropical Snakes (Colubroidea: Dipsadidae)
Source: Ecol Evol. 2025 Jul 7;15(7):e71716. doi: 10.1002/ece3.71716 (PMC12230370; doi:10.1002/ece3.71716)
Supplement: Supplementary file 1 — Table S1. Accession numbers of the sequences used for constructing the timetree employed for this work. Table S2. Taxonomic arrangement of the Dipsadidae used for the TACT analyses performed for this work, following Uetz et al. (2024); References used for constructing Table S2. [file ECE3-15-e71716-s001.docx]

Supplementary Materials for **Regional speciation drives spatial variation in species richness in the most diverse family of Neotropical snakes (Colubroidea: Dipsadidae)**

**Supplementary Tables:**

**Supplementary Table S1.** Accession numbers of each of the sequences used for constructing the time tree presented in Suppl. Figure 1.

**Supplementary Table S2.** Taxonomic arrangement of the Dipsadidae used for the TACT analyses performed for this work, following Uetz et al. (2023).

**References used for constructing Table S2**

**Supplementary Tables**

**Table S1.** Accession numbers of each of the sequences used for constructing the time tree presented in Suppl. Figure 2.1.

| **Species** | **Number of loci** | **12S** | **16S** | **cytb** | **bdnf** | **cmos** | **nt3** | **nd4** | **nd2** | **RAG2** |
| --- | --- | --- | --- | --- | --- | --- | --- | --- | --- | --- |
| *Acrochordus_granulatus* | 9 | AB177879 | AB177879 | AB177879 | FJ433981 | HM234057 | FJ434082 | AB177879.1 | AB177879.1 | EF144093.1 |
| *Atractaspis_micropholis* | 8 | AF544740 | AY611823 | AY612006 | FJ433994 | AF544677 | FJ434095 | FJ404336 | - | AY612006.1 |
| *Boa_constrictor* | 9 | NC007398 | NC007398 | NC007398 | AY988030 | AF544676 | AY988047 | NC007398 | NC007398 | FJ433905.1 |
| *Calamaria_pavimentata* | 8 | KX694584 | KX694624 | KX694890 | JQ599014 | KX694804 | KX694999 | - | MK199069 | EF144116.1 |
| *Coluber_constrictor* | 8 | U96794_AY122819_YPX528 | YPX528 | EU180486 | JQ599015 | YPX528 | YPX528 | AY487040 | AY487001 | - |
| *Lampropeltis_getula* | 8 | KX694603 | KX694649 | FJ997820 | EU402645 | KX694811 | KX695024 | MG672616 | MG672874.1 | - |
| *Adelphicos_quadrivirgatus* | 4 | - | - | GQ895853 | - | GQ895796 | MH823662 | JX398446 | - | - |
| *Alsophis_antillensis* | 7 | FJ416691 | FJ416702 | FJ416726 | JQ599005 | - | - | FJ416800 | FJ416764 | FJ416837 |
| *Alsophis_manselli* | 6 | AF158528 | AF158528 | FJ416727 | - | - | - | FJ416801 | FJ416765 | FJ416838 |
| *Alsophis_rijgersmaei* | 6 | FJ416697 | FJ416708 | FJ416729 | - | - | - | FJ416803 | FJ416767 | FJ416840 |
| *Alsophis_rufiventris* | 6 | FJ416698 | FJ416709 | FJ416730 | - | - | - | FJ416804 | FJ416768 | FJ416841 |
| *Alsophis_sajdaki* | 6 | AF158455 | AF158524 | FJ416731 | - | - | - | FJ416805 | FJ416769 | FJ416842 |
| *Alsophis_sibonius* | 6 | FJ416692 | FJ416703 | FJ416728 | - | - | - | FJ416802 | FJ416766 | FJ416839 |
| *Amastridium_sapperi* | 4 | - | - | GQ334479 | - | GQ895797 | GQ334663 | GQ334580 | - | - |
| *Amastridium_veliferum* | 1 | - | MH140470 | - | - | - | - | - | - | - |
| *Apostolepis_albicollaris* | 6 | JQ598793 | JQ598856 | CBGM00201 | CBGM00201 | JQ598965 | CBGM00201 | - | - | - |
| *Apostolepis_assimilis* | 4 | GQ457781 | GQ457724 | - | JQ599007 | GQ457843 | - | - | - | - |
| *Apostolepis_cearensis* | 4 | JQ598794 | JQ598857 | - | CTMZ00097 | JQ598966 | - | - | - | - |
| *Apostolepis_dimidiata* | 6 | GQ457782 | GQ457725 | JQ598917 | JQ599008 | GQ457844 | CBGM00042 | - | - | - |
| *Apostolepis_flavotorquata* | 4 | JQ598795 | JQ598858 | GQ895854 | - | JQ598967 | - | - | - | - |
| *Apostolepis_rondoni* | 2 | JQ598796 | - | - | - | JQ598968 | - | - | - | - |
| *Apostolepis_sanctaeritae* | 3 | JQ598797 | JQ598859 | - | - | JQ598969 | - | - | - | - |
| *Arcanumophis_problematicus* | 3 | MH513952 | - | MH536833 | MH536834 | - | - | - | - | - |
| *Arrhyton_dolichura* | 6 | AF158438 | AF158507 | FJ416721 | - | - | - | FJ416795 | FJ416759.1 | FJ416832.1 |
| *Arrhyton_procerum* | 6 | AF158452 | AF158521 | FJ416723 | - | - | - | FJ416797 | FJ416761.1 | FJ416834.1 |
| *Arrhyton_redimitum* | 6 | AF158439 | AF158508 | FJ416720 | - | - | - | FJ416794 | FJ416758.1 | FJ416831.1 |
| *Arrhyton_supernum* | 6 | AF158436 | AF158505 | FJ416718 | - | - | - | FJ416792 | FJ416756.1 | FJ416829.1 |
| *Arrhyton_taeniatum* | 6 | AF158453 | AF158522 | FJ416717 | - | - | - | FJ416791 | FJ416755.1 | FJ416828.1 |
| *Arrhyton_tanyplectum* | 6 | AF158446 | AF158516 | FJ416722 | - | - | - | FJ416796 | FJ416760.1 | FJ416833.1 |
| *Arrhyton_vittatum* | 6 | AF158437 | AF158506 | FJ416719 | - | - | - | FJ416793 | FJ416757.1 | FJ416830.1 |
| *Atractus_albuquerquei* | 6 | GQ457783 | GQ457726 | JQ598918 | JQ599009 | GQ457845 | CBGM00072 | - | - | - |
| *Atractus_atlas* | 1 | - | MH790470 | - | - | - | - | - | - | - |
| *Atractus_badius* | 2 | AF158425 | KX277233 | - | - | - | - | - | - | - |
| *Atractus_boimirim* | 1 | - | MH790478 | - | - | - | - | - | - | - |
| *Atractus_carrioni* | 2 | - | KY610046 | - | - | - | - | KY610094 | - | - |
| *Atractus_cerberus* | 3 | - | KY610047 | KY610073 | - | - | - | KY610095 | - | - |
| *Atractus_dapsilis* | 2 | MH790542 | MH790481 | - | - | - | - | - | - | - |
| *Atractus_duboisi* | 2 | - | KT944041 | - | - | - | - | KT944059 | - | - |
| *Atractus_dunni* | 3 | - | KY610048 | KT944050 | - | - | - | KY610096.1 | - | - |
| *Atractus_ecuadorensis* | 1 | - | - | - | - | - | - | KY610100 | - | - |
| *Atractus_elaps* | 4 | - | KY610052 | KY610076 | - | - | GU353273 | EF078584 | - | - |
| *Atractus_esepe* | 2 | - | KY610053 | - | - | - | - | KY610102 | - | - |
| *Atractus_favae* | 2 | MH790543 | MH790482 | - | - | - | - | - | - | - |
| *Atractus_flammigerus* | 2 | AF158402 | KR815897 | - | - | - | - | - | - | - |
| *Atractus_gigas* | 3 | - | KT944043 | KT944053 | - | - | - | KT944061 | - | - |
| *Atractus_imperfectus* | 1 | - | MH140626 | - | - | - | - | - | - | - |
| *Atractus_iridescens* | 3 | - | KT944039 | KT944052 | - | - | - | KT944058.1 | - | - |
| *Atractus_lasallei* | 5 | MHUAT0754 | MHUAT0754 | - | MHUAT0754 | MHUAT0754 | MHUAT0754 | - | - | - |
| *Atractus_latifrons* | 2 | MH790544 | MH790495 | - | - | - | - | - | - | - |
| *Atractus_lehmanni* | 2 | - | KY610058 | - | - | - | - | KY610103 | - | - |
| *Atractus_major* | 3 | MH790545 | KT944045 | - | - | - | - | KY610105.1 | - | - |
| *Atractus_microrhynchus* | 3 | - | KY610060 | KY610083 | - | - | - | KY610106 | - | - |
| *Atractus_modestus* | 3 | - | KY610061 | KY610084 | - | - | - | KY610107 | - | - |
| *Atractus_multicinctus* | 3 | - | KY610062 | KY610085 | - | - | - | KY610108 | - | - |
| *Atractus_occidentalis* | 3 | - | KY610065 | KY610086 | - | - | - | KY610109.1 | - | - |
| *Atractus_paucidens* | 2 | - | KY610067 | - | - | - | - | KY610114.1 | - | - |
| *Atractus_pyroni* | 3 | - | KY610068 | KY610089 | - | - | - | KY610115 | - | - |
| *Atractus_resplendens* | 4 | KT944036 | KT944042 | KT944055 | - | - | - | KT944060 | - | - |
| *Atractus_reticulatus* | 3 | JQ598798 | - | - | CBGM00091 | JQ598970 | - | - | - | - |
| *Atractus_riveroi* | 1 | - | MH790526 | - | - | - | - | - | - | - |
| *Atractus_roulei* | 3 | - | KY610069 | KY610091 | - | - | - | KY610117.1 | - | - |
| *Atractus_savagei* | 3 | - | KY610070 | KY610092 | - | - | - | KY610118 | - | - |
| *Atractus_schach* | 4 | JQ598799 | JQ598860 | - | - | JQ598971 | CBGM00034 | - | - | - |
| *Atractus_tartarus* | 1 | - | KX926018 | - | - | - | - | - | - | - |
| *Atractus_torquatus* | 2 | MH790547 | MH790535 | - | - | - | - | - | - | - |
| *Atractus_touzeti* | 3 | - | KY610071 | KY610093 | - | - | - | KY610119 | - | - |
| *Atractus_trefauti* | 1 | - | MH790536 | - | - | - | - | - | - | - |
| *Atractus_trihedrurus* | 6 | GQ457784 | GQ457727 | JQ598919 | JQ599010 | GQ457846 | CBGM00028 | - | - | - |
| *Atractus_trilineatus* | 6 | MK648011 | MK648019 | MK648028 | - | MK648044 | JX398731 | MK648036 | - | - |
| *Atractus_typhon* | 3 | - | KT944044 | KT944054 | - | - | - | KT944062 | - | - |
| *Atractus_wagleri* | 5 | MH790548 | MH790541 | GQ334480 | - | - | GQ334664 | GQ334581 | - | - |
| *Atractus_zebrinus* | 5 | JQ598800 | JQ598861 | - | CBGM00060 | JQ598972 | CBGM00060 | - | - | - |
| *Atractus_zidocki* | 2 | AF158426 | AF158487 | - | - | - | - | - | - | - |
| *Boiruna_maculata* | 6 | GQ457785 | JQ598862 | JQ598920 | JQ599011 | GQ457847 | YPX113 | - | - | - |
| *Borikenophis_portoricensis* | 9 | FJ416696 | FJ416707 | FJ416732 | JQ599012 | AF471126 | CTMZ-04118 | U49308 | FJ416770.1 | FJ416843.1 |
| *Borikenophis_prymnus* | 5 | AF158448 | - | FJ416733 | - | - | - | FJ416807 | FJ416771 | FJ416844 |
| *Borikenophis_variegatus* | 6 | FJ416700 | FJ416711 | FJ416734 | - | - | - | FJ416808 | FJ416772.1 | FJ416845.1 |
| *Caaeteboia_amarali* | 4 | GQ457807 | GQ457747 | JQ598921 | - | GQ457867 | - | - | - | - |
| *Calamodontophis_paucidens* | 3 | GQ457786 | GQ457728 | - | - | GQ457848 | - | - | - | - |
| *Caraiba_andreae* | 6 | AF158442 | AF158511 | FJ416743 | - | - | - | FJ416817 | FJ416781 | FJ416854 |
| *Carphophis_amoenus* | 4 | AY577013 | AY577022 | AF471067 | - | DQ112082 | - | - | - | - |
| *Carphophis_vermis* | 4 | - | - | KP765656 | - | KP765638 | JX398729 | JX398449 | - | - |
| *Chersodromus_liebmanni* | 3 | - | - | JX398604 | - | - | JX398732 | JX398451 | - | - |
| *Clelia_clelia* | 4 | AF158403 | AF158472 | GQ895859 | - | JQ598973 | - | - | - | - |
| *Coniophanes_fissidens* | 3 | - | - | EF078538 | - | - | GU353274 | EF078586 | - | - |
| *Coniophanes_imperialis* | 1 | - | - | MT308778 | - | - | - | - | - | - |
| *Conophis_lineatus* | 6 | GQ457788 | JQ598865 | JQ598924 | JQ599016 | JQ598975 | JX398739 | - | - | - |
| *Conophis_vittatus* | 2 | - | - | GQ895862 | - | GQ895806 | - | - | - | - |
| *Contia_longicaudae* | 3 | - | - | GU112403 | GU112371 | - | - | GU112427 | - | - |
| *Contia_tenuis* | 6 | AY577021 | AY577030 | AF471095 | GU112361 | AF471134 | - | AF402658 | - | - |
| *Crisantophis_nevermanni* | 2 | GU018152 | GU018169 | - | - | - | - | - | - | - |
| *Cryophis_hallbergi* | 4 | - | - | EF078496 | - | GQ895807 | GQ334666 | EF078544 | - | - |
| *Cubophis_cantherigerus* | 9 | AF158405 | AF158475 | KF612921 | FJ433999 | AF544694 | FJ434100 | FJ416818 | FJ416782 | EF144109 |
| *Cubophis_caymanus* | 6 | FJ416693 | FJ416704 | FJ416745 | - | - | - | FJ416820 | FJ416784 | FJ416856 |
| *Cubophis_fuscicauda* | 5 | FJ416695 | FJ416706 | FJ416747 | - | - | - | FJ416822 | FJ416786 | - |
| *Cubophis_ruttyi* | 5 | FJ416699 | FJ416710 | FJ416746 | - | - | - | FJ416821 | FJ416785 | - |
| *Cubophis_vudii* | 7 | AF158443 | AF158512 | FJ416744 | - | JQ598976 | - | FJ416819 | FJ416783 | FJ416855 |
| *Diadophis_punctatus* | 8 | AF544765 | KX694636 | EU193843 | JQ599017 | AF471122 | KX695009 | EU193987 | - | EF144110 |
| *Diaphorolepis_wagneri* | 4 | - | KR814752 | KT345361 | - | KR814764 | - | KR814778.1 | - | - |
| *Dipsas_albifrons* | 5 | JQ598803 | JQ598866 | JQ598925 | JQ599019 | - | YPX117 | - | - | - |
| *Dipsas_andiana* | 4 | - | MH341014.1 | MH375012.1 | - | - | JX398744 | JX398453 | - | - |
| *Dipsas_articulata* | 5 | JQ598804 | JQ598867 | - | JQ599020 | - | JX398740 | JX398454 | - | - |
| *Dipsas_bicolor* | 2 | - | - | - | - | - | JX398741 | JX398455 | - | - |
| *Dipsas_bobridgelyi* | 2 | - | MH341016.1 | MH374984.1 | - | - | - | - | - | - |
| *Dipsas_bucephala* | 5 | MH341087.1 | MH341018.1 | MH375026.1 | - | MH374932.1 | - | MH375052.1 | - | - |
| *Dipsas_catesbyi* | 7 | JQ598805 | JQ598868 | JQ598926 | JQ599021 | JQ598977 | KX695010 | EF078585 | - | - |
| *Dipsas_elegans* | 2 | - | MH341021.1 | MH375033.1 | - | - | - | - | - | - |
| *Dipsas_ellipsifera* | 3 | - | MH341024.1 | MH375030.1 | - | MH374934.1 | - | - | - | - |
| *Dipsas_gaigeae* | 3 | - | - | JX398613 | - | - | JX398735 | JX398462 | - | - |
| *Dipsas_georgejetti* | 3 | - | MH341025.1 | MH375024.1 | - | MH374936.1 | - | - | - | - |
| *Dipsas_gracilis* | 5 | - | MH341028.1 | MH374980.1 | - | MH374938.1 | JX398747 | JX398466 | - | - |
| *Dipsas_indica* | 7 | GQ457789 | GQ457730 | JX398618 | CBGM00027 | GQ457850 | JX398734 | JX398468 | - | - |
| *Dipsas_jamespetersi* | 5 | - | MH341041.1 | MH375028.1 | - | MH374942.1 | JX398818 | JX398555 | - | - |
| *Dipsas_klebbai* | 3 | - | MH341043.1 | MH375022.1 | - | MH374945.1 | - | - | - | - |
| *Dipsas_neivai* | 5 | GQ457790 | GQ457731 | - | CBGM00100 | GQ457851 | CBGM00100 | - | - | - |
| *Dipsas_nicholsi* | 2 | - | - | JX398619 | - | - | - | JX398469 | - | - |
| *Dipsas_oligozonata* | 4 | - | MH341050.1 | MH375029.1 | - | - | JX398817 | JX398554 | - | - |
| *Dipsas_oreas* | 3 | - | MH341053.1 | MH374987.1 | - | - | - | MH375038.1 | - | - |
| *Dipsas_oswaldobaezi* | 2 | - | MH341060.1 | MH374997.1 | - | - | - | - | - | - |
| *Dipsas_palmeri* | 5 | MH341092.1 | MH341065.1 | MH375009.1 | - | MH374949.1 | - | MH375046.1 | - | - |
| *Dipsas_pavonina* | 5 | - | KX660268 | KX660537 | - | MH374952 | KX652078 | JX398470 | - | - |
| *Dipsas_peruana* | 5 | - | KX660269 | KX660538 | - | KX660406 | KX652080 | JX398472 | - | - |
| *Dipsas_pratti* | 3 | - | - | GQ334482 | - | - | GQ334667 | GQ334583 | - | - |
| *Dipsas_temporalis* | 4 | - | MH341069.1 | MH375003.1 | - | - | JX398753 | JX398478 | - | - |
| *Dipsas_variegata* | 5 | AF158406 | AF158476 | JX398631 | - | - | JX398737 | JX398481 | - | - |
| *Dipsas_vermiculata* | 6 | MH341095.1 | MH341071.1 | MH374972.1 | - | MH374953.1 | JX398754 | MH375049.1 | - | - |
| *Dipsas_williamsi* | 2 | - | - | MH374968.1 | - | - | - | MH375041.1 | - | - |
| *Drepanoides_anomalus* | 6 | GQ457791 | GQ457732 | JQ598927 | CBGM00038 | GQ457852 | CBGM00038 | - | - | - |
| *Echinanthera_melanostigma* | 4 | JQ598806 | JQ598869 | JQ598928 | - | - | YPX120 | - | - | - |
| *Echinanthera_undulata* | 5 | JQ598807 | JQ598870 | JQ598929 | JQ599022 | JQ598978 | - | - | - | - |
| *Elapomorphus_quinquelineatusB* | 6 | GQ457794 | GQ457735 | JQ598930 | JQ599023 | GQ457855 | CTMZ13858 | - | - | - |
| *Enuliophis_sclateri* | 4 | - | MH140724 | JX398635 | - | - | JX398757 | JX398485 | - | - |
| *Enulius_flavitorques* | 4 | - | MH140725 | JX398636 | - | - | JX398758 | JX398486 | - | - |
| *Erythrolamprus_aesculapii* | 7 | GQ457795 | GQ457736 | GQ895871 | JQ599024 | GQ457856 | MN703645 | MN703551 | - | - |
| *Erythrolamprus_almadensis* | 5 | JQ598808 | JQ598871 | - | CBGM00180 | JQ598979 | CBGM00180 | - | - | - |
| *Erythrolamprus_atraventer* | 5 | JQ598809 | JQ598872 | - | CBGM00048 | JQ598980 | CBGM00048 | - | - | - |
| *Erythrolamprus_bizona* | 3 | - | KY986513.1 | - | KY986502.1 | KY986493.1 | - | - | - | - |
| *Erythrolamprus_breviceps* | 2 | AF158464 | AF158533 | - | - | - | - | - | - | - |
| *Erythrolamprus_ceii* | 5 | JQ598810 | JQ598873 | - | CBGM00054 | JQ598981 | CBGM00054 | - | - | - |
| *Erythrolamprus_cobella* | 3 | - | KY986514.1 | - | KY986503.1 | KY986489.1 | - | - | - | - |
| *Erythrolamprus_cursor* | 2 | JX905307 | JX905311 | - | - | - | - | - | - | - |
| *Erythrolamprus_epinephelus* | 5 | MHUAT0694 | MHUAT0694 | - | MHUAT0694 | MHUAT0694 | MHUAT0694 | - | - | - |
| *Erythrolamprus_festae* | 5 | MN703457 | MN703486 | MN703531 | - | - | MN703647 | MN703568 | - | - |
| *Erythrolamprus_jaegeri* | 5 | GQ457809 | GQ457749 | - | CBGM00177 | GQ457869 | CBGM00177 | - | - | - |
| *Erythrolamprus_juliae* | 2 | AF158445 | AF158514 | - | - | - | - | - | - | - |
| *Erythrolamprus_melanotus* | 5 | MHUAT1249 | MHUAT1249 | - | MHUAT1249 | MHUAT1249 | MHUAT1249 | - | - | - |
| *Erythrolamprus_miliaris* | 6 | JQ598811 | JQ598874 | JQ598931 | JQ599025 | JQ598982 | KX695016 | - | - | - |
| *Erythrolamprus_mimus* | 7 | MN703446 | MN703479 | MN703522 | KY986499 | KY986496 | MN703636 | MN703544 | - | - |
| *Erythrolamprus_ocellatus* | 2 | - | KY986518.1 | - | - | KY986490.1 | - | - | - | - |
| *Erythrolamprus_poecilogyrus* | 5 | JQ598812 | KY986516 | - | KY986500 | KY986491 | CTMZ00015 | - | - | - |
| *Erythrolamprus_pseudoreginae* | 3 | MK287470 | MK287477 | - | - | MK287484 | - | - | - | - |
| *Erythrolamprus_pygmaeus* | 5 | MN703453 | MN703482 | MN703528 | - | - | MN703643 | MN703550 | - | - |
| *Erythrolamprus_reginae* | 7 | JQ598813 | JQ598876 | MN703521 | CBGM00192 | JQ598983 | CBGM00192 | MN703543 | - | - |
| *Erythrolamprus_sagittifer* | 1 | - | MN276240 | - | - | - | - | - | - | - |
| *Erythrolamprus_taeniogaster* | 4 | MN703425 | MN703464 | MN703504 | - | - | MN703615 | - | - | - |
| *Erythrolamprus_typhlus* | 5 | GQ457811 | GQ457751 | - | CBGM00195 | GQ457871 | CBGM00195 | - | - | - |
| *Erythrolamprus_vitti* | 5 | MN703431 | MN703498 | MN703509 | - | - | MN703621 | MN703561 | - | - |
| *Erythrolamprus_zweifeli* | 3 | MK287475 | MK287482 | - | - | MK287488 | - | - | - | - |
| *Farancia_abacura* | 6 | KR814628 | KR814639 | U69832 | - | KR814674 | - | DQ902307 | DQ902239 | - |
| *Farancia_erythrogramma_merged* | 6 | CTMZ04131 | YPX122 | KP765663 | YPX122 | KP765647 | YPX122 | - | - | - |
| *Geophis_anocularis* | 1 | - | - | - | - | - | MH823655 | - | - | - |
| *Geophis_bicolor* | 3 | - | - | JX398637 | - | - | JX398759 | JX398487 | - | - |
| *Geophis_blanchardi* | 1 | - | - | - | - | - | MH823615 | - | - | - |
| *Geophis_brachycephalus* | 2 | - | MH140742 | - | - | - | MH823616 | - | - | - |
| *Geophis_cancellatus* | 1 | - | - | - | - | - | MH823619 | - | - | - |
| *Geophis_carinosus* | 2 | - | - | GQ895872 | - | GQ895815 | - | - | - | - |
| *Geophis_damiani* | 1 | - | - | - | - | - | MH823621 | - | - | - |
| *Geophis_duellmani* | 1 | - | - | - | - | - | MH823623 | - | - | - |
| *Geophis_dubius* | 2 | - | - | KC917316 | - | - | MH823622 | - | - | - |
| *Geophis_dugesii* | 1 | - | - | - | - | - | MH823612 | - | - | - |
| *Geophis_godmani* | 5 | JQ598814 | JQ598877 | JQ598932 | JQ599026 | - | MH823625 | - | - | - |
| *Geophis_fulvoguttatus* | 1 | - | - | - | - | - | MH823630 | - | - | - |
| *Geophis_hoffmanni* | 1 | - | - | - | - | - | MH823652 | - | - | - |
| *Geophis_immaculatus* | 1 | - | - | - | - | - | MH823635 | - | - | - |
| *Geophis_juarezi* | 2 | - | - | KC917315 | - | - | MH823624 | - | - | - |
| *Geophis_laticinctus* | 1 | - | - | - | - | - | MH823631 | - | - | - |
| *Geophis_latifrontalis* | 2 | - | - | KC917322 | - | - | MH823627 | - | - | - |
| *Geophis_lorancai* | 1 | - | - | - | - | - | MH823654 | - | - | - |
| *Geophis_multitorques* | 1 | - | - | - | - | - | MH823628 | - | - | - |
| *Geophis_nasalis* | 1 | - | - | - | - | - | MH823637 | - | - | - |
| *Geophis_nephodrymus* | 1 | - | - | - | - | - | MH823657 | - | - | - |
| *Geophis_nigrocinctus* | 3 | - | - | JX398638 | - | - | MH823658 | JX398488 | - | - |
| *Geophis_occabus* | 2 | - | - | KC917323 | - | - | MH823633 | - | - | - |
| *Geophis_omiltemanus* | 2 | - | - | JX398639 | - | - | MH823648 | - | - | - |
| *Geophis_petersii* | 1 | - | - | - | - | - | MH823642 | - | - | - |
| *Geophis_rhodogaster* | 1 | - | - | - | - | - | MH823640 | - | - | - |
| *Geophis_russatus* | 1 | - | - | - | - | - | MH823632 | - | - | - |
| *Geophis_sallaei* | 1 | - | - | - | - | - | MH823629 | - | - | - |
| *Geophis_semidoliatus* | 1 | - | - | - | - | - | MH823644 | - | - | - |
| *Geophis_tarascae* | 3 | - | - | JX398640 | - | - | JX398761 | JX398489 | - | - |
| *Geophis_tectus* | 2 | - | MH140744 | - | - | - | MH823649 | - | - | - |
| *Geophis_turbidus* | 2 | - | - | KC917321 | - | - | MH823647 | - | - | - |
| *Geophis_zeledoni* | 1 | - | - | - | - | - | MH823656 | - | - | - |
| *Gomesophis_brasiliensis* | 2 | GQ457796 | GQ457737 | - | - | - | - | - | - | - |
| *Haitiophis_anomalus* | 2 | FJ666091 | FJ666092 | - | - | - | - | - | - | - |
| *Helicops_angulatus* | 8 | GQ457797 | GQ457738 | AF471037 | JQ599027 | GQ457857 | MN032485 | - | FJ416751 | FJ416824.1 |
| *Helicops_boitata* | 5 | MN038112 | MN038124 | - | MN032458 | MN032471 | MN032484 | - | - | - |
| *Helicops_carinicaudus* | 5 | MN038104 | MN038125 | - | MN032450 | MN032462 | MN032475 | - | - | - |
| *Helicops_gomesi* | 5 | GQ457798 | GQ457739 | - | MN032449 | GQ457858 | MN032476 | - | - | - |
| *Helicops_hagmanni* | 5 | JQ598816 | JQ598878 | - | MN032456 | JQ598985 | MN032481 | - | - | - |
| *Helicops_infrataeniatus* | 4 | GQ457799 | GQ457740 | JQ598933 | - | GQ457859 | - | - | - | - |
| *Helicops_leopardinus* | 5 | MN038108 | MN038121 | - | MN032452 | MN032465 | MN032479 | - | - | - |
| *Helicops_modestus* | 5 | MN038110 | MN038123 | - | MN032455 | MN032469 | MN032480 | - | - | - |
| *Helicops_nentur* | 2 | - | KT453992 | - | - | KT453991 | - | - | - | - |
| *Helicops_pictiventris* | 6 | CBGM00208 | CBGM00208 | - | CBGM00208 | CBGM00208 | CBGM00208 | U49310 | - | - |
| *Helicops_polylepis* | 4 | MN038111 | - | - | MN032453 | MN032470 | MN032483 | - | - | - |
| *Heterodon_nasicus* | 6 | GQ457801 | KX277250 | KP765664 | CTMZ00154 | GQ457861 | CTMZ00154 | - | - | - |
| *Heterodon_platirhinos* | 9 | MK065495 | KX694647 | JQ598934 | JQ599028 | JQ598986 | GU353271 | AF402659 | MK198973 | MK194907 |
| *Heterodon_simus* | 6 | AY577020 | AY577029 | AF217840 | - | AF471142 | - | DQ902310 | DQ902242 | - |
| *Hydrodynastes_bicinctus* | 6 | GQ457802 | GQ457742 | JQ598935 | JQ599030 | GQ457862 | MT328102 | - | - | - |
| *Hydrodynastes_gigas* | 6 | GQ457803 | GQ457743 | GQ895873 | JQ599031 | GQ457863 | MT328127 | - | - | - |
| *Hydromorphus_concolor* | 4 | - | - | JX398641 | - | GQ895817 | JX398762 | JX398490 | - | - |
| *Hydrops_triangularis* | 6 | GQ457804 | GQ457744 | AF471039 | JQ599032 | GQ457864 | MN032474 | - | - | - |
| *Hypsiglena_affinis* | 6 | MT561499 | MT561499 | MT561499 | - | - | GU353278 | MT561499 | MT561499 | - |
| *Hypsiglena_catalinae* | 5 | KJ486459 | KJ486459 | KJ486459 | - | - | - | KJ486459 | KJ486459 | - |
| *Hypsiglena_chlorophaea* | 6 | EU728577 | EU728577 | EU728577 | - | - | FJ455197 | EU728577 | EU728577 | - |
| *Hypsiglena_jani* | 6 | EU728592 | EU728592 | EU728592 | - | - | FJ455193 | EU728592 | EU728592 | - |
| *Hypsiglena_ochrorhyncha* | 6 | EU728578 | EU728578 | EU728578 | - | - | FJ455199 | EU728578 | EU728578 | - |
| *Hypsiglena_slevini* | 6 | EU728584 | EU728584 | EU728584 | - | - | FJ455191 | EU728584 | EU728584 | - |
| *Hypsiglena_tanzeri* | 2 | - | - | EU728588 | - | - | - | EU363044 | - | - |
| *Hypsiglena_torquata* | 7 | EU728591 | EU728591 | EU728591 | - | AF471159 | FJ455192 | EU728591 | EU728591 | - |
| *Hypsiglena_unaocularus* | 5 | KJ486458 | KJ486458 | KJ486458 | - | - | - | KJ486458 | KJ486458 | - |
| *Hypsirhynchus_callilaemus* | 6 | AF158440 | AF158509 | FJ416737 | - | - | - | FJ416811 | FJ416775 | FJ416848 |
| *Hypsirhynchus_ferox* | 7 | AF158447 | AF158515 | FJ416742 | - | GQ895818 | - | FJ416816 | FJ416780 | FJ416853 |
| *Hypsirhynchus_funereus* | 6 | AF158451 | AF158520 | FJ416739 | - | - | - | FJ416813 | FJ416777 | FJ416850 |
| *Hypsirhynchus_parvifrons* | 8 | KX694557 | KX694616 | KX694883 | JQ599006 | - | KX694993 | FJ416814 | FJ416778 | FJ416851 |
| *Hypsirhynchus_polylepis* | 6 | AF158450 | AF158519 | FJ416738 | - | - | - | FJ416812 | FJ416776 | FJ416849 |
| *Hypsirhynchus_scalaris* | 6 | AF158449 | AF158518 | FJ416741 | - | - | - | FJ416815 | FJ416779 | FJ416852 |
| *Ialtris_dorsalis* | 6 | AF158456 | AF158525 | FJ416735 | - | - | - | FJ416809 | FJ416773 | FJ416846 |
| *Ialtris_haeitianus* | 6 | AF158458 | AF158527 | FJ416736 | - | - | - | FJ416810 | FJ416774 | FJ416847 |
| *Imantodes_cenchoa* | 8 | NC_013988 | NC_013988 | NC_013988 | JQ599033 | GQ457865 | KX695022 | NC_013988 | NC_013988 | - |
| *Imantodes_chocoensis* | 2 | - | - | KC176250 | - | - | - | KC176262 | - | - |
| *Imantodes_gemmistratus* | 2 | - | - | EF078509 | - | - | - | EF078557 | - | - |
| *Imantodes_inornatus* | 4 | - | MH140784 | EF078511 | - | - | GU353279 | EF078559 | - | - |
| *Imantodes_lentiferus* | 6 | 968330 | KX277251 | KC176252 | 968330 | 968330 | - | EF078561 | - | - |
| *Leptodeira_annulata* | 9 | GQ457806 | GQ457746 | EF078516 | FJ433998 | GQ457866 | FJ434099 | FJ416787 | FJ416749 | EF144108 |
| *Leptodeira_ashmeadii* | 2 | - | - | GQ334498 | - | - | - | GQ334600 | - | - |
| *Leptodeira_bakeri* | 3 | - | - | GQ334518 | - | - | GQ334673 | GQ334618 | - | - |
| *Leptodeira_frenata* | 3 | - | - | EF078532 | - | - | FJ810242 | EF078580 | - | - |
| *Leptodeira_larcorum* | 3 | - | MW208458 | - | - | - | MW208520 | MW208484 | - | - |
| *Leptodeira_maculata* | 3 | - | - | GQ334521 | - | - | GQ334674 | GQ334623 | - | - |
| *Leptodeira_misinawui* | 4 | - | MW208452 | MW208466 | - | - | MW208522 | MW208503 | - | - |
| *Leptodeira_nigrofasciata* | 3 | - | - | GQ334526 | - | - | FJ810241 | EF078581 | - | - |
| *Leptodeira_ornata* | 4 | - | MW208455 | MW208461 | - | - | MW208527 | MW208487 | - | - |
| *Leptodeira_polysticta* | 6 | EU728590 | EU728590 | EU728590 | - | - | GQ334679 | EU728590 | EU728590 | - |
| *Leptodeira_punctata* | 3 | - | - | EF078530 | - | - | GQ334682 | EF078577 | - | - |
| *Leptodeira_rhombifera* | 3 | - | - | GQ334509 | - | - | GQ334672 | GQ334611 | - | - |
| *Leptodeira_rubricata* | 2 | - | - | GQ334527 | - | - | - | GQ334631 | - | - |
| *Leptodeira_septentrionalis* | 5 | GU018148 | GU018163 | KC176243 | - | - | FJ455188 | KC176255 | - | - |
| *Leptodeira_splendida* | 3 | - | - | EF078521 | - | - | GQ334680 | EF078569 | - | - |
| *Leptodeira_uribei* | 3 | - | - | FJ810235 | - | - | FJ810243 | EF078579 | - | - |
| *Lygophis_anomalus* | 5 | JQ598817 | JQ598879 | - | CBGM00179 | CBGM00179 | CBGM00179 | - | - | - |
| *Lygophis_dilepis* | 1 | - | MN276252 | - | - | - | - | - | - | - |
| *Lygophis_elegantissimus* | 6 | GQ457808 | GQ457748 | CBGM00055 | CBGM00055 | GQ457868 | CBGM00055 | - | - | - |
| *Lygophis_flavifrenatus* | 2 | JQ598818 | JQ598880 | - | - | - | - | - | - | - |
| *Lygophis_lineatus* | 3 | - | - | - | DQ469795 | DQ469789 | DQ469793 | - | - | - |
| *Lygophis_meridionalis* | 5 | GQ457810 | GQ457750 | - | CBGM00041 | GQ457870 | CBGM00041 | - | - | - |
| *Lygophis_paucidens* | 2 | JQ598819 | - | - | - | JQ598987 | - | - | - | - |
| *Magliophis_exiguum* | 7 | FJ416694 | FJ416705 | FJ416724 | - | AF471117 | - | FJ416798 | FJ416762 | FJ416835 |
| *Magliophis_stahli* | 4 | - | - | FJ416725 | - | - | - | FJ416799 | FJ416763 | FJ416836 |
| *Manolepis_putnami* | 5 | JQ598820 | JQ598881 | JQ598936 | JQ599035 | JQ598988 | - | - | - | - |
| *Mussurana_bicolor* | 5 | GQ457787 | GQ457729 | - | CTMZ00230 | GQ457849 | CTMZ00230 | - | - | - |
| *Ninia_atrata* | 7 | GQ457814 | KX694675 | JQ598937 | JQ599037 | GQ457874 | GQ334683 | GQ334659 | - | - |
| *Ninia_diademata* | 2 | - | - | JX398645 | - | - | JX398764 | - | - | - |
| *Ninia_maculata* | 1 | - | MH140876 | - | - | - | - | - | - | - |
| *Ninia_sebae* | 3 | - | - | GQ895879 | - | GQ895821 | MH823659 | - | - | - |
| *Nothopsis_rugosus* | 6 | GU018159 | KR814760 | KR814770 | - | KR814768 | JX398765 | JX398494 | - | - |
| *Oxyrhopus_clathratus* | 5 | GQ457815 | GQ457754 | - | CBGM00183 | GQ457875 | CBGM00183 | - | - | - |
| *Oxyrhopus_fitzingeri* | 5 | CTMZ04839 | KX660272 | KX660541 | - | KX660409 | KX652083 | - | - | - |
| *Oxyrhopus_formosus* | 6 | JQ598821 | KX660249 | KX660543 | CBGM00090 | KX660411 | KX652085 | - | - | - |
| *Oxyrhopus_guibei* | 6 | JQ598822 | JQ598883 | JQ598938 | JQ599038 | JQ598989 | YPX132 | - | - | - |
| *Oxyrhopus_melanogenys* | 5 | JQ598823 | KX277264 | - | CBGM00064 | JQ598990 | CBGM00064 | - | - | - |
| *Oxyrhopus_petola* | 7 | OXPE001 | GU018162 | GQ334554 | CBGM00149 | OXPE001 | GQ334684 | GQ334660 | - | - |
| *Oxyrhopus_rhombifer* | 5 | GQ457816 | GQ457755 | - | CBGM00210 | GQ457876 | CBGM00210 | - | - | - |
| *Oxyrhopus_trigeminus* | 5 | JQ598824 | JQ598884 | JQ598939 | - | - | KX652086 | KX660653 | - | - |
| *Paraphimophis_rusticus* | 4 | JQ598802 | JQ598864 | JQ598923 | - | JQ598974 | - | - | - | - |
| *Phalotris_lativittatus* | 5 | JQ598825 | JQ598885 | - | CTMZ00627 | JQ598991 | CTMZ00627 | - | - | - |
| *Phalotris_lemniscatus* | 5 | GQ457817 | GQ457756 | JQ598941 | JQ599039 | GQ457877 | - | - | - | - |
| *Phalotris_mertensi* | 2 | JQ598826 | JQ598886 | - | - | - | - | - | - | - |
| *Phalotris_nasutus* | 4 | GQ457818 | GQ457757 | JQ598942 | - | GQ457878 | - | - | - | - |
| *Phalotris_reticulatus* | 3 | JQ598827 | JQ598887 | JQ598943 | - | - | - | - | - | - |
| *Philodryas_aestiva* | 6 | GQ457819 | GQ457758 | AF236809 | CBGM00079 | GQ457879 | CBGM00079 | - | - | - |
| *Philodryas_agassizii* | 6 | GQ457823 | GQ457762 | GQ895883 | CBGM00080 | GQ457883 | CBGM00080 | - | - | - |
| *Philodryas_argentea* | 5 | GQ457842 | GQ457780 | JQ598944 | JQ599040 | GQ457899 | - | - | - | - |
| *Philodryas_baroni* | 5 | JQ598828 | JQ598888 | AF236812 | - | MH122715 | - | MH122686 | - | - |
| *Philodryas_chamissonis* | 1 | - | - | - | - | - | - | HM639960 | - | - |
| *Philodryas_georgeboulengeri* | 5 | MK287607 | MK287616 | MK282824 | MK282807 | MK282816 | - | - | - | - |
| *Philodryas_laticeps* | 1 | - | MK287608 | - | - | - | - | - | - | - |
| *Philodryas_mattogrossensis* | 5 | GQ457820 | GQ457759 | - | CBGM00044 | GQ457880 | CBGM00044 | - | - | - |
| *Philodryas_nattereri* | 6 | JQ598829 | JQ598889 | AF236806 | CBGM00220 | JQ598992 | CBGM00220 | - | - | - |
| *Philodryas_olfersii* | 6 | JQ598830 | JQ598890 | JQ598945 | JQ599041 | JQ598993 | YPX134 | - | - | - |
| *Philodryas_patagoniensis* | 4 | GQ457821 | GQ457760 | AF236808 | - | GQ457881 | - | - | - | - |
| *Philodryas_psammophidea* | 2 | GU018149 | GU018168 | - | - | - | - | - | - | - |
| *Philodryas_trilineata* | 3 | PHTR001err | PHTRL001err | - | - | - | - | HM639963 | - | - |
| *Philodryas_viridissima* | 3 | AF158419 | AF158474 | AF236807 | - | - | - | - | - | - |
| *Phimophis_guerini* | 3 | GQ457822 | GQ457761 | - | - | GQ457882 | - | - | - | - |
| *Phimophis_vittatus* | 1 | - | MN276285 | - | - | - | - | - | - | - |
| *Pliocercus_elapoides* | 4 | - | - | GQ895882 | - | GQ895824 | JX398766 | JX398495 | - | - |
| *Pliocercus_euryzonus_merged* | 7 | TG2201 | TG2201 | KX660440 | TG2201 | TG2201 | TG2201 | KX660569.1 | - | - |
| *Pseudalsophis_biserialis_San_Cristobal_CTMZ4659* | 6 | CTMZ4659 | CTMZ4659 | CTMZ4659 | CTMZ4659 | CTMZ4659 | CTMZ4659 | - | - | - |
| *Pseudalsophis_darwini_Isabela_CTMZ4721* | 6 | CTMZ4721 | CTMZ4721 | CTMZ4721 | CTMZ4721 | CTMZ4721 | CTMZ4721 | - | - | - |
| *Pseudalsophis_dorsalis_Santa_Fe_CTMZ4647* | 6 | CTMZ4647 | CTMZ4647 | CTMZ4647 | CTMZ4647 | CTMZ4647 | CTMZ4647 | - | - | - |
| *Pseudalsophis_elegans_CTMZ7428* | 6 | CTMZ7428 | CTMZ7428 | CTMZ7428 | CTMZ7428 | CTMZ7428 | CTMZ7428 | - | - | - |
| *Pseudalsophis_hephaestus_Santiago_CTMZ4725* | 6 | CTMZ4725 | CTMZ4725 | CTMZ4725 | CTMZ4725 | CTMZ4725 | CTMZ4725 | - | - | - |
| *Pseudalsophis_hoodensis_Espanola_CTMZ4657* | 6 | CTMZ4657 | CTMZ4657 | CTMZ4657 | CTMZ4657 | CTMZ4657 | CTMZ4657 | - | - | - |
| *Pseudalsophis_occidentalis_Tortuga_CTMZ4665* | 6 | CTMZ4665 | CTMZ4665 | CTMZ4665 | CTMZ4665 | CTMZ4665 | CTMZ4665 | - | - | - |
| *Pseudalsophis_slevini_Pinzon_CTMZ4730* | 6 | CTMZ4730 | CTMZ4730 | CTMZ4730 | CTMZ4730 | CTMZ4730 | CTMZ4730 | - | - | - |
| *Pseudalsophis_steindachneri_Santa_Cruz_CTMZ4740* | 6 | CTMZ4740 | CTMZ4740, si hay secuencia pero corta | CTMZ4740, si hay secuencia | CTMZ4740 | CTMZ4740 | CTMZ4740 | - | - | - |
| *Pseudalsophis_thomasi_Rabida_CTMZ4727* | 6 | CTMZ4727 | CTMZ4727 | CTMZ4727 | CTMZ4727 | CTMZ4727 | CTMZ4727 | - | - | - |
| *Pseudoboa_coronata* | 4 | GQ457824 | GQ457763 | - | - | GQ457884 | CTMZ00538 | - | - | - |
| *Pseudoboa_neuwiedii* | 4 | AF158423 | AF158490 | GQ895884 | - | GQ895825 | - | - | - | - |
| *Pseudoboa_nigra* | 6 | GQ457825 | GQ457764 | JQ598948 | JQ599043 | GQ457885 | CTMZ00036 | - | - | - |
| *Pseudoeryx_plicatilis* | 6 | GQ457826 | GQ457765 | GQ895885 | MN032447 | GQ457886 | MN032473 | - | - | - |
| *Pseudoleptodeira_latifasciata* | 6 | EU728579 | EU728579 | EU728579 | - | - | FJ455190 | EU728579 | EU728579 | - |
| *Pseudotomodon_trigonatus* | 4 | GQ457827 | GQ457766 | - | - | GQ457887 | CTMZ00238 | - | - | - |
| *Psomophis_genimaculatus* | 3 | GQ457828 | GQ457767 | - | - | GQ457888 | - | - | - | - |
| *Psomophis_joberti* | 6 | GQ457829 | GQ457768 | JQ598950 | JQ599046 | GQ457889 | KX695043 | - | - | - |
| *Psomophis_obtusus* | 2 | JQ598836 | JQ598896 | - | - | - | - | - | - | - |
| *Ptychophis_flavovirgatus* | 4 | GQ457830 | GQ457769 | - | - | GQ457890 | YPX138 | - | - | - |
| *Rhachidelus_brazili* | 6 | JQ598837 | JQ598897 | JQ598952 | JQ599048 | CTMZ00714 | KX695045 | - | - | - |
| *Rhadinaea_decorata* | 2 | - | MH140925 | MT308780 | - | - | - | - | - | - |
| *Rhadinaea_flavilata* | 2 | - | - | AF471078 | - | AF471152 | - | - | - | - |
| *Rhadinaea_fulvivittis* | 3 | - | - | EF078539 | - | - | GU353275 | EF078587 | - | - |
| *Rhadinaea_laureata* | 1 | - | - | MT308785 | - | - | - | - | - | - |
| *Rhadinaea_pulveriventris* | 3 | - | - | JX398649 | - | - | JX398768 | JX398497 | - | - |
| *Rhadinaea_taeniata* | 1 | - | - | MT308788 | - | - | - | - | - | - |
| *Rhadinaea_vermiculaticeps* | 1 | - | MH140938 | - | - | - | - | - | - | - |
| *Rhadinella_hempsteadae* | 1 | - | - | MT308783 | - | - | - | - | - | - |
| *Rhadinella_lachrymans* | 1 | - | - | MT308784 | - | - | - | - | - | - |
| *Rhadinella_stadelmani* | 1 | - | - | MT308786 | - | - | - | - | - | - |
| *Rhadinophanes_monticola* | 3 | - | - | JX398650 | - | - | JX398769 | JX398498 | - | - |
| *Rodriguesophis_chui* | 1 | - | - | MK548582 | - | - | - | - | - | - |
| *Rodriguesophis_iglesiasi* | 4 | JQ598831 | JQ598891 | GQ895881 | - | GQ895823 | - | - | - | - |
| *Rodriguesophis_scriptorcibatus* | 1 | - | - | MK548583 | - | - | - | - | - | - |
| *Saphenophis_antioquiensis* | 6 | CTMZ19640 | CTMZ19640 | CTMZ19640 | CTMZ19640 | CTMZ19640 | CTMZ19640 | - | - | - |
| *Sibon_annulatus* | 6 | MH341097 | KX660170 | KX660443 | - | KX660308 | KX651997 | KX660572 | - | - |
| *Sibon_anthracops* | 6 | MH341098.1 | MH341076.1 | MH375035.1 | - | MH374957.1 | JX398779 | MH375054.1 | - | - |
| *Sibon_argus* | 4 | - | MH140955 | JX398659 | - | - | JX398780 | JX398507 | - | - |
| *Sibon_bevridgelyi* | 2 | - | MH341078.1 | MH374963.1 | - | - | - | - | - | - |
| *Sibon_carri* | 3 | - | - | JX398664 | - | - | JX398785 | JX398513 | - | - |
| *Sibon_dimidiatus* | 5 | - | KX660278 | JX398669 | - | KX660417 | KX652093 | KX660658 | - | - |
| *Sibon_dunni* | 2 | - | MH341079.1 | MH374991.1 | - | - | - | - | - | - |
| *Sibon_lamari* | 4 | - | MH140964 | JX398670 | - | - | JX398791 | JX398519 | - | - |
| *Sibon_longifrenis* | 6 | MH341099.1 | MH341080.1 | MH375036.1 | - | MH374958.1 | JX398792. | MH375055.1 | - | - |
| *Sibon_manzanaresi* | 3 | - | - | JX398686 | - | - | JX398796 | JX398525 | - | - |
| *Sibon_merendonensis* | 6 | MH341100.1 | MH341081.1 | MH375037.1 | - | MH374959.1 | JX398797 | MH375056.1 | - | - |
| *Sibon_miskitus* | 3 | - | - | JX398676 | - | - | JX398798 | JX398527 | - | - |
| *Sibon_nebulatus* | 7 | EU728583 | EU728583 | EU728583 | - | AF544736 | FJ455189 | EU728583 | EU728583 | - |
| *Sibon_noalamina* | 1 | - | KP209376 | - | - | - | - | - | - | - |
| *Sibon_perissostichon* | 3 | - | - | JX398688 | - | - | JX398814 | JX398552 | - | - |
| *Sibon_sanniolus* | 3 | - | - | JX398692 | - | - | JX398815 | JX398553 | - | - |
| *Sibynomorphus_garmani* | 4 | GQ457831 | GQ457770 | - | CBGM00232 | GQ457891 | - | - | - | - |
| *Sibynomorphus_mikanii* | 6 | GQ457832 | GQ457771 | JQ598954 | JQ599050 | GQ457892 | KX695048 | - | - | - |
| *Sibynomorphus_neuwiedi* | 5 | JQ598838 | JQ598898 | - | CBGM00061 | CBGM00061 | CBGM00061 | - | - | - |
| *Sibynomorphus_turgidus* | 6 | JQ598839 | JQ598899 | KX660547 | - | KX660418 | KX652094 | KX660659 | - | - |
| *Sibynomorphus_vagus* | 2 | - | KX660252 | - | - | KX660393 | - | - | - | - |
| *Sibynomorphus_ventrimaculatus* | 6 | JQ598840 | JQ598900 | CBGM00231 | CBGM00231 | JQ598997 | CBGM00231 | - | - | - |
| *Siphlophis_ayauma* | 1 | - | JX406878 | - | - | - | - | - | - | - |
| *Siphlophis_cervinus* | 6 | JQ598841 | JQ598901 | GQ895888 | CBGM00033 | JQ598998 | CBGM00033 | - | - | - |
| *Siphlophis_compressus* | 6 | GQ457833 | GQ457772 | GQ895894 | CBGM00035 | GQ457893 | CBGM00035 | - | - | - |
| *Siphlophis_longicaudatus* | 4 | JQ598842 | JQ598902 | - | CTMZ00655 | JQ598999 | - | - | - | - |
| *Siphlophis_pulcher* | 5 | GQ457834 | GQ457773 | JQ598955 | JQ599051 | GQ457894 | - | - | - | - |
| *Sordellina_punctata* | 6 | JQ598843 | JQ598903 | JQ598956 | JQ599052 | JQ599000 | YPX143 | - | - | - |
| *Stichophanes_ningshaanensis* | 6 | KJ719252 | KJ719252 | KJ719252 | - | KJ638717 | - | KJ719252 | KJ719252 | - |
| *Synophis_bicolor* | 6 | - | CTMZ0854 | KR814771 | CTMZ0854 | KR814762 | CTMZ0854 | KR814780.1 | - | - |
| *Synophis_bogerti* | 3 | - | KT345348 | KT345365 | - | - | - | KT345382.1 | - | - |
| *Synophis_calamitus* | 5 | KR814622 | KR814640 | KR814772 | - | KR814663 | - | KR814711.1 | - | - |
| *Synophis_insulomontanus* | 3 | - | KT345349 | KT345366 | - | - | - | KT345383.1 | - | - |
| *Synophis_lasallei* | 3 | - | KT944047 | - | - | KT944068 | - | KT944064 | - | - |
| *Synophis_niceforomariae* | 6 | KR814751 | KR814758 | KR814773 | KX672829 | KR814769 | KX672831 | - | - | - |
| *Synophis_zaheri* | 3 | - | KR814756 | - | - | KR814763 | - | KR814776.1 | - | - |
| *Synophis_zamora* | 3 | - | KT345347 | KT345364 | - | - | - | KT345381 | - | - |
| *Tachymenis_chilensis* | 1 | - | - | - | - | - | - | HM639913 | - | - |
| *Tachymenis_peruviana* | 5 | GQ457835 | GQ457774 | - | JQ599054 | GQ457895 | CBGM00063 | - | - | - |
| *Taeniophallus_affinis* | 5 | JQ598844 | JQ598905 | JQ598957 | JQ599055 | GQ457853 | - | - | - | - |
| *Taeniophallus_brevirostris* | 5 | GQ457793 | GQ457734 | JQ598958 | JQ599056 | GQ457854 | - | - | - | - |
| *Taeniophallus_nicagus* | 6 | JQ598845 | JQ598906 | KX694886 | KX694766 | JQ599001 | KX695053 | - | - | - |
| *Tantalophis_discolor* | 3 | - | - | EF078541 | - | - | FJ810240 | EF078589 | - | - |
| *Thamnodynastes_chaquensis* | 1 | - | MN276309 | - | - | - | - | - | - | - |
| *Thamnodynastes_hypoconia* | 7 | JQ598846 | CTMZ00462 | KX660523 | CTMZ00462 | KX660394 | CTMZ00462 | KX660643 | - | - |
| *Thamnodynastes_lanei* | 2 | GQ457836 | GQ457775 | - | - | - | - | - | - | - |
| *Thamnodynastes_pallidus* | 4 | GU018155 | KX277270 | GQ895891 | - | GQ895832 | - | - | - | - |
| *Thamnodynastes_rutilus* | 5 | GQ457837 | GQ457776 | - | CBGM00051 | GQ457896 | CBGM00051 | - | - | - |
| *Thamnodynastes_strigatus* | 6 | JQ598847 | JQ598907 | JQ598959 | JQ599057 | KX660420 | KX652096 | - | - | - |
| *Thermophis_baileyi* | 9 | MF326642 | MF326642 | MF326642 | CTMZ07596 | CTMZ07596 | CTMZ07596 | MF326642 | MF326642 | MK194860 |
| *Thermophis_shangrila* | 7 | NC_035058 | NC_035058 | NC_035058 | - | KF514883 | - | NC_035058 | NC_035058 | MK194857 |
| *Thermophis_zhaoermii* | 7 | GQ166168 | GQ166168 | GQ166168 | - | KP777529 | - | GQ166168 | GQ166168 | MK194858 |
| *Tomodon_dorsatus* | 6 | GQ457838 | GQ457777 | JQ598960 | JQ599059 | GQ457897 | KX695055 | - | - | - |
| *Tretanorhinus_mocquardi* | 1 | - | MH141000 | - | - | - | - | - | - | - |
| *Tretanorhinus_nigroluteus* | 2 | - | - | GQ895893 | - | GQ895834 | - | - | - | - |
| *Tretanorhinus_variabilis* | 5 | AF158460 | AF158529 | JX398722 | - | - | JX398837 | JX398592 | - | - |
| *Trimetopon_gracile* | 5 | GU018160 | GU018178 | JX398723 | - | - | JX398838 | JX398593 | - | - |
| *Tropidodipsas_annulifera* | 3 | - | - | JX398698 | - | - | JX398824 | JX398558 | - | - |
| *Tropidodipsas_fasciata* | 6 | MH341101.1 | MH341085.1 | MH375027.1 | - | MH374961.1 | JX398828 | MH375057.1 | - | - |
| *Tropidodipsas_fischeri* | 6 | MH341102 | KX660289 | KX660553 | - | KX660428 | KX652100 | KX660664 | - | - |
| *Tropidodipsas_philippii* | 3 | - | - | JX398710 | - | - | JX398826 | JX398570 | - | - |
| *Tropidodipsas_sartorii* | 3 | - | - | EF078540 | - | - | JX398834 | EF078588 | - | - |
| *Tropidodryas_serra* | 5 | JQ598848 | CTMZ-00717 | JQ598961 | - | CTMZ-00717 | CTMZ-00717 | - | - | - |
| *Tropidodryas_striaticeps* | 6 | GQ457839 | GQ457778 | YPX149 | JQ599060 | CTMZ00185 | CTMZ00185 | - | - | - |
| *Uromacer_catesbyi* | 6 | AF158454 | AF158523 | FJ416714 | - | - | - | FJ416788 | FJ416752 | FJ416825 |
| *Uromacer_frenatus* | 6 | AF158444 | AF158513 | FJ416715 | - | - | - | FJ416789 | FJ416753 | FJ416826 |
| *Uromacer_oxyrhynchus* | 6 | FJ416701 | FJ416712 | FJ416716 | - | - | - | FJ416790 | FJ416754 | FJ416827 |
| *Urotheca_decipiens* | 3 | - | - | JX398725 | - | - | JX398840 | JX398595 | - | - |
| *Urotheca_fulviceps* | 1 | - | MH141002 | - | - | - | - | - | - | - |
| *Urotheca_guentheri* | 3 | - | - | JX398726 | - | - | JX398841 | JX398596 | - | - |
| *Urotheca_pachyura* | 1 | - | MH141003 | - | - | - | - | - | - | - |
| *Xenodon_dorbignyi* | 5 | GQ457812 | GQ457752 | - | CBGM00202 | CBGM00202 | CBGM00202 | - | - | - |
| *Xenodon_guentheri* | 5 | JQ598849 | JQ598909 | - | CBGM00058 | CBGM00058 | CBGM00058 | - | - | - |
| *Xenodon_histricus* | 6 | GQ457813 | GQ457753 | JQ598962 | JQ599061 | GQ457873 | CBGM00066 | - | - | - |
| *Xenodon_matogrossensis* | 5 | JQ598850 | JQ598910 | - | CBGM00001 | CBGM00001 | CBGM00001 | - | - | - |
| *Xenodon_merremii* | 6 | GQ457840 | JQ598911 | JQ598963 | JQ599062 | CBGM00178 | YPX150 | - | - | - |
| *Xenodon_nattereri* | 5 | JQ598851 | JQ598912 | - | CBGM00109 | CBGM00109 | CBGM00109 | - | - | - |
| *Xenodon_neuwiedii* | 6 | GQ457841 | GQ457779 | AF236814 | CBGM00199 | CBGM00199 | CBGM00199 | - | - | - |
| *Xenodon_pulcher* | 4 | JQ598852 | JQ598913 | - | CBGM00078 | - | CBGM00078 | - | - | - |
| *Xenodon_rabdocephalus* | 4 | - | MH141007 | JX398727 | - | - | JX398842 | JX398597 | - | - |
| *Xenodon_semicinctus* | 3 | GU018156 | GU018173 | GQ895877 | - | - | - | - | - | - |
| *Xenodon_severus* | 5 | JQ598853 | JQ598914 | JQ598964 | JQ599063 | - | YPX151 | - | - | - |
| *Xenodon_werneri* | 2 | AF158468 | AF158538 | - | - | - | - | - | - | - |
| *Xenopholis_scalaris* | 4 | JQ598854 | JQ598915 | GQ895897 | - | JQ599002 | - | - | - | - |
| *Xenopholis_undulatus* | 3 | JQ598855 | JQ598916 | - | - | JQ599003 | - | - | - | - |
| *Bungarus_fasciatus* | 9 | EU579523 | EU579523 | EU579523 | JQ599013 | AF544732_AY058924_YPX591 | YPX591 | EU579523 | EU579523 | EF144100.1 |
| *Calliophis_maculiceps* | 5 | - | YPX592 | YPX592 | YPX592 | YPX592 | YPX592 | - | - | - |
| *Micrurus_surinamensis* | 8 | AF544770 | AF544799 | EF137415 | FJ433991 | EF137422_AF544708 | FJ434092 | AF228444 | - | EF144102.1 |
| *Eryx_conicus* | 6 | AF512743 | AF512743 | GQ225658 | AY988040 | GQ225672 | YPX046 | - | - | - |
| *Homalopsis_buccata* | 7 | AF499288_YPX560 | EF395868_YPX560 | EF395917 | YPX560 | EF395940_AF544701_YPX560 | YPX560 | - | - | EF144097.1 |
| *Natrix_natrix* | 8 | KF781319 | AF158530 | AY866541 | JQ599036 | AF471121_AF544697_YPX539 | KF234022 | AY873736 | AY870617.1 | - |
| *Rhabdophis_subminiatus* | 7 | AF544776_YPX566 | AF544805 | JQ598951 | JQ599047 | YPX566 | YPX566 | JQ687411 | - | - |
| *Aplopeltura_boa* | 7 | AF544761 | AF544787 | JF827673 | FJ433984 | AF544715 | FJ434085 | JF827650 | - | - |
| *Pareas_carinatus* | 8 | AF544773 | AF544802 | JQ598940 | FJ433985 | AF544692 | FJ434086 | JF827652 | - | EF144096.1 |
| *Rhamphiophis_oxyrhynchus* | 7 | YPX523 | YPX523 | JQ598953 | JQ599049 | FJ387213_AF544710_YPX523 | YPX523 | - | - | FJ404400.1 |
| *Pseudoxenodon_bambusicola* | 7 | JQ598833 | JQ598893 | - | JQ599044 | JQ598996 | FJ434101 | - | MK198926 | EF144111.1 |
| *Pseudoxenodon_karlschmidti* | 8 | JQ598834 | JQ598894 | AF471080 | JQ599045 | AF471102 | KX695042 | - | MK198924 | MK194832 |
| *Azemiops_feae* | 9 | KJ872487 | KJ872487 | KJ872487 | EU402628 | YPX807 | YPX807 | KJ872487 | KJ872487.1 | MK195041 |
| *Bothriechis_schlegelii* | 9 | AF057213 | AF057260 | AY223590 | FJ433983 | AF544680 | FJ434084 | AF292611 | MK313460 | EF144095.1 |
| *Causus_lichtensteinii* | 6 | YPX796 | YPX796 | YPX796 | YPX796 | YPX796 | YPX796 | - | - | - |
| *Macrovipera_lebetina* | 8 | YPX817 | YPX817 | AJ275713 | YPX817 | YPX817 | YPX817 | DQ897729 | MT232996 | - |
| *Achalinus_rufescens* | 8 | YPX105 | YPX105 | YPX547 | YPX105 | - | YPX105 | U49319 | KT897595.1 | MK195122 |
| *Xenodermus_javanicus* | 6 | AF544781 | AF544810 | AY425810 | EU402667 | AF544711 | - | U49320 | - | - |
| **Total number of sequences per gene** | | 269 | 343 | 303 | 152 | 218 | 251 | 202 | 69 | 54 |

**Table S2.** Taxonomic arrangement of the Dipsadidae used for the TACT analyses performed for this work, following Uetz et al. (2023). Taxa whose taxonomic assignment differs from this database are indicated in bold, and a justification is provided in a separate column. Please note that this does not mean we do not endorse Uetz et al. (2023)’s taxonomy. Instead, modifications from Uetz et al. (2022)’s taxonomy were made to minimize the impact on our TACT analysis of the few taxa which most likely were recovered in an erroneous phylogenetic position in the Bayesian calibrated time tree we inferred herein.

| **Family** | **Subfamily** | **Tribe** | **Genus** | **Species** | **Comments** |
| --- | --- | --- | --- | --- | --- |
| Dipsadidae | Dipsadinae | Tribenov.1 | *Adelphicos* | *Adelphicos daryi* | Tribal classification following Sheehy (2012) |
| Dipsadidae | Dipsadinae | Tribenov.1 | *Adelphicos* | *Adelphicos ibarrorum* | Tribal classification following Sheehy (2012) |
| Dipsadidae | Dipsadinae | Tribenov.1 | *Adelphicos* | *Adelphicos latifasciatum* | Tribal classification following Sheehy (2012) |
| Dipsadidae | Dipsadinae | Tribenov.1 | *Adelphicos* | *Adelphicos newmanorum* | Tribal classification following Sheehy (2012) |
| Dipsadidae | Dipsadinae | Tribenov.1 | *Adelphicos* | *Adelphicos nigrilatum* | Tribal classification following Sheehy (2012) |
| Dipsadidae | Dipsadinae | Tribenov.1 | *Adelphicos* | *Adelphicos quadrivirgatum* | Tribal classification following Sheehy (2012) |
| Dipsadidae | Dipsadinae | Tribenov.1 | *Adelphicos* | *Adelphicos sargii* | Tribal classification following Sheehy (2012) |
| Dipsadidae | Dipsadinae | Tribenov.1 | *Adelphicos* | *Adelphicos veraepacis* | Tribal classification following Sheehy (2012) |
| Dipsadidae | Dipsadinae | Tribenov.1 | *Adelphicos* | *Adelphicos visoninum* | Tribal classification following Sheehy (2012) |
| Dipsadidae | Xenodontinae | Echinantherini | *Adelphostigma* | *Adelphostigma occipitalis* | Generic classification following Abegg et al. (2022) |
| Dipsadidae | Xenodontinae | Echinantherini | *Adelphostigma* | *Adelphostigma quadriocellata* | Generic classification following Abegg et al. (2022) |
| Dipsadidae | Xenodontinae | Alsophini | *Alsophis* | *Alsophis antiguae* |  |
| Dipsadidae | Xenodontinae | Alsophini | *Alsophis* | *Alsophis antillensis* |  |
| Dipsadidae | Xenodontinae | Alsophini | *Alsophis* | *Alsophis danforthi* |  |
| Dipsadidae | Xenodontinae | Alsophini | *Alsophis* | *Alsophis manselli* |  |
| Dipsadidae | Xenodontinae | Alsophini | *Alsophis* | *Alsophis rijgersmaei* |  |
| Dipsadidae | Xenodontinae | Alsophini | *Alsophis* | *Alsophis rufiventris* |  |
| Dipsadidae | Xenodontinae | Alsophini | *Alsophis* | *Alsophis sanctonum* |  |
| Dipsadidae | Xenodontinae | Alsophini | *Alsophis* | *Alsophis sibonius* |  |
| Dipsadidae | Dipsadinae | Tribenov.3 | *Amastridium* | *Amastridium sapperi* | Tribal classification following Sheehy (2012) |
| Dipsadidae | Dipsadinae | Tribenov.3 | *Amastridium* | *Amastridium veliferum* | Tribal classification following Sheehy (2012) |
| Dipsadidae | Dipsadinae | Amnesteophiini | *Amnesteophis* | *Amnesteophis melanauchen* | Tribal classification following Myers (2011) and Moraes et al. (2021) |
| Dipsadidae | Xenodontinae | Echinantherini | *Amnisiophis* | *Amnisiophis amoena* | Generic classification following Abegg et al. (2022) |
| Dipsadidae | Xenodontinae | Tachymenini | *Apographon* | *Apographon orestes* | Generic classification following Trevine et al. (2022) |
| Dipsadidae | Xenodontinae | Elapomorphini | *Apostolepis* | *Apostolepis adhara* |  |
| Dipsadidae | Xenodontinae | Elapomorphini | *Apostolepis* | *Apostolepis albicollaris* |  |
| Dipsadidae | Xenodontinae | Elapomorphini | *Apostolepis* | *Apostolepis ambiniger* |  |
| Dipsadidae | Xenodontinae | Elapomorphini | *Apostolepis* | *Apostolepis arenaria* |  |
| Dipsadidae | Xenodontinae | Elapomorphini | *Apostolepis* | *Apostolepis assimilis* |  |
| Dipsadidae | Xenodontinae | Elapomorphini | *Apostolepis* | *Apostolepis borellii* |  |
| Dipsadidae | Xenodontinae | Elapomorphini | *Apostolepis* | *Apostolepis breviceps* |  |
| Dipsadidae | Xenodontinae | Elapomorphini | *Apostolepis* | *Apostolepis cearensis* |  |
| Dipsadidae | Xenodontinae | Elapomorphini | *Apostolepis* | *Apostolepis cerradoensis* |  |
| Dipsadidae | Xenodontinae | Elapomorphini | *Apostolepis* | *Apostolepis christineae* |  |
| Dipsadidae | Xenodontinae | Elapomorphini | *Apostolepis* | *Apostolepis dimidiata* |  |
| Dipsadidae | Xenodontinae | Elapomorphini | *Apostolepis* | *Apostolepis dorbignyi* |  |
| Dipsadidae | Xenodontinae | Elapomorphini | *Apostolepis* | *Apostolepis flavotorquata* |  |
| Dipsadidae | Xenodontinae | Elapomorphini | *Apostolepis* | *Apostolepis gaboi* |  |
| Dipsadidae | Xenodontinae | Elapomorphini | *Apostolepis* | *Apostolepis goiasensis* |  |
| Dipsadidae | Xenodontinae | Elapomorphini | *Apostolepis* | *Apostolepis intermedia* |  |
| Dipsadidae | Xenodontinae | Elapomorphini | *Apostolepis* | *Apostolepis kikoi* |  |
| Dipsadidae | Xenodontinae | Elapomorphini | *Apostolepis* | *Apostolepis lineata* |  |
| Dipsadidae | Xenodontinae | Elapomorphini | *Apostolepis* | *Apostolepis longicaudata* |  |
| Dipsadidae | Xenodontinae | Elapomorphini | *Apostolepis* | *Apostolepis multicincta* |  |
| Dipsadidae | Xenodontinae | Elapomorphini | *Apostolepis* | *Apostolepis nelsonjorgei* |  |
| Dipsadidae | Xenodontinae | Elapomorphini | *Apostolepis* | *Apostolepis niceforoi* |  |
| Dipsadidae | Xenodontinae | Elapomorphini | *Apostolepis* | *Apostolepis nigrolineata* |  |
| Dipsadidae | Xenodontinae | Elapomorphini | *Apostolepis* | *Apostolepis nigroterminata* |  |
| Dipsadidae | Xenodontinae | Elapomorphini | *Apostolepis* | *Apostolepis phillipsae* |  |
| Dipsadidae | Xenodontinae | Elapomorphini | *Apostolepis* | *Apostolepis polylepis* |  |
| Dipsadidae | Xenodontinae | Elapomorphini | *Apostolepis* | *Apostolepis pymi* |  |
| Dipsadidae | Xenodontinae | Elapomorphini | *Apostolepis* | *Apostolepis quirogai* |  |
| Dipsadidae | Xenodontinae | Elapomorphini | *Apostolepis* | *Apostolepis rondoni* |  |
| Dipsadidae | Xenodontinae | Elapomorphini | *Apostolepis* | *Apostolepis sanctaeritae* |  |
| Dipsadidae | Xenodontinae | Elapomorphini | *Apostolepis* | *Apostolepis serrana* |  |
| Dipsadidae | Xenodontinae | Elapomorphini | *Apostolepis* | *Apostolepis striata* |  |
| Dipsadidae | Xenodontinae | Elapomorphini | *Apostolepis* | *Apostolepis tenuis* |  |
| Dipsadidae | Xenodontinae | Elapomorphini | *Apostolepis* | *Apostolepis thalesdelemai* |  |
| Dipsadidae | Xenodontinae | Elapomorphini | *Apostolepis* | *Apostolepis underwoodi* |  |
| Dipsadidae | Xenodontinae | Elapomorphini | *Apostolepis* | *Apostolepis vittata* |  |
| Dipsadidae | Xenodontinae | Eutrachelophiini | *Arcanumophis* | *Arcanumophis problematicus* | Generic classification following Smaga et al. (2019); Tribal classification following Moraes et al. (2021) |
| Dipsadidae | Xenodontinae | Alsophini | *Arrhyton* | *Arrhyton ainictum* |  |
| Dipsadidae | Xenodontinae | Alsophini | *Arrhyton* | *Arrhyton albicollum* |  |
| Dipsadidae | Xenodontinae | Alsophini | *Arrhyton* | *Arrhyton dolichura* |  |
| Dipsadidae | Xenodontinae | Alsophini | *Arrhyton* | *Arrhyton procerum* |  |
| Dipsadidae | Xenodontinae | Alsophini | *Arrhyton* | *Arrhyton redimitum* |  |
| Dipsadidae | Xenodontinae | Alsophini | *Arrhyton* | *Arrhyton supernum* |  |
| Dipsadidae | Xenodontinae | Alsophini | *Arrhyton* | *Arrhyton taeniatum* |  |
| Dipsadidae | Xenodontinae | Alsophini | *Arrhyton* | *Arrhyton tanyplectum* |  |
| Dipsadidae | Xenodontinae | Alsophini | *Arrhyton* | *Arrhyton vittatum* |  |
| Dipsadidae | Dipsadinae | Tribenov.2 | *Atractus* | *Atractus aboiporu* | Tribal classification following Sheehy (2012) |
| Dipsadidae | Dipsadinae | Tribenov.2 | *Atractus* | *Atractus acheronius* | Tribal classification following Sheehy (2012) |
| Dipsadidae | Dipsadinae | Tribenov.2 | *Atractus* | *Atractus akerios* | Tribal classification following Sheehy (2012) |
| Dipsadidae | Dipsadinae | Tribenov.2 | *Atractus* | *Atractus albuquerquei* | Tribal classification following Sheehy (2012) |
| Dipsadidae | Dipsadinae | Tribenov.2 | *Atractus* | *Atractus alphonsehogei* | Tribal classification following Sheehy (2012) |
| Dipsadidae | Dipsadinae | Tribenov.2 | *Atractus* | *Atractus altagratiae* | Tribal classification following Sheehy (2012) |
| Dipsadidae | Dipsadinae | Tribenov.2 | *Atractus* | *Atractus alytogrammus* | Tribal classification following Sheehy (2012) |
| Dipsadidae | Dipsadinae | Tribenov.2 | *Atractus* | *Atractus andinus* | Tribal classification following Sheehy (2012) |
| Dipsadidae | Dipsadinae | Tribenov.2 | *Atractus* | *Atractus apophis* | Tribal classification following Sheehy (2012) |
| Dipsadidae | Dipsadinae | Tribenov.2 | *Atractus* | *Atractus arangoi* | Tribal classification following Sheehy (2012) |
| **Dipsadidae** | **Dipsadinae** | **Dipsadini** | ***"Geophis"*** | ***"Geophis" atlas*** | Found to be located within one of the clades of the paraphyletic "Geophis" in the phylogeny of X. However, this is most likely an artifact resulting from only including sequence data of a single mitochondrial gene for this species. No previous evidence supports this phylogenetic relationship. |
| Dipsadidae | Dipsadinae | Tribenov.2 | *Atractus* | *Atractus atratus* | Tribal classification following Sheehy (2012) |
| Dipsadidae | Dipsadinae | Tribenov.2 | *Atractus* | *Atractus attenuatus* | Tribal classification following Sheehy (2012) |
| Dipsadidae | Dipsadinae | Tribenov.2 | *Atractus* | *Atractus avernus* | Tribal classification following Sheehy (2012) |
| Dipsadidae | Dipsadinae | Tribenov.2 | *Atractus* | *Atractus ayeush* | Tribal classification following Sheehy (2012) |
| **Dipsadidae** | **Dipsadinae** | **Dipsadini** | ***"Geophis"*** | ***"Geophis" badius*** | Found to be located within one of the clades of the paraphyletic "Geophis" in the phylogeny of X. No previous evidence supports this phylogenetic relationship. |
| Dipsadidae | Dipsadinae | Tribenov.2 | *Atractus* | *Atractus biseriatus* | Tribal classification following Sheehy (2012) |
| Dipsadidae | Dipsadinae | Tribenov.2 | *Atractus* | *Atractus bocki* | Tribal classification following Sheehy (2012) |
| Dipsadidae | Dipsadinae | Tribenov.2 | *Atractus* | *Atractus bocourti* | Tribal classification following Sheehy (2012) |
| **Dipsadidae** | **Dipsadinae** | **Dipsadini** | ***"Geophis"*** | ***"Geophis" boimirim*** | Found to be located within one of the clades of the paraphyletic "Geophis" in the phylogeny of X. However, this is most likely an artifact resulting from only including sequence data of a single mitochondrial gene for this species. No previous evidence supports this phylogenetic relationship. |
| Dipsadidae | Dipsadinae | Tribenov.2 | *Atractus* | *Atractus boulengerii* | Tribal classification following Sheehy (2012) |
| Dipsadidae | Dipsadinae | Tribenov.2 | *Atractus* | *Atractus caete* | Tribal classification following Sheehy (2012) |
| Dipsadidae | Dipsadinae | Tribenov.2 | *Atractus* | *Atractus careolepis* | Tribal classification following Sheehy (2012) |
| Dipsadidae | Dipsadinae | Tribenov.2 | *Atractus* | *Atractus carrioni* | Tribal classification following Sheehy (2012) |
| Dipsadidae | Dipsadinae | Tribenov.2 | *Atractus* | *Atractus caxiuana* | Tribal classification following Sheehy (2012) |
| Dipsadidae | Dipsadinae | Tribenov.2 | *Atractus* | *Atractus cerberus* | Tribal classification following Sheehy (2012) |
| Dipsadidae | Dipsadinae | Tribenov.2 | *Atractus* | *Atractus charitoae* | Tribal classification following Sheehy (2012) |
| Dipsadidae | Dipsadinae | Tribenov.2 | *Atractus* | *Atractus chthonius* | Tribal classification following Sheehy (2012) |
| Dipsadidae | Dipsadinae | Tribenov.2 | *Atractus* | *Atractus clarki* | Tribal classification following Sheehy (2012) |
| Dipsadidae | Dipsadinae | Tribenov.2 | *Atractus* | *Atractus collaris* | Tribal classification following Sheehy (2012) |
| Dipsadidae | Dipsadinae | Tribenov.2 | *Atractus* | *Atractus crassicaudatus* | Tribal classification following Sheehy (2012) |
| Dipsadidae | Dipsadinae | Tribenov.2 | *Atractus* | *Atractus dapsilis* | Tribal classification following Sheehy (2012) |
| Dipsadidae | Dipsadinae | Tribenov.2 | *Atractus* | *Atractus darienensis* | Tribal classification following Sheehy (2012) |
| Dipsadidae | Dipsadinae | Tribenov.2 | *Atractus* | *Atractus depressiocellus* | Tribal classification following Sheehy (2012) |
| Dipsadidae | Dipsadinae | Tribenov.2 | *Atractus* | *Atractus discovery* | Tribal classification following Sheehy (2012) |
| Dipsadidae | Dipsadinae | Tribenov.2 | *Atractus* | *Atractus duboisi* | Tribal classification following Sheehy (2012) |
| Dipsadidae | Dipsadinae | Tribenov.2 | *Atractus* | *Atractus duidensis* | Tribal classification following Sheehy (2012) |
| Dipsadidae | Dipsadinae | Tribenov.2 | *Atractus* | *Atractus dunni* | Tribal classification following Sheehy (2012) |
| Dipsadidae | Dipsadinae | Tribenov.2 | *Atractus* | *Atractus echidna* | Tribal classification following Sheehy (2012) |
| Dipsadidae | Dipsadinae | Tribenov.2 | *Atractus* | *Atractus ecuadorensis* | Tribal classification following Sheehy (2012) |
| Dipsadidae | Dipsadinae | Tribenov.2 | *Atractus* | *Atractus edioi* | Tribal classification following Sheehy (2012) |
| Dipsadidae | Dipsadinae | Tribenov.2 | *Atractus* | *Atractus elaps* | Tribal classification following Sheehy (2012) |
| Dipsadidae | Dipsadinae | Tribenov.2 | *Atractus* | *Atractus emigdioi* | Tribal classification following Sheehy (2012) |
| Dipsadidae | Dipsadinae | Tribenov.2 | *Atractus* | *Atractus emmeli* | Tribal classification following Sheehy (2012) |
| Dipsadidae | Dipsadinae | Tribenov.2 | *Atractus* | *Atractus eriki* | Tribal classification following Sheehy (2012) |
| Dipsadidae | Dipsadinae | Tribenov.2 | *Atractus* | *Atractus erythromelas* | Tribal classification following Sheehy (2012) |
| Dipsadidae | Dipsadinae | Tribenov.2 | *Atractus* | *Atractus esepe* | Tribal classification following Sheehy (2012) |
| **Dipsadidae** | **Dipsadinae** | **Dipsadini** | ***"Geophis"*** | ***"Geophis" favae*** | Found to be located within one of the clades of the paraphyletic "Geophis" in the phylogeny of X. No previous evidence supports this phylogenetic relationship. |
| **Dipsadidae** | **Dipsadinae** | **Dipsadini** | ***"Geophis"*** | ***"Geophis" flammigerus*** | Found to be located within one of the clades of the paraphyletic "Geophis" in the phylogeny of X. No previous evidence supports this phylogenetic relationship. |
| Dipsadidae | Dipsadinae | Tribenov.2 | *Atractus* | *Atractus franciscopaivai* | Tribal classification following Sheehy (2012) |
| Dipsadidae | Dipsadinae | Tribenov.2 | *Atractus* | *Atractus francoi* | Tribal classification following Sheehy (2012) |
| Dipsadidae | Dipsadinae | Tribenov.2 | *Atractus* | *Atractus fuliginosus* | Tribal classification following Sheehy (2012) |
| Dipsadidae | Dipsadinae | Tribenov.2 | *Atractus* | *Atractus gaigeae* | Tribal classification following Sheehy (2012) |
| Dipsadidae | Dipsadinae | Tribenov.2 | *Atractus* | *Atractus gigas* | Tribal classification following Sheehy (2012) |
| Dipsadidae | Dipsadinae | Tribenov.2 | *Atractus* | *Atractus guentheri* | Tribal classification following Sheehy (2012) |
| Dipsadidae | Dipsadinae | Tribenov.2 | *Atractus* | *Atractus heliobelluomini* | Tribal classification following Sheehy (2012) |
| Dipsadidae | Dipsadinae | Tribenov.2 | *Atractus* | *Atractus heyeri* | Tribal classification following Sheehy (2012) |
| Dipsadidae | Dipsadinae | Tribenov.2 | *Atractus* | *Atractus hoogmoedi* | Tribal classification following Sheehy (2012) |
| Dipsadidae | Dipsadinae | Tribenov.2 | *Atractus* | *Atractus hostilitractus* | Tribal classification following Sheehy (2012) |
| **Dipsadidae** | **Dipsadinae** | **Dipsadini** | ***"Geophis"*** | ***"Geophis" imperfectus*** | Found to be located within one of the clades of the paraphyletic "Geophis" in the phylogeny of X. However, this is most likely an artifact resulting from only including sequence data of a single mitochondrial gene for this species. No previous evidence supports this phylogenetic relationship. |
| Dipsadidae | Dipsadinae | Tribenov.2 | *Atractus* | *Atractus indistinctus* | Tribal classification following Sheehy (2012) |
| Dipsadidae | Dipsadinae | Tribenov.2 | *Atractus* | *Atractus insipidus* | Tribal classification following Sheehy (2012) |
| Dipsadidae | Dipsadinae | Tribenov.2 | *Atractus* | *Atractus iridescens* | Tribal classification following Sheehy (2012) |
| Dipsadidae | Dipsadinae | Tribenov.2 | *Atractus* | *Atractus lancinii* | Tribal classification following Sheehy (2012) |
| Dipsadidae | Dipsadinae | Tribenov.2 | *Atractus* | *Atractus lasallei* | Tribal classification following Sheehy (2012) |
| Dipsadidae | Dipsadinae | Tribenov.2 | *Atractus* | *Atractus latifrons* | Tribal classification following Sheehy (2012) |
| Dipsadidae | Dipsadinae | Tribenov.2 | *Atractus* | *Atractus lehmanni* | Tribal classification following Sheehy (2012) |
| Dipsadidae | Dipsadinae | Tribenov.2 | *Atractus* | *Atractus loveridgei* | Tribal classification following Sheehy (2012) |
| Dipsadidae | Dipsadinae | Tribenov.2 | *Atractus* | *Atractus macondo* | Tribal classification following Sheehy (2012) |
| Dipsadidae | Dipsadinae | Tribenov.2 | *Atractus* | *Atractus maculatus* | Tribal classification following Sheehy (2012) |
| **Dipsadidae** | **Dipsadinae** | **Dipsadini** | ***"Geophis"*** | ***"Geophis" major*** | Found to be located within one of the clades of the paraphyletic "Geophis" in the phylogeny of X. No previous evidence supports this phylogenetic relationship. |
| Dipsadidae | Dipsadinae | Tribenov.2 | *Atractus* | *Atractus manizalesensis* | Tribal classification following Sheehy (2012) |
| Dipsadidae | Dipsadinae | Tribenov.2 | *Atractus* | *Atractus mariselae* | Tribal classification following Sheehy (2012) |
| Dipsadidae | Dipsadinae | Tribenov.2 | *Atractus* | *Atractus marthae* | Tribal classification following Sheehy (2012) |
| Dipsadidae | Dipsadinae | Tribenov.2 | *Atractus* | *Atractus matthewi* | Tribal classification following Sheehy (2012) |
| Dipsadidae | Dipsadinae | Tribenov.2 | *Atractus* | *Atractus medusa* | Tribal classification following Sheehy (2012) |
| Dipsadidae | Dipsadinae | Tribenov.2 | *Atractus* | *Atractus melanogaster* | Tribal classification following Sheehy (2012) |
| Dipsadidae | Dipsadinae | Tribenov.2 | *Atractus* | *Atractus melas* | Tribal classification following Sheehy (2012) |
| Dipsadidae | Dipsadinae | Tribenov.2 | *Atractus* | *Atractus meridensis* | Tribal classification following Sheehy (2012) |
| Dipsadidae | Dipsadinae | Tribenov.2 | *Atractus* | *Atractus michaelsabini* | Tribal classification following Sheehy (2012) |
| Dipsadidae | Dipsadinae | Tribenov.2 | *Atractus* | *Atractus micheleae* | Tribal classification following Sheehy (2012) |
| Dipsadidae | Dipsadinae | Tribenov.2 | *Atractus* | *Atractus microrhynchus* | Tribal classification following Sheehy (2012) |
| Dipsadidae | Dipsadinae | Tribenov.2 | *Atractus* | *Atractus mijaresi* | Tribal classification following Sheehy (2012) |
| Dipsadidae | Dipsadinae | Tribenov.2 | *Atractus* | *Atractus modestus* | Tribal classification following Sheehy (2012) |
| Dipsadidae | Dipsadinae | Tribenov.2 | *Atractus* | *Atractus multicinctus* | Tribal classification following Sheehy (2012) |
| Dipsadidae | Dipsadinae | Tribenov.2 | *Atractus* | *Atractus multidentatus* | Tribal classification following Sheehy (2012) |
| Dipsadidae | Dipsadinae | Tribenov.2 | *Atractus* | *Atractus nasutus* | Tribal classification following Sheehy (2012) |
| Dipsadidae | Dipsadinae | Tribenov.2 | *Atractus* | *Atractus natans* | Tribal classification following Sheehy (2012) |
| Dipsadidae | Dipsadinae | Tribenov.2 | *Atractus* | *Atractus nawa* | Tribal classification following Sheehy (2012) |
| Dipsadidae | Dipsadinae | Tribenov.2 | *Atractus* | *Atractus nicefori* | Tribal classification following Sheehy (2012) |
| Dipsadidae | Dipsadinae | Tribenov.2 | *Atractus* | *Atractus nigricauda* | Tribal classification following Sheehy (2012) |
| Dipsadidae | Dipsadinae | Tribenov.2 | *Atractus* | *Atractus nigriventris* | Tribal classification following Sheehy (2012) |
| Dipsadidae | Dipsadinae | Tribenov.2 | *Atractus* | *Atractus obesus* | Tribal classification following Sheehy (2012) |
| Dipsadidae | Dipsadinae | Tribenov.2 | *Atractus* | *Atractus obtusirostris* | Tribal classification following Sheehy (2012) |
| Dipsadidae | Dipsadinae | Tribenov.2 | *Atractus* | *Atractus occidentalis* | Tribal classification following Sheehy (2012) |
| Dipsadidae | Dipsadinae | Tribenov.2 | *Atractus* | *Atractus occipitoalbus* | Tribal classification following Sheehy (2012) |
| Dipsadidae | Dipsadinae | Tribenov.2 | *Atractus* | *Atractus ochrosetrus* | Tribal classification following Sheehy (2012) |
| Dipsadidae | Dipsadinae | Tribenov.2 | *Atractus* | *Atractus oculotemporalis* | Tribal classification following Sheehy (2012) |
| Dipsadidae | Dipsadinae | Tribenov.2 | *Atractus* | *Atractus orcesi* | Tribal classification following Sheehy (2012) |
| Dipsadidae | Dipsadinae | Tribenov.2 | *Atractus* | *Atractus pachacamac* | Tribal classification following Sheehy (2012) |
| Dipsadidae | Dipsadinae | Tribenov.2 | *Atractus* | *Atractus paisa* | Tribal classification following Sheehy (2012) |
| Dipsadidae | Dipsadinae | Tribenov.2 | *Atractus* | *Atractus pamplonensis* | Tribal classification following Sheehy (2012) |
| Dipsadidae | Dipsadinae | Tribenov.2 | *Atractus* | *Atractus pantostictus* | Tribal classification following Sheehy (2012) |
| Dipsadidae | Dipsadinae | Tribenov.2 | *Atractus* | *Atractus paraguayensis* | Tribal classification following Sheehy (2012) |
| Dipsadidae | Dipsadinae | Tribenov.2 | *Atractus* | *Atractus paucidens* | Tribal classification following Sheehy (2012) |
| Dipsadidae | Dipsadinae | Tribenov.2 | *Atractus* | *Atractus pauciscutatus* | Tribal classification following Sheehy (2012) |
| Dipsadidae | Dipsadinae | Tribenov.2 | *Atractus* | *Atractus peruvianus* | Tribal classification following Sheehy (2012) |
| Dipsadidae | Dipsadinae | Tribenov.2 | *Atractus* | *Atractus poeppigi* | Tribal classification following Sheehy (2012) |
| Dipsadidae | Dipsadinae | Tribenov.2 | *Atractus* | *Atractus potschi* | Tribal classification following Sheehy (2012) |
| Dipsadidae | Dipsadinae | Tribenov.2 | *Atractus* | *Atractus punctiventris* | Tribal classification following Sheehy (2012) |
| Dipsadidae | Dipsadinae | Tribenov.2 | *Atractus* | *Atractus resplendens* | Tribal classification following Sheehy (2012) |
| Dipsadidae | Dipsadinae | Tribenov.2 | *Atractus* | *Atractus reticulatus* | Tribal classification following Sheehy (2012) |
| Dipsadidae | Dipsadinae | Tribenov.2 | *Atractus* | *Atractus riveroi* | Tribal classification following Sheehy (2012) |
| Dipsadidae | Dipsadinae | Tribenov.2 | *Atractus* | *Atractus ronnie* | Tribal classification following Sheehy (2012) |
| Dipsadidae | Dipsadinae | Tribenov.2 | *Atractus* | *Atractus roulei* | Tribal classification following Sheehy (2012) |
| Dipsadidae | Dipsadinae | Tribenov.2 | *Atractus* | *Atractus sanctaemartae* | Tribal classification following Sheehy (2012) |
| Dipsadidae | Dipsadinae | Tribenov.2 | *Atractus* | *Atractus sanguineus* | Tribal classification following Sheehy (2012) |
| Dipsadidae | Dipsadinae | Tribenov.2 | *Atractus* | *Atractus savagei* | Tribal classification following Sheehy (2012) |
| Dipsadidae | Dipsadinae | Tribenov.2 | *Atractus* | *Atractus schach* | Tribal classification following Sheehy (2012) |
| Dipsadidae | Dipsadinae | Tribenov.2 | *Atractus* | *Atractus serranus* | Tribal classification following Sheehy (2012) |
| Dipsadidae | Dipsadinae | Tribenov.2 | *Atractus* | *Atractus snethlageae* | Tribal classification following Sheehy (2012) |
| Dipsadidae | Dipsadinae | Tribenov.2 | *Atractus* | *Atractus spinalis* | Tribal classification following Sheehy (2012) |
| Dipsadidae | Dipsadinae | Tribenov.2 | *Atractus* | *Atractus steyermarki* | Tribal classification following Sheehy (2012) |
| Dipsadidae | Dipsadinae | Tribenov.2 | *Atractus* | *Atractus stygius* | Tribal classification following Sheehy (2012) |
| Dipsadidae | Dipsadinae | Tribenov.2 | *Atractus* | *Atractus surucucu* | Tribal classification following Sheehy (2012) |
| Dipsadidae | Dipsadinae | Tribenov.2 | *Atractus* | *Atractus tamaensis* | Tribal classification following Sheehy (2012) |
| Dipsadidae | Dipsadinae | Tribenov.2 | *Atractus* | *Atractus tamessari* | Tribal classification following Sheehy (2012) |
| Dipsadidae | Dipsadinae | Tribenov.2 | *Atractus* | *Atractus taphorni* | Tribal classification following Sheehy (2012) |
| **Dipsadidae** | **Dipsadinae** | **Dipsadini** | ***"Geophis"*** | ***"Geophis" tartarus*** | Found to be located within one of the clades of the paraphyletic "Geophis" in the phylogeny of X. However, this is most likely an artifact resulting from only including sequence data of a single mitochondrial gene for this species. No previous evidence supports this phylogenetic relationship. |
| Dipsadidae | Dipsadinae | Tribenov.2 | *Atractus* | *Atractus thalesdelemai* | Tribal classification following Sheehy (2012) |
| Dipsadidae | Dipsadinae | Tribenov.2 | *Atractus* | *Atractus titanicus* | Tribal classification following Sheehy (2012) |
| **Dipsadidae** | **Dipsadinae** | **Tribenov.2** | ***"Geophis"*** | ***"Geophis" torquatus*** | Found to be located within one of the clades of the paraphyletic "Geophis" in the phylogeny of X. No previous evidence supports this phylogenetic relationship. |
| Dipsadidae | Dipsadinae | Tribenov.2 | *Atractus* | *Atractus touzeti* | Tribal classification following Sheehy (2012) |
| Dipsadidae | Dipsadinae | Tribenov.2 | *Atractus* | *Atractus trefauti* | Tribal classification following Sheehy (2012) |
| Dipsadidae | Dipsadinae | Tribenov.2 | *Atractus* | *Atractus trihedrurus* | Tribal classification following Sheehy (2012) |
| Dipsadidae | Dipsadinae | Tribenov.2 | *Atractus* | *Atractus trilineatus* | Tribal classification following Sheehy (2012) |
| Dipsadidae | Dipsadinae | Tribenov.2 | *Atractus* | *Atractus trivittatus* | Tribal classification following Sheehy (2012) |
| Dipsadidae | Dipsadinae | Tribenov.2 | *Atractus* | *Atractus turikensis* | Tribal classification following Sheehy (2012) |
| Dipsadidae | Dipsadinae | Tribenov.2 | *Atractus* | *Atractus typhon* | Tribal classification following Sheehy (2012) |
| Dipsadidae | Dipsadinae | Tribenov.2 | *Atractus* | *Atractus ukupacha* | Tribal classification following Sheehy (2012) |
| Dipsadidae | Dipsadinae | Tribenov.2 | *Atractus* | *Atractus variegatus* | Tribal classification following Sheehy (2012) |
| Dipsadidae | Dipsadinae | Tribenov.2 | *Atractus* | *Atractus ventrimaculatus* | Tribal classification following Sheehy (2012) |
| Dipsadidae | Dipsadinae | Tribenov.2 | *Atractus* | *Atractus vertebralis* | Tribal classification following Sheehy (2012) |
| Dipsadidae | Dipsadinae | Tribenov.2 | *Atractus* | *Atractus vertebrolineatus* | Tribal classification following Sheehy (2012) |
| Dipsadidae | Dipsadinae | Tribenov.2 | *Atractus* | *Atractus vittatus* | Tribal classification following Sheehy (2012) |
| Dipsadidae | Dipsadinae | Tribenov.2 | *Atractus* | *Atractus wagleri* | Tribal classification following Sheehy (2012) |
| Dipsadidae | Dipsadinae | Tribenov.2 | *Atractus* | *Atractus werneri* | Tribal classification following Sheehy (2012) |
| Dipsadidae | Dipsadinae | Tribenov.2 | *Atractus* | *Atractus zebrinus* | Tribal classification following Sheehy (2012) |
| Dipsadidae | Dipsadinae | Tribenov.2 | *Atractus* | *Atractus zgap* | Tribal classification following Sheehy (2012) |
| Dipsadidae | Dipsadinae | Tribenov.2 | *Atractus* | *Atractus zidoki* | Tribal classification following Sheehy (2012) |
| Dipsadidae | Xenodontinae | Eutrachelophiini | *Baliodryas* | *Baliodryas steinbachi* | Generic classification following Zaher & Prudente, 2019; Tribal classification following Moraes et al. (2021) |
| Dipsadidae | Xenodontinae | Pseudoboini | *Boiruna* | *Boiruna maculata* |  |
| Dipsadidae | Xenodontinae | Pseudoboini | *Boiruna* | *Boiruna sertaneja* |  |
| Dipsadidae | Xenodontinae | Alsophini | *Borikenophis* | *Borikenophis portoricensis* |  |
| Dipsadidae | Xenodontinae | Alsophini | *Borikenophis* | *Borikenophis prymnus* |  |
| Dipsadidae | Xenodontinae | Alsophini | *Borikenophis* | *Borikenophis sanctaecrucis* |  |
| Dipsadidae | Xenodontinae | Alsophini | *Borikenophis* | *Borikenophis variegatus* |  |
| Dipsadidae | Xenodontinae | Caaeteboini | *Caaeteboia* | *Caaeteboia amarali* |  |
| Dipsadidae | Xenodontinae | Caaeteboini | *Caaeteboia* | *Caaeteboia gaeli* |  |
| Dipsadidae | Xenodontinae | Tachymenini | *Calamodontophis* | *Calamodontophis paucidens* |  |
| Dipsadidae | Xenodontinae | Tachymenini | *Calamodontophis* | *Calamodontophis ronaldoi* |  |
| Dipsadidae | Xenodontinae | Alsophini | *Caraiba* | *Caraiba andreae* |  |
| Dipsadidae | Carphophiinae | - | *Carphophis* | *Carphophis amoenus* |  |
| Dipsadidae | Carphophiinae | - | *Carphophis* | *Carphophis vermis* |  |
| Dipsadidae | Dipsadinae | Insertae sedis in Dipsadinae1 | *Cenaspis* | *Cenaspis aenigma* |  |
| Dipsadidae | Insertae sedis in Dipsadidae1 | Insertae sedis family1 | *Cercophis* | *Cercophis auratus* |  |
| Dipsadidae | Dipsadinae | Tribenov.3 | *Chapinophis* | *Chapinophis xanthocheilus* | Tribal classification following Sheehy (2012) |
| Dipsadidae | Dipsadinae | Tribenov.4 | *Chersodromus* | *Chersodromus australis* |  |
| Dipsadidae | Dipsadinae | Tribenov.4 | *Chersodromus* | *Chersodromus liebmanni* |  |
| Dipsadidae | Dipsadinae | Tribenov.4 | *Chersodromus* | *Chersodromus nigrum* |  |
| Dipsadidae | Dipsadinae | Tribenov.4 | *Chersodromus* | *Chersodromus rubriventris* |  |
| Dipsadidae | Xenodontinae | Philodryadini | *Chlorosoma* | *Chlorosoma dunupyana* | Generic classification following Melo-Sampaio et al. (2020) |
| Dipsadidae | Xenodontinae | Philodryadini | *Chlorosoma* | *Chlorosoma laticeps* | Generic classification following Melo-Sampaio et al. (2020) |
| Dipsadidae | Xenodontinae | Philodryadini | *Chlorosoma* | *Chlorosoma viridissimum* | Generic classification following Melo-Sampaio et al. (2020) |
| **Dipsadidae** | **Xenodontinae** | **Xenodontini** | ***Clelia*** | ***Clelia clelia*** | For the TACT analysis we performed, we considered *Clelia* as part of the Xenodontini. We do so as X recovered the only species of *Clelia* sampled (=*Clelia clelia*) as part of this tribe. However, no other previous work has found evidence supporting this relationship or sequenced any of the remaining species of *Clelia*. Instead, previous studies recognize this species as part of the Pseudoboini |
| **Dipsadidae** | **Xenodontinae** | **Xenodontini** | ***Clelia*** | ***Clelia equatoriana*** | For the TACT analysis we performed, we considered *Clelia* as part of the Xenodontini. We do so as X recovered the only species of *Clelia* sampled (=*Clelia clelia*) as part of this tribe. However, no other previous work has found evidence supporting this relationship or sequenced any of the remaining species of *Clelia*. Instead, previous studies recognize this species as part of the Pseudoboini |
| **Dipsadidae** | **Xenodontinae** | **Xenodontini** | ***Clelia*** | ***Clelia errabunda*** | For the TACT analysis we performed, we considered *Clelia* as part of the Xenodontini. We do so as X recovered the only species of *Clelia* sampled (=*Clelia clelia*) as part of this tribe. However, no other previous work has found evidence supporting this relationship or sequenced any of the remaining species of *Clelia*. Instead, previous studies recognize this species as part of the Pseudoboini |
| **Dipsadidae** | **Xenodontinae** | **Xenodontini** | ***Clelia*** | ***Clelia hussami*** | For the TACT analysis we performed, we considered *Clelia* as part of the Xenodontini. We do so as X recovered the only species of *Clelia* sampled (=*Clelia clelia*) as part of this tribe. However, no other previous work has found evidence supporting this relationship or sequenced any of the remaining species of *Clelia*. Instead, previous studies recognize this species as part of the Pseudoboini |
| **Dipsadidae** | **Xenodontinae** | **Xenodontini** | ***Clelia*** | ***Clelia langeri*** | For the TACT analysis we performed, we considered *Clelia* as part of the Xenodontini. We do so as X recovered the only species of *Clelia* sampled (=*Clelia clelia*) as part of this tribe. However, no other previous work has found evidence supporting this relationship or sequenced any of the remaining species of *Clelia*. Instead, previous studies recognize this species as part of the Pseudoboini |
| **Dipsadidae** | **Xenodontinae** | **Xenodontini** | ***Clelia*** | ***Clelia plumbea*** | For the TACT analysis we performed, we considered *Clelia* as part of the Xenodontini. We do so as X recovered the only species of *Clelia* sampled (=*Clelia clelia*) as part of this tribe. However, no other previous work has found evidence supporting this relationship or sequenced any of the remaining species of *Clelia*. Instead, previous studies recognize this species as part of the Pseudoboini |
| **Dipsadidae** | **Xenodontinae** | **Xenodontini** | ***Clelia*** | ***Clelia scytalina*** | For the TACT analysis we performed, we considered *Clelia* as part of the Xenodontini. We do so as X recovered the only species of *Clelia* sampled (=*Clelia clelia*) as part of this tribe. However, no other previous work has found evidence supporting this relationship or sequenced any of the remaining species of *Clelia*. Instead, previous studies recognize this species as part of the Pseudoboini |
| Dipsadidae | Dipsadinae | Tribenov.3 | *Coniophanes* | *Coniophanes alvarezi* | Tribal classification following Sheehy (2012) |
| Dipsadidae | Dipsadinae | Tribenov.3 | *Coniophanes* | *Coniophanes andresensis* | Tribal classification following Sheehy (2012) |
| Dipsadidae | Dipsadinae | Tribenov.3 | *Coniophanes* | *Coniophanes bipunctatus* | Tribal classification following Sheehy (2012) |
| Dipsadidae | Dipsadinae | Tribenov.3 | *Coniophanes* | *Coniophanes dromiciformis* | Tribal classification following Sheehy (2012) |
| Dipsadidae | Dipsadinae | Tribenov.3 | *Coniophanes* | *Coniophanes fissidens* | Tribal classification following Sheehy (2012) |
| Dipsadidae | Dipsadinae | Tribenov.3 | *Coniophanes* | *Coniophanes imperialis* | Tribal classification following Sheehy (2012) |
| Dipsadidae | Dipsadinae | Tribenov.3 | *Coniophanes* | *Coniophanes joanae* | Tribal classification following Sheehy (2012) |
| Dipsadidae | Dipsadinae | Tribenov.3 | *Coniophanes* | *Coniophanes lateritius* | Tribal classification following Sheehy (2012) |
| Dipsadidae | Dipsadinae | Tribenov.3 | *Coniophanes* | *Coniophanes longinquus* | Tribal classification following Sheehy (2012) |
| Dipsadidae | Dipsadinae | Tribenov.3 | *Coniophanes* | *Coniophanes melanocephalus* | Tribal classification following Sheehy (2012) |
| Dipsadidae | Dipsadinae | Tribenov.3 | *Coniophanes* | *Coniophanes meridanus* | Tribal classification following Sheehy (2012) |
| Dipsadidae | Dipsadinae | Tribenov.3 | *Coniophanes* | *Coniophanes michoacanensis* | Tribal classification following Sheehy (2012) |
| Dipsadidae | Dipsadinae | Tribenov.3 | *Coniophanes* | *Coniophanes piceivittis* | Tribal classification following Sheehy (2012) |
| Dipsadidae | Dipsadinae | Tribenov.3 | *Coniophanes* | *Coniophanes quinquevittatus* | Tribal classification following Sheehy (2012) |
| Dipsadidae | Dipsadinae | Tribenov.3 | *Coniophanes* | *Coniophanes schmidti* | Tribal classification following Sheehy (2012) |
| Dipsadidae | Dipsadinae | Tribenov.3 | *Coniophanes* | *Coniophanes taeniata* | Tribal classification following Sheehy (2012) |
| Dipsadidae | Dipsadinae | Tribenov.3 | *Coniophanes* | *Coniophanes taylori* | Tribal classification following Sheehy (2012) |
| Dipsadidae | Xenodontinae | Conophini | *Conophis* | *Conophis lineatus* |  |
| Dipsadidae | Xenodontinae | Conophini | *Conophis* | *Conophis morai* |  |
| Dipsadidae | Xenodontinae | Conophini | *Conophis* | *Conophis vittatus* |  |
| Dipsadidae | Carphophiinae | - | *Contia* | *Contia longicaudae* |  |
| Dipsadidae | Carphophiinae | - | *Contia* | *Contia tenuis* |  |
| Dipsadidae | Xenodontinae | Elapomorphini | *Coronelaps* | *Coronelaps lepidus* |  |
| Dipsadidae | Xenodontinae | Insertae sedis in Xenodontinae1 | *Crisantophis* | *Crisantophis nevermanni* |  |
| Dipsadidae | Dipsadinae | Tribenov.1 | *Cryophis* | *Cryophis hallbergi* | Tribal classification following Sheehy (2012) |
| Dipsadidae | Xenodontinae | Alsophini | *Cubophis* | *Cubophis brooksi* |  |
| Dipsadidae | Xenodontinae | Alsophini | *Cubophis* | *Cubophis cantherigerus* |  |
| Dipsadidae | Xenodontinae | Alsophini | *Cubophis* | *Cubophis caymanus* |  |
| Dipsadidae | Xenodontinae | Alsophini | *Cubophis* | *Cubophis fuscicauda* |  |
| Dipsadidae | Xenodontinae | Alsophini | *Cubophis* | *Cubophis ruttyi* |  |
| Dipsadidae | Xenodontinae | Alsophini | *Cubophis* | *Cubophis vudii* |  |
| Dipsadidae | Carphophiinae | - | *Diadophis* | *Diadophis punctatus* |  |
| Dipsadidae | Dipsadinae | Diaphorolepini | *Diaphorolepis* | *Diaphorolepis laevis* |  |
| Dipsadidae | Dipsadinae | Diaphorolepini | *Diaphorolepis* | *Diaphorolepis wagneri* |  |
| Dipsadidae | Xenodontinae | Echinantherini | *Dibernardia* | *Dibernardia affinis* | Generic classification following Abegg et al. (2022) |
| Dipsadidae | Xenodontinae | Echinantherini | *Dibernardia* | *Dibernardia bilineata* | Generic classification following Abegg et al. (2022) |
| Dipsadidae | Xenodontinae | Echinantherini | *Dibernardia* | *Dibernardia persimilis* | Generic classification following Abegg et al. (2022) |
| Dipsadidae | Xenodontinae | Echinantherini | *Dibernardia* | *Dibernardia poecilopogon* | Generic classification following Abegg et al. (2022) |
| Dipsadidae | Dipsadinae | Dipsadini | *Dipsas* | *Dipsas albifrons* |  |
| Dipsadidae | Dipsadinae | Dipsadini | *Dipsas* | *Dipsas alternans* |  |
| Dipsadidae | Dipsadinae | Dipsadini | *Dipsas* | *Dipsas andiana* |  |
| Dipsadidae | Dipsadinae | Dipsadini | *Dipsas* | *Dipsas articulata* |  |
| Dipsadidae | Dipsadinae | Dipsadini | *Dipsas* | *Dipsas baliomelas* |  |
| Dipsadidae | Dipsadinae | Dipsadini | *Dipsas* | *Dipsas bicolor* |  |
| Dipsadidae | Dipsadinae | Dipsadini | *Dipsas* | *Dipsas bobridgelyi* |  |
| Dipsadidae | Dipsadinae | Dipsadini | *Dipsas* | *Dipsas bothropoides* |  |
| Dipsadidae | Dipsadinae | Dipsadini | *Dipsas* | *Dipsas brevifacies* |  |
| Dipsadidae | Dipsadinae | Dipsadini | *Dipsas* | *Dipsas bucephala* |  |
| Dipsadidae | Dipsadinae | Dipsadini | *Dipsas* | *Dipsas catesbyi* |  |
| Dipsadidae | Dipsadinae | Dipsadini | *Dipsas* | *Dipsas chaparensis* |  |
| Dipsadidae | Dipsadinae | Dipsadini | *Dipsas* | *Dipsas cisticeps* |  |
| Dipsadidae | Dipsadinae | Dipsadini | *Dipsas* | *Dipsas copei* |  |
| Dipsadidae | Dipsadinae | Dipsadini | *Dipsas* | *Dipsas elegans* |  |
| Dipsadidae | Dipsadinae | Dipsadini | *Dipsas* | *Dipsas ellipsifera* |  |
| Dipsadidae | Dipsadinae | Dipsadini | *Dipsas* | *Dipsas gaigeae* |  |
| Dipsadidae | Dipsadinae | Dipsadini | *Dipsas* | *Dipsas georgejetti* |  |
| Dipsadidae | Dipsadinae | Dipsadini | *Dipsas* | *Dipsas gracilis* |  |
| Dipsadidae | Dipsadinae | Dipsadini | *Dipsas* | *Dipsas incerta* |  |
| Dipsadidae | Dipsadinae | Dipsadini | *Dipsas* | *Dipsas indica* |  |
| Dipsadidae | Dipsadinae | Dipsadini | *Dipsas* | *Dipsas jamespetersi* |  |
| Dipsadidae | Dipsadinae | Dipsadini | *Dipsas* | *Dipsas klebbai* |  |
| Dipsadidae | Dipsadinae | Dipsadini | *Dipsas* | *Dipsas latifrontalis* |  |
| Dipsadidae | Dipsadinae | Dipsadini | *Dipsas* | *Dipsas lavillai* |  |
| Dipsadidae | Dipsadinae | Dipsadini | *Dipsas* | *Dipsas maxillaris* |  |
| Dipsadidae | Dipsadinae | Dipsadini | *Dipsas* | *Dipsas mikanii* |  |
| Dipsadidae | Dipsadinae | Dipsadini | *Dipsas* | *Dipsas neuwiedi* |  |
| Dipsadidae | Dipsadinae | Dipsadini | *Dipsas* | *Dipsas nicholsi* |  |
| Dipsadidae | Dipsadinae | Dipsadini | *Dipsas* | *Dipsas oligozonata* |  |
| Dipsadidae | Dipsadinae | Dipsadini | *Dipsas* | *Dipsas oneilli* |  |
| Dipsadidae | Dipsadinae | Dipsadini | *Dipsas* | *Dipsas oreas* |  |
| Dipsadidae | Dipsadinae | Dipsadini | *Dipsas* | *Dipsas oswaldobaezi* |  |
| Dipsadidae | Dipsadinae | Dipsadini | *Dipsas* | *Dipsas pakaraima* |  |
| Dipsadidae | Dipsadinae | Dipsadini | *Dipsas* | *Dipsas palmeri* |  |
| Dipsadidae | Dipsadinae | Dipsadini | *Dipsas* | *Dipsas pavonina* |  |
| Dipsadidae | Dipsadinae | Dipsadini | *Dipsas* | *Dipsas peruana* |  |
| Dipsadidae | Dipsadinae | Dipsadini | *Dipsas* | *Dipsas praeornata* |  |
| Dipsadidae | Dipsadinae | Dipsadini | *Dipsas* | *Dipsas pratti* |  |
| Dipsadidae | Dipsadinae | Dipsadini | *Dipsas* | *Dipsas sanctijoannis* |  |
| Dipsadidae | Dipsadinae | Dipsadini | *Dipsas* | *Dipsas sazimai* |  |
| Dipsadidae | Dipsadinae | Dipsadini | *Dipsas* | *Dipsas schunkii* |  |
| Dipsadidae | Dipsadinae | Dipsadini | *Dipsas* | *Dipsas temporalis* |  |
| Dipsadidae | Dipsadinae | Dipsadini | *Dipsas* | *Dipsas tenuissima* |  |
| Dipsadidae | Dipsadinae | Dipsadini | *Dipsas* | *Dipsas trinitatis* |  |
| Dipsadidae | Dipsadinae | Dipsadini | *Dipsas* | *Dipsas turgida* |  |
| Dipsadidae | Dipsadinae | Dipsadini | *Dipsas* | *Dipsas vagrans* |  |
| Dipsadidae | Dipsadinae | Dipsadini | *Dipsas* | *Dipsas vagus* |  |
| Dipsadidae | Dipsadinae | Dipsadini | *Dipsas* | *Dipsas variegata* |  |
| Dipsadidae | Dipsadinae | Dipsadini | *Dipsas* | *Dipsas ventrimaculata* |  |
| Dipsadidae | Dipsadinae | Dipsadini | *Dipsas* | *Dipsas vermiculata* |  |
| Dipsadidae | Dipsadinae | Dipsadini | *Dipsas* | *Dipsas viguieri* |  |
| Dipsadidae | Dipsadinae | Dipsadini | *Dipsas* | *Dipsas williamsi* |  |
| Dipsadidae | Xenodontinae | Philodryadini | *Ditaxodon* | *Ditaxodon taeniatus* |  |
| Dipsadidae | Xenodontinae | Pseudoboini | *Drepanoides* | *Drepanoides anomalus* |  |
| Dipsadidae | Xenodontinae | Tachymenini | *Dryophylax* | *Dryophylax almae* | Generic classification following Trevine et al. (2022) |
| Dipsadidae | Xenodontinae | Tachymenini | *Dryophylax* | *Dryophylax ceibae* | Generic classification following Trevine et al. (2022) |
| Dipsadidae | Xenodontinae | Tachymenini | *Dryophylax* | *Dryophylax chaquensis* | Generic classification following Trevine et al. (2022) |
| Dipsadidae | Xenodontinae | Tachymenini | *Dryophylax* | *Dryophylax chimanta* | Generic classification following Trevine et al. (2022) |
| Dipsadidae | Xenodontinae | Tachymenini | *Dryophylax* | *Dryophylax corocoroensis* | Generic classification following Trevine et al. (2022) |
| Dipsadidae | Xenodontinae | Tachymenini | *Dryophylax* | *Dryophylax dixoni* | Generic classification following Trevine et al. (2022) |
| Dipsadidae | Xenodontinae | Tachymenini | *Dryophylax* | *Dryophylax duida* | Generic classification following Trevine et al. (2022) |
| Dipsadidae | Xenodontinae | Tachymenini | *Dryophylax* | *Dryophylax gambotensis* | Generic classification following Trevine et al. (2022) |
| Dipsadidae | Xenodontinae | Tachymenini | *Dryophylax* | *Dryophylax hypoconia* | Generic classification following Trevine et al. (2022) |
| Dipsadidae | Xenodontinae | Tachymenini | *Dryophylax* | *Dryophylax marahuaquensis* | Generic classification following Trevine et al. (2022) |
| Dipsadidae | Xenodontinae | Tachymenini | *Dryophylax* | *Dryophylax nattereri* | Generic classification following Trevine et al. (2022) |
| Dipsadidae | Xenodontinae | Tachymenini | *Dryophylax* | *Dryophylax paraguanae* | Generic classification following Trevine et al. (2022) |
| Dipsadidae | Xenodontinae | Tachymenini | *Dryophylax* | *Dryophylax phoenix* | Generic classification following Trevine et al. (2022) |
| Dipsadidae | Xenodontinae | Tachymenini | *Dryophylax* | *Dryophylax ramonriveroi* | Generic classification following Trevine et al. (2022) |
| Dipsadidae | Xenodontinae | Tachymenini | *Dryophylax* | *Dryophylax yavi* | Generic classification following Trevine et al. (2022) |
| Dipsadidae | Xenodontinae | Echinantherini | *Echinanthera* | *Echinanthera cephalomaculata* |  |
| Dipsadidae | Xenodontinae | Echinantherini | *Echinanthera* | *Echinanthera cephalostriata* |  |
| Dipsadidae | Xenodontinae | Echinantherini | *Echinanthera* | *Echinanthera cyanopleura* |  |
| Dipsadidae | Xenodontinae | Echinantherini | *Echinanthera* | *Echinanthera melanostigma* |  |
| Dipsadidae | Xenodontinae | Echinantherini | *Echinanthera* | *Echinanthera undulata* |  |
| Dipsadidae | Xenodontinae | Elapomorphini | *Elapomorphus* | *Elapomorphus quinquelineatus* |  |
| Dipsadidae | Xenodontinae | Elapomorphini | *Elapomorphus* | *Elapomorphus wuchereri* |  |
| Dipsadidae | Dipsadinae | Diaphorolepini | *Emmochliophis* | *Emmochliophis fugleri* |  |
| Dipsadidae | Dipsadinae | Diaphorolepini | *Emmochliophis* | *Emmochliophis miops* |  |
| Dipsadidae | Dipsadinae | Tribenov.5 | *Enuliophis* | *Enuliophis sclateri* | Tribal classification following Sheehy (2012) |
| Dipsadidae | Dipsadinae | Tribenov.5 | *Enulius* | *Enulius bifoveatus* | Tribal classification following Sheehy (2012) |
| Dipsadidae | Dipsadinae | Tribenov.5 | *Enulius* | *Enulius flavitorques* | Tribal classification following Sheehy (2012) |
| Dipsadidae | Dipsadinae | Tribenov.5 | *Enulius* | *Enulius oligostichus* | Tribal classification following Sheehy (2012) |
| Dipsadidae | Dipsadinae | Tribenov.5 | *Enulius* | *Enulius roatanensis* | Tribal classification following Sheehy (2012) |
| Dipsadidae | Xenodontinae | Xenodontini | *Erythrolamprus* | *Erythrolamprus aenigma* |  |
| Dipsadidae | Xenodontinae | Xenodontini | *Erythrolamprus* | *Erythrolamprus aesculapii* |  |
| Dipsadidae | Xenodontinae | Xenodontini | *Erythrolamprus* | *Erythrolamprus albertguentheri* |  |
| Dipsadidae | Xenodontinae | Xenodontini | *Erythrolamprus* | *Erythrolamprus albiventris* |  |
| Dipsadidae | Xenodontinae | Xenodontini | *Erythrolamprus* | *Erythrolamprus almadensis* |  |
| Dipsadidae | Xenodontinae | Xenodontini | *Erythrolamprus* | *Erythrolamprus andinus* |  |
| Dipsadidae | Xenodontinae | Xenodontini | *Erythrolamprus* | *Erythrolamprus atraventer* |  |
| Dipsadidae | Xenodontinae | Xenodontini | *Erythrolamprus* | *Erythrolamprus bizona* |  |
| Dipsadidae | Xenodontinae | Xenodontini | *Erythrolamprus* | *Erythrolamprus breviceps* |  |
| Dipsadidae | Xenodontinae | Xenodontini | *Erythrolamprus* | *Erythrolamprus carajasensis* |  |
| Dipsadidae | Xenodontinae | Xenodontini | *Erythrolamprus* | *Erythrolamprus ceii* |  |
| Dipsadidae | Xenodontinae | Xenodontini | *Erythrolamprus* | *Erythrolamprus cobella* |  |
| Dipsadidae | Xenodontinae | Xenodontini | *Erythrolamprus* | *Erythrolamprus cursor* |  |
| Dipsadidae | Xenodontinae | Xenodontini | *Erythrolamprus* | *Erythrolamprus dorsocorallinus* |  |
| Dipsadidae | Xenodontinae | Xenodontini | *Erythrolamprus* | *Erythrolamprus epinephalus* |  |
| Dipsadidae | Xenodontinae | Xenodontini | *Erythrolamprus* | *Erythrolamprus festae* |  |
| Dipsadidae | Xenodontinae | Xenodontini | *Erythrolamprus* | *Erythrolamprus fraseri* |  |
| Dipsadidae | Xenodontinae | Xenodontini | *Erythrolamprus* | *Erythrolamprus frenatus* |  |
| Dipsadidae | Xenodontinae | Xenodontini | *Erythrolamprus* | *Erythrolamprus guentheri* |  |
| Dipsadidae | Xenodontinae | Xenodontini | *Erythrolamprus* | *Erythrolamprus ingeri* |  |
| Dipsadidae | Xenodontinae | Xenodontini | *Erythrolamprus* | *Erythrolamprus jaegeri* |  |
| Dipsadidae | Xenodontinae | Xenodontini | *Erythrolamprus* | *Erythrolamprus janaleeae* |  |
| Dipsadidae | Xenodontinae | Xenodontini | *Erythrolamprus* | *Erythrolamprus juliae* |  |
| Dipsadidae | Xenodontinae | Xenodontini | *Erythrolamprus* | *Erythrolamprus lamonae* |  |
| Dipsadidae | Xenodontinae | Xenodontini | *Erythrolamprus* | *Erythrolamprus macrosomus* |  |
| Dipsadidae | Xenodontinae | Xenodontini | *Erythrolamprus* | *Erythrolamprus maryellenae* |  |
| Dipsadidae | Xenodontinae | Xenodontini | *Erythrolamprus* | *Erythrolamprus melanotus* |  |
| Dipsadidae | Xenodontinae | Xenodontini | *Erythrolamprus* | *Erythrolamprus mertensi* |  |
| Dipsadidae | Xenodontinae | Xenodontini | *Erythrolamprus* | *Erythrolamprus miliaris* |  |
| Dipsadidae | Xenodontinae | Xenodontini | *Erythrolamprus* | *Erythrolamprus mimus* |  |
| Dipsadidae | Xenodontinae | Xenodontini | *Erythrolamprus* | *Erythrolamprus mossoroensis* |  |
| Dipsadidae | Xenodontinae | Xenodontini | *Erythrolamprus* | *Erythrolamprus ocellatus* |  |
| Dipsadidae | Xenodontinae | Xenodontini | *Erythrolamprus* | *Erythrolamprus oligolepis* |  |
| Dipsadidae | Xenodontinae | Xenodontini | *Erythrolamprus* | *Erythrolamprus ornatus* |  |
| Dipsadidae | Xenodontinae | Xenodontini | *Erythrolamprus* | *Erythrolamprus perfuscus* |  |
| Dipsadidae | Xenodontinae | Xenodontini | *Erythrolamprus* | *Erythrolamprus poecilogyrus* |  |
| Dipsadidae | Xenodontinae | Xenodontini | *Erythrolamprus* | *Erythrolamprus pseudocorallus* |  |
| Dipsadidae | Xenodontinae | Xenodontini | *Erythrolamprus* | *Erythrolamprus pseudoreginae* |  |
| Dipsadidae | Xenodontinae | Xenodontini | *Erythrolamprus* | *Erythrolamprus pyburni* |  |
| Dipsadidae | Xenodontinae | Xenodontini | *Erythrolamprus* | *Erythrolamprus pygmaeus* |  |
| Dipsadidae | Xenodontinae | Xenodontini | *Erythrolamprus* | *Erythrolamprus reginae* |  |
| Dipsadidae | Xenodontinae | Xenodontini | *Erythrolamprus* | *Erythrolamprus rochai* |  |
| **Dipsadidae** | **Xenodontinae** | **Xenodontini** | ***Lygophis*** | ***Lygophis sagittifer*** | Found to be located within the otherwise monophyletic genus *Lygophis* in the phylogeny of X. No previous evidence supports this phylogenetic relationship. |
| Dipsadidae | Xenodontinae | Xenodontini | *Erythrolamprus* | *Erythrolamprus semiaureus* |  |
| Dipsadidae | Xenodontinae | Xenodontini | *Erythrolamprus* | *Erythrolamprus subocularis* |  |
| Dipsadidae | Xenodontinae | Xenodontini | *Erythrolamprus* | *Erythrolamprus taeniogaster* |  |
| Dipsadidae | Xenodontinae | Xenodontini | *Erythrolamprus* | *Erythrolamprus taeniurus* |  |
| Dipsadidae | Xenodontinae | Xenodontini | *Erythrolamprus* | *Erythrolamprus torrenicola* |  |
| Dipsadidae | Xenodontinae | Xenodontini | *Erythrolamprus* | *Erythrolamprus trebbaui* |  |
| Dipsadidae | Xenodontinae | Xenodontini | *Erythrolamprus* | *Erythrolamprus triscalis* |  |
| Dipsadidae | Xenodontinae | Xenodontini | *Erythrolamprus* | *Erythrolamprus typhlus* |  |
| Dipsadidae | Xenodontinae | Xenodontini | *Erythrolamprus* | *Erythrolamprus viridis* |  |
| Dipsadidae | Xenodontinae | Xenodontini | *Erythrolamprus* | *Erythrolamprus vitti* |  |
| Dipsadidae | Xenodontinae | Xenodontini | *Erythrolamprus* | *Erythrolamprus williamsi* |  |
| Dipsadidae | Xenodontinae | Xenodontini | *Erythrolamprus* | *Erythrolamprus zweifeli* |  |
| Dipsadidae | Xenodontinae | Eutrachelophiini | *Eutrachelophis* | *Eutrachelophis bassleri* | Tribal classification following Moraes et al. (2021) |
| Dipsadidae | Xenodontinae | Eutrachelophiini | *Eutrachelophis* | *Eutrachelophis papilio* | Tribal classification following Moraes et al. (2021) |
| Dipsadidae | Carphophiinae | - | *Farancia* | *Farancia abacura* |  |
| Dipsadidae | Carphophiinae | - | *Farancia* | *Farancia erytrogramma* |  |
| Dipsadidae | Xenodontinae | Tachymenini | *Galvarinus* | *Galvarinus attenuatus* | Generic classification following Trevine et al. (2022) |
| Dipsadidae | Xenodontinae | Tachymenini | *Galvarinus* | *Galvarinus chilensis* | Generic classification following Trevine et al. (2022) |
| Dipsadidae | Xenodontinae | Tachymenini | *Galvarinus* | *Galvarinus tarmensis* | Generic classification following Trevine et al. (2022) |
| Dipsadidae | Dipsadinae | Dipsadini | *Geophis* | *Geophis annuliferus* |  |
| Dipsadidae | Dipsadinae | Dipsadini | *Geophis* | *Geophis anocularis* |  |
| Dipsadidae | Dipsadinae | Dipsadini | *Geophis* | *Geophis bellus* |  |
| Dipsadidae | Dipsadinae | Dipsadini | *Geophis* | *Geophis betaniensis* |  |
| Dipsadidae | Dipsadinae | Dipsadini | *Geophis* | *Geophis bicolor* |  |
| Dipsadidae | Dipsadinae | Dipsadini | *Geophis* | *Geophis blanchardi* |  |
| Dipsadidae | Dipsadinae | Dipsadini | *Geophis* | *Geophis brachycephalus* |  |
| Dipsadidae | Dipsadinae | Dipsadini | *Geophis* | *Geophis cancellatus* |  |
| Dipsadidae | Dipsadinae | Dipsadini | *Geophis* | *Geophis cansecoi* |  |
| Dipsadidae | Dipsadinae | Dipsadini | *Geophis* | *Geophis carinosus* |  |
| Dipsadidae | Dipsadinae | Dipsadini | *Geophis* | *Geophis chalybeus* |  |
| Dipsadidae | Dipsadinae | Dipsadini | *Geophis* | *Geophis championi* |  |
| Dipsadidae | Dipsadinae | Dipsadini | *Geophis* | *Geophis damiani* |  |
| Dipsadidae | Dipsadinae | Dipsadini | *Geophis* | *Geophis downsi* |  |
| Dipsadidae | Dipsadinae | Dipsadini | *Geophis* | *Geophis dubius* |  |
| Dipsadidae | Dipsadinae | Dipsadini | *Geophis* | *Geophis duellmani* |  |
| Dipsadidae | Dipsadinae | Dipsadini | *Geophis* | *Geophis dugesii* |  |
| Dipsadidae | Dipsadinae | Dipsadini | *Geophis* | *Geophis dunni* |  |
| Dipsadidae | Dipsadinae | Dipsadini | *Geophis* | *Geophis fulvoguttatus* |  |
| Dipsadidae | Dipsadinae | Dipsadini | *Geophis* | *Geophis godmani* |  |
| Dipsadidae | Dipsadinae | Dipsadini | *Geophis* | *Geophis hoffmanni* |  |
| Dipsadidae | Dipsadinae | Dipsadini | *Geophis* | *Geophis immaculatus* |  |
| Dipsadidae | Dipsadinae | Dipsadini | *Geophis* | *Geophis incomptus* |  |
| Dipsadidae | Dipsadinae | Dipsadini | *Geophis* | *Geophis isthmicus* |  |
| Dipsadidae | Dipsadinae | Dipsadini | *Geophis* | *Geophis juarezi* |  |
| Dipsadidae | Dipsadinae | Dipsadini | *Geophis* | *Geophis juliai* |  |
| Dipsadidae | Dipsadinae | Dipsadini | *Geophis* | *Geophis laticinctus* |  |
| Dipsadidae | Dipsadinae | Dipsadini | *Geophis* | *Geophis laticollaris* |  |
| Dipsadidae | Dipsadinae | Dipsadini | *Geophis* | *Geophis latifrontalis* |  |
| Dipsadidae | Dipsadinae | Dipsadini | *Geophis* | *Geophis lorancai* |  |
| Dipsadidae | Dipsadinae | Dipsadini | *Geophis* | *Geophis maculiferus* |  |
| Dipsadidae | Dipsadinae | Dipsadini | *Geophis* | *Geophis mutitorques* |  |
| Dipsadidae | Dipsadinae | Dipsadini | *Geophis* | *Geophis nasalis* |  |
| Dipsadidae | Dipsadinae | Dipsadini | *Geophis* | *Geophis nephodrymus* |  |
| Dipsadidae | Dipsadinae | Dipsadini | *Geophis* | *Geophis nigroalbus* |  |
| Dipsadidae | Dipsadinae | Dipsadini | *Geophis* | *Geophis nigrocinctus* |  |
| Dipsadidae | Dipsadinae | Dipsadini | *Geophis* | *Geophis occabus* |  |
| Dipsadidae | Dipsadinae | Dipsadini | *Geophis* | *Geophis omiltemanus* |  |
| Dipsadidae | Dipsadinae | Dipsadini | *Geophis* | *Geophis petersii* |  |
| Dipsadidae | Dipsadinae | Dipsadini | *Geophis* | *Geophis pyburni* |  |
| **Dipsadidae** | **Dipsadinae** | **Tribenov.2** | ***"Atractus"*** | ***"Atractus" rhodogaster*** | Found to be located within the genus *Atractus* in the phylogeny of X. However, this is most likely an artifact resulting from only including sequence data of a single mitochondrial gene for this species. No previous evidence supports this phylogenetic relationship. |
| Dipsadidae | Dipsadinae | Dipsadini | *Geophis* | *Geophis rostralis* |  |
| Dipsadidae | Dipsadinae | Dipsadini | *Geophis* | *Geophis ruthveni* |  |
| Dipsadidae | Dipsadinae | Dipsadini | *Geophis* | *Geophis sallaei* |  |
| Dipsadidae | Dipsadinae | Dipsadini | *Geophis* | *Geophis sanniolus* |  |
| Dipsadidae | Dipsadinae | Dipsadini | *Geophis* | *Geophis sartorii* |  |
| Dipsadidae | Dipsadinae | Dipsadini | *Geophis* | *Geophis semidoliatus* |  |
| Dipsadidae | Dipsadinae | Dipsadini | *Geophis* | *Geophis sieboldi* |  |
| Dipsadidae | Dipsadinae | Dipsadini | *Geophis* | *Geophis talamancae* |  |
| Dipsadidae | Dipsadinae | Dipsadini | *Geophis* | *Geophis tarascae* |  |
| Dipsadidae | Dipsadinae | Dipsadini | *Geophis* | *Geophis tectus* |  |
| Dipsadidae | Dipsadinae | Dipsadini | *Geophis* | *Geophis turbidus* |  |
| Dipsadidae | Dipsadinae | Dipsadini | *Geophis* | *Geophis zeledoni* |  |
| Dipsadidae | Xenodontinae | Tachymenini | *Gomesophis* | *Gomesophis brasiliensis* |  |
| Dipsadidae | Xenodontinae | Alsophini | *Haitiophis* | *Haitiophis anomalus* |  |
| Dipsadidae | Xenodontinae | Hydropsini | *Helicops* | *Helicops acangussu* |  |
| Dipsadidae | Xenodontinae | Hydropsini | *Helicops* | *Helicops angulatus* |  |
| Dipsadidae | Xenodontinae | Hydropsini | *Helicops* | *Helicops apiaka* |  |
| Dipsadidae | Xenodontinae | Hydropsini | *Helicops* | *Helicops boitata* |  |
| Dipsadidae | Xenodontinae | Hydropsini | *Helicops* | *Helicops carinicaudus* |  |
| Dipsadidae | Xenodontinae | Hydropsini | *Helicops* | *Helicops danieli* |  |
| Dipsadidae | Xenodontinae | Hydropsini | *Helicops* | *Helicops gomesi* |  |
| Dipsadidae | Xenodontinae | Hydropsini | *Helicops* | *Helicops hagmanni* |  |
| Dipsadidae | Xenodontinae | Hydropsini | *Helicops* | *Helicops infrataeniatus* |  |
| Dipsadidae | Xenodontinae | Hydropsini | *Helicops* | *Helicops leopardinus* |  |
| Dipsadidae | Xenodontinae | Hydropsini | *Helicops* | *Helicops modestus* |  |
| Dipsadidae | Xenodontinae | Hydropsini | *Helicops* | *Helicops nentur* |  |
| Dipsadidae | Xenodontinae | Hydropsini | *Helicops* | *Helicops pastazae* |  |
| Dipsadidae | Xenodontinae | Hydropsini | *Helicops* | *Helicops petersi* |  |
| Dipsadidae | Xenodontinae | Hydropsini | *Helicops* | *Helicops phantasma* |  |
| Dipsadidae | Xenodontinae | Hydropsini | *Helicops* | *Helicops polylepis* |  |
| Dipsadidae | Xenodontinae | Hydropsini | *Helicops* | *Helicops scalaris* |  |
| Dipsadidae | Xenodontinae | Hydropsini | *Helicops* | *Helicops tapajonicus* |  |
| Dipsadidae | Xenodontinae | Hydropsini | *Helicops* | *Helicops trivittatus* |  |
| Dipsadidae | Xenodontinae | Hydropsini | *Helicops* | *Helicops yacu* |  |
| Dipsadidae | Carphophiinae | - | *Heterodon* | *Heterodon kennerlyi* |  |
| Dipsadidae | Carphophiinae | - | *Heterodon* | *Heterodon nasicus* |  |
| Dipsadidae | Carphophiinae | - | *Heterodon* | *Heterodon platirhinos* |  |
| Dipsadidae | Carphophiinae | - | *Heterodon* | *Heterodon simus* |  |
| Dipsadidae | Xenodontinae | Hydronastini | *Hydrodynastes* | *Hydrodynastes bicinctus* |  |
| Dipsadidae | Xenodontinae | Hydronastini | *Hydrodynastes* | *Hydrodynastes gigas* |  |
| Dipsadidae | Dipsadinae | Tribenov.6 | *Hydromorphus* | *Hydromorphus concolor* |  |
| Dipsadidae | Dipsadinae | Tribenov.6 | *Hydromorphus* | *Hydromorphus dunni* |  |
| Dipsadidae | Xenodontinae | Hydropsini | *Hydrops* | *Hydrops caesurus* |  |
| Dipsadidae | Xenodontinae | Hydropsini | *Hydrops* | *Hydrops martii* |  |
| Dipsadidae | Xenodontinae | Hydropsini | *Hydrops* | *Hydrops triangularis* |  |
| Dipsadidae | Dipsadinae | exNothopsini | *Hypsiglena* | *Hypsiglena affinis* |  |
| Dipsadidae | Dipsadinae | exNothopsini | *Hypsiglena* | *Hypsiglena catalinae* |  |
| Dipsadidae | Dipsadinae | exNothopsini | *Hypsiglena* | *Hypsiglena chlorophaea* |  |
| Dipsadidae | Dipsadinae | exNothopsini | *Hypsiglena* | *Hypsiglena jani* |  |
| Dipsadidae | Dipsadinae | exNothopsini | *Hypsiglena* | *Hypsiglena ochrorhynchus* |  |
| Dipsadidae | Dipsadinae | exNothopsini | *Hypsiglena* | *Hypsiglena slevini* |  |
| Dipsadidae | Dipsadinae | exNothopsini | *Hypsiglena* | *Hypsiglena tanzeri* |  |
| Dipsadidae | Dipsadinae | exNothopsini | *Hypsiglena* | *Hypsiglena torquata* |  |
| Dipsadidae | Dipsadinae | exNothopsini | *Hypsiglena* | *Hypsiglena unaocularus* |  |
| Dipsadidae | Xenodontinae | Alsophini | *Hypsirhynchus* | *Hypsirhynchus ater* |  |
| Dipsadidae | Xenodontinae | Alsophini | *Hypsirhynchus* | *Hypsirhynchus callilaemus* |  |
| Dipsadidae | Xenodontinae | Alsophini | *Hypsirhynchus* | *Hypsirhynchus ferox* |  |
| Dipsadidae | Xenodontinae | Alsophini | *Hypsirhynchus* | *Hypsirhynchus funereus* |  |
| Dipsadidae | Xenodontinae | Alsophini | *Hypsirhynchus* | *Hypsirhynchus melanichnus* |  |
| Dipsadidae | Xenodontinae | Alsophini | *Hypsirhynchus* | *Hypsirhynchus parvifrons* |  |
| Dipsadidae | Xenodontinae | Alsophini | *Hypsirhynchus* | *Hypsirhynchus polylepis* |  |
| Dipsadidae | Xenodontinae | Alsophini | *Hypsirhynchus* | *Hypsirhynchus scalaris* |  |
| Dipsadidae | Xenodontinae | Alsophini | *Ialtris* | *Ialtris agyrtes* |  |
| Dipsadidae | Xenodontinae | Alsophini | *Ialtris* | *Ialtris dorsalis* |  |
| Dipsadidae | Xenodontinae | Alsophini | *Ialtris* | *Ialtris haetianus* |  |
| Dipsadidae | Xenodontinae | Alsophini | *Ialtris* | *Ialtris parishi* |  |
| Dipsadidae | Dipsadinae | Imadontini | *Imantodes* | *Imantodes cenchoa* |  |
| Dipsadidae | Dipsadinae | Imadontini | *Imantodes* | *Imantodes chocoensis* |  |
| Dipsadidae | Dipsadinae | Imadontini | *Imantodes* | *Imantodes gemmistratus* |  |
| Dipsadidae | Dipsadinae | Imadontini | *Imantodes* | *Imantodes guane* |  |
| Dipsadidae | Dipsadinae | Imadontini | *Imantodes* | *Imantodes inornatus* |  |
| Dipsadidae | Dipsadinae | Imadontini | *Imantodes* | *Imantodes lentiferus* |  |
| Dipsadidae | Dipsadinae | Imadontini | *Imantodes* | *Imantodes phantasma* |  |
| Dipsadidae | Dipsadinae | Imadontini | *Imantodes* | *Imantodes tenuissimus* |  |
| Dipsadidae | Xenodontinae | Philodryadini | *Incaspis* | *Incaspis amaru* | Generic classification following Melo-Sampaio et al. (2020) |
| Dipsadidae | Xenodontinae | Philodryadini | *Incaspis* | *Incaspis simonsii* | Generic classification following Melo-Sampaio et al. (2020) |
| Dipsadidae | Xenodontinae | Philodryadini | *Incaspis* | *Incaspis tachymenoides* | Generic classification following Melo-Sampaio et al. (2020) |
| Dipsadidae | Dipsadinae | Imadontini | *Leptodeira* | *Leptodeira annulata* |  |
| Dipsadidae | Dipsadinae | Imadontini | *Leptodeira* | *Leptodeira approximans* |  |
| Dipsadidae | Dipsadinae | Imadontini | *Leptodeira* | *Leptodeira ashmeadii* |  |
| Dipsadidae | Dipsadinae | Imadontini | *Leptodeira* | *Leptodeira bakeri* |  |
| Dipsadidae | Dipsadinae | Imadontini | *Leptodeira* | *Leptodeira frenata* |  |
| Dipsadidae | Dipsadinae | Imadontini | *Leptodeira* | *Leptodeira larcorum* |  |
| Dipsadidae | Dipsadinae | Imadontini | *Leptodeira* | *Leptodeira maculata* |  |
| Dipsadidae | Dipsadinae | Imadontini | *Leptodeira* | *Leptodeira misinawui* |  |
| Dipsadidae | Dipsadinae | Imadontini | *Leptodeira* | *Leptodeira nigrofasciata* |  |
| Dipsadidae | Dipsadinae | Imadontini | *Leptodeira* | *Leptodeira ornata* |  |
| Dipsadidae | Dipsadinae | Imadontini | *Leptodeira* | *Leptodeira polysticta* |  |
| Dipsadidae | Dipsadinae | Imadontini | *Leptodeira* | *Leptodeira pulchriceps* |  |
| Dipsadidae | Dipsadinae | Imadontini | *Leptodeira* | *Leptodeira punctata* |  |
| Dipsadidae | Dipsadinae | Imadontini | *Leptodeira* | *Leptodeira rhombifera* |  |
| Dipsadidae | Dipsadinae | Imadontini | *Leptodeira* | *Leptodeira rubricata* |  |
| Dipsadidae | Dipsadinae | Imadontini | *Leptodeira* | *Leptodeira septentrionalis* |  |
| Dipsadidae | Dipsadinae | Imadontini | *Leptodeira* | *Leptodeira splendida* |  |
| Dipsadidae | Dipsadinae | Imadontini | *Leptodeira* | *Leptodeira tarairiu* |  |
| Dipsadidae | Dipsadinae | Imadontini | *Leptodeira* | *Leptodeira uribei* |  |
| Dipsadidae | Insertae sedis in Dipsadidae 2 | Insertae sedis family2 | *Lioheterophis* | *Lioheterophis iheringi* |  |
| Dipsadidae | Xenodontinae | Xenodontini | *Lygophis* | *Lygophis anomalus* |  |
| Dipsadidae | Xenodontinae | Xenodontini | *Lygophis* | *Lygophis dilepis* |  |
| Dipsadidae | Xenodontinae | Xenodontini | *Lygophis* | *Lygophis elegantissimus* |  |
| Dipsadidae | Xenodontinae | Xenodontini | *Lygophis* | *Lygophis flavifrenatus* |  |
| Dipsadidae | Xenodontinae | Xenodontini | *Lygophis* | *Lygophis lineatus* |  |
| Dipsadidae | Xenodontinae | Xenodontini | *Lygophis* | *Lygophis meridionalis* |  |
| Dipsadidae | Xenodontinae | Xenodontini | *Lygophis* | *Lygophis paucidens* |  |
| Dipsadidae | Xenodontinae | Xenodontini | *Lygophis* | *Lygophis vanzolinii* |  |
| Dipsadidae | Xenodontinae | Alsophini | *Magliophis* | *Magliophis exiguus* |  |
| Dipsadidae | Xenodontinae | Alsophini | *Magliophis* | *Magliophis stahli* |  |
| **Dipsadidae** | **Dipsadinae** | **Tribenov.3** | ***Manolepis*** | ***Manolepis putnami*** | For the TACT analysis we performed, we considered the single species of Manolepis (M. putmani) as part of the Dipsadinae, as was recovered in the phylogeny of X. However, no other previous work has found evidence supporting this relationship. Instead, previous studies recognize this species as part of the Xenodontinae. |
| Dipsadidae | Xenodontinae | Tachymenini | *Mesotes* | *Mesotes rutilus* | Generic classification following Trevine et al. (2022) |
| Dipsadidae | Xenodontinae | Tachymenini | *Mesotes* | *Mesotes strigatus* | Generic classification following Trevine et al. (2022) |
| Dipsadidae | Xenodontinae | Pseudoboini | *Mussurana* | *Mussurana bicolor* |  |
| Dipsadidae | Xenodontinae | Pseudoboini | *Mussurana* | *Mussurana montana* |  |
| Dipsadidae | Xenodontinae | Pseudoboini | *Mussurana* | *Mussurana quimi* |  |
| Dipsadidae | Dipsadinae | Tribenov.4 | *Ninia* | *Ninia atrata* | Tribal classification following Sheehy (2012) |
| Dipsadidae | Dipsadinae | Tribenov.4 | *Ninia* | *Ninia celata* | Tribal classification following Sheehy (2012) |
| Dipsadidae | Dipsadinae | Tribenov.4 | *Ninia* | *Ninia diademata* | Tribal classification following Sheehy (2012) |
| Dipsadidae | Dipsadinae | Tribenov.4 | *Ninia* | *Ninia espinali* | Tribal classification following Sheehy (2012) |
| Dipsadidae | Dipsadinae | Tribenov.4 | *Ninia* | *Ninia franciscoi* | Tribal classification following Sheehy (2012) |
| Dipsadidae | Dipsadinae | Tribenov.4 | *Ninia* | *Ninia hudsoni* | Tribal classification following Sheehy (2012) |
| Dipsadidae | Dipsadinae | Tribenov.4 | *Ninia* | *Ninia maculata* | Tribal classification following Sheehy (2012) |
| Dipsadidae | Dipsadinae | Tribenov.4 | *Ninia* | *Ninia pavimentata* | Tribal classification following Sheehy (2012) |
| Dipsadidae | Dipsadinae | Tribenov.4 | *Ninia* | *Ninia psephota* | Tribal classification following Sheehy (2012) |
| **Dipsadidae** | **Xenodontinae** | **Pseudoboini** | ***"Ninia"*** | ***"Ninia" sebae*** | For the TACT analysis we performed, we considered Ninia sebae as part of the Xenodontinae, as was recovered in the phylogeny of X. However, no other previous work has found evidence supporting this relationship. Instead, previous studies recognize this species as part of the Dipsadinae. |
| Dipsadidae | Dipsadinae | Tribenov.4 | *Ninia* | *Ninia teresitae* | Tribal classification following Sheehy (2012) |
| Dipsadidae | Dipsadinae | Nothopsini | *Nothopsis* | *Nothopsis rugosus* |  |
| Dipsadidae | Dipsadinae | Insertae sedis in Dipsadinae3 | *Omoadiphas* | *Omoadiphas aurula* |  |
| Dipsadidae | Dipsadinae | Insertae sedis in Dipsadinae3 | *Omoadiphas* | *Omoadiphas cannula* |  |
| Dipsadidae | Dipsadinae | Insertae sedis in Dipsadinae3 | *Omoadiphas* | *Omoadiphas texiguatensis* |  |
| Dipsadidae | Xenodontinae | Pseudoboini | *Oxyrhopus* | *Oxyrhopus clathratus* |  |
| Dipsadidae | Xenodontinae | Pseudoboini | *Oxyrhopus* | *Oxyrhopus doliatus* |  |
| Dipsadidae | Xenodontinae | Pseudoboini | *Oxyrhopus* | *Oxyrhopus emberti* |  |
| Dipsadidae | Xenodontinae | Pseudoboini | *Oxyrhopus* | *Oxyrhopus erdisii* |  |
| Dipsadidae | Xenodontinae | Pseudoboini | *Oxyrhopus* | *Oxyrhopus fitzingeri* |  |
| Dipsadidae | Xenodontinae | Pseudoboini | *Oxyrhopus* | *Oxyrhopus formosus* |  |
| Dipsadidae | Xenodontinae | Pseudoboini | *Oxyrhopus* | *Oxyrhopus guibei* |  |
| Dipsadidae | Xenodontinae | Pseudoboini | *Oxyrhopus* | *Oxyrhopus leucomelas* |  |
| Dipsadidae | Xenodontinae | Pseudoboini | *Oxyrhopus* | *Oxyrhopus marcapatae* |  |
| Dipsadidae | Xenodontinae | Pseudoboini | *Oxyrhopus* | *Oxyrhopus melanogenys* |  |
| Dipsadidae | Xenodontinae | Pseudoboini | *Oxyrhopus* | *Oxyrhopus occipitalis* |  |
| Dipsadidae | Xenodontinae | Pseudoboini | *Oxyrhopus* | *Oxyrhopus petolarius* |  |
| Dipsadidae | Xenodontinae | Pseudoboini | *Oxyrhopus* | *Oxyrhopus rhombifer* |  |
| Dipsadidae | Xenodontinae | Pseudoboini | *Oxyrhopus* | *Oxyrhopus trigeminus* |  |
| Dipsadidae | Xenodontinae | Pseudoboini | *Oxyrhopus* | *Oxyrhopus vanidicus* |  |
| Dipsadidae | Xenodontinae | Pseudoboini | *Paraphimophis* | *Paraphimophis rusticus* |  |
| Dipsadidae | Xenodontinae | Elapomorphini | *Phalotris* | *Phalotris bilineatus* |  |
| Dipsadidae | Xenodontinae | Elapomorphini | *Phalotris* | *Phalotris concolor* |  |
| Dipsadidae | Xenodontinae | Elapomorphini | *Phalotris* | *Phalotris cuyanus* |  |
| Dipsadidae | Xenodontinae | Elapomorphini | *Phalotris* | *Phalotris illustrator* |  |
| Dipsadidae | Xenodontinae | Elapomorphini | *Phalotris* | *Phalotris labiomaculatus* |  |
| Dipsadidae | Xenodontinae | Elapomorphini | *Phalotris* | *Phalotris lativittatus* |  |
| Dipsadidae | Xenodontinae | Elapomorphini | *Phalotris* | *Phalotris lemniscatus* |  |
| Dipsadidae | Xenodontinae | Elapomorphini | *Phalotris* | *Phalotris matogrossensis* |  |
| Dipsadidae | Xenodontinae | Elapomorphini | *Phalotris* | *Phalotris mertensi* |  |
| Dipsadidae | Xenodontinae | Elapomorphini | *Phalotris* | *Phalotris multipunctatus* |  |
| Dipsadidae | Xenodontinae | Elapomorphini | *Phalotris* | *Phalotris nasutus* |  |
| Dipsadidae | Xenodontinae | Elapomorphini | *Phalotris* | *Phalotris nigrilatus* |  |
| Dipsadidae | Xenodontinae | Elapomorphini | *Phalotris* | *Phalotris normanscotti* |  |
| Dipsadidae | Xenodontinae | Elapomorphini | *Phalotris* | *Phalotris reticulatus* |  |
| Dipsadidae | Xenodontinae | Elapomorphini | *Phalotris* | *Phalotris sansebastiani* |  |
| Dipsadidae | Xenodontinae | Elapomorphini | *Phalotris* | *Phalotris shawnella* |  |
| Dipsadidae | Xenodontinae | Elapomorphini | *Phalotris* | *Phalotris spegazzinii* |  |
| Dipsadidae | Xenodontinae | Elapomorphini | *Phalotris* | *Phalotris suspectus* |  |
| Dipsadidae | Xenodontinae | Elapomorphini | *Phalotris* | *Phalotris tricolor* |  |
| Dipsadidae | Xenodontinae | Philodryadini | *Philodryas* | *Philodryas aestiva* |  |
| Dipsadidae | Xenodontinae | Philodryadini | *Philodryas* | *Philodryas agassizii* |  |
| Dipsadidae | Xenodontinae | Philodryadini | *Philodryas* | *Philodryas arnaldoi* |  |
| Dipsadidae | Xenodontinae | Philodryadini | *Philodryas* | *Philodryas baroni* |  |
| Dipsadidae | Xenodontinae | Philodryadini | *Philodryas* | *Philodryas boliviana* |  |
| Dipsadidae | Xenodontinae | Philodryadini | *Philodryas* | *Philodryas chamissonis* |  |
| Dipsadidae | Xenodontinae | Philodryadini | *Philodryas* | *Philodryas cordata* |  |
| Dipsadidae | Xenodontinae | Philodryadini | *Philodryas* | *Philodryas erlandi* |  |
| Dipsadidae | Xenodontinae | Philodryadini | *Philodryas* | *Philodryas livida* |  |
| Dipsadidae | Xenodontinae | Philodryadini | *Philodryas* | *Philodryas mattogrossensis* |  |
| Dipsadidae | Xenodontinae | Philodryadini | *Philodryas* | *Philodryas nattereri* |  |
| Dipsadidae | Xenodontinae | Philodryadini | *Philodryas* | *Philodryas olfersii* |  |
| Dipsadidae | Xenodontinae | Philodryadini | *Philodryas* | *Philodryas patagoniensis* |  |
| Dipsadidae | Xenodontinae | Philodryadini | *Philodryas* | *Philodryas psammophidea* |  |
| Dipsadidae | Xenodontinae | Philodryadini | *Philodryas* | *Philodryas trilineata* |  |
| Dipsadidae | Xenodontinae | Philodryadini | *Philodryas* | *Philodryas varia* |  |
| Dipsadidae | Xenodontinae | Pseudoboini | *Phimophis* | *Phimophis guerini* |  |
| Dipsadidae | Xenodontinae | Pseudoboini | *Phimophis* | *Phimophis guianensis* |  |
| Dipsadidae | Xenodontinae | Pseudoboini | *Phimophis* | *Phimophis vittatus* |  |
| Dipsadidae | Dipsadinae | Dipsadini | *Plesiodipsas* | *Plesiodipsas perijanensis* |  |
| Dipsadidae | Dipsadinae | Tribenov.3 | *Pliocercus* | *Pliocercus elapoides* | Tribal classification following Sheehy (2012) |
| Dipsadidae | Dipsadinae | Tribenov.3 | *Pliocercus* | *Pliocercus euryzonus* | Tribal classification following Sheehy (2012) |
| Dipsadidae | Xenodontinae | Insertae sedis in Xenodontinae2 | *Pseudalsophis* | *Pseudalsophis biserialis* |  |
| Dipsadidae | Xenodontinae | Insertae sedis in Xenodontinae2 | *Pseudalsophis* | *Pseudalsophis darwini* |  |
| Dipsadidae | Xenodontinae | Insertae sedis in Xenodontinae2 | *Pseudalsophis* | *Pseudalsophis dorsalis* |  |
| Dipsadidae | Xenodontinae | Insertae sedis in Xenodontinae2 | *Pseudalsophis* | *Pseudalsophis elegans* |  |
| Dipsadidae | Xenodontinae | Insertae sedis in Xenodontinae2 | *Pseudalsophis* | *Pseudalsophis hephaestus* |  |
| Dipsadidae | Xenodontinae | Insertae sedis in Xenodontinae2 | *Pseudalsophis* | *Pseudalsophis hoodensis* |  |
| Dipsadidae | Xenodontinae | Insertae sedis in Xenodontinae2 | *Pseudalsophis* | *Pseudalsophis occidentalis* |  |
| Dipsadidae | Xenodontinae | Insertae sedis in Xenodontinae2 | *Pseudalsophis* | *Pseudalsophis slevini* |  |
| Dipsadidae | Xenodontinae | Insertae sedis in Xenodontinae2 | *Pseudalsophis* | *Pseudalsophis steindachneri* |  |
| Dipsadidae | Xenodontinae | Insertae sedis in Xenodontinae2 | *Pseudalsophis* | *Pseudalsophis thomasi* |  |
| Dipsadidae | Xenodontinae | Pseudoboini | *Pseudoboa* | *Pseudoboa coronata* |  |
| Dipsadidae | Xenodontinae | Pseudoboini | *Pseudoboa* | *Pseudoboa haasi* |  |
| Dipsadidae | Xenodontinae | Pseudoboini | *Pseudoboa* | *Pseudoboa martinsi* |  |
| Dipsadidae | Xenodontinae | Pseudoboini | *Pseudoboa* | *Pseudoboa neuwiedii* |  |
| Dipsadidae | Xenodontinae | Pseudoboini | *Pseudoboa* | *Pseudoboa nigra* |  |
| Dipsadidae | Xenodontinae | Pseudoboini | *Pseudoboa* | *Pseudoboa serrana* |  |
| Dipsadidae | Xenodontinae | Hydropsini | *Pseudoeryx* | *Pseudoeryx plicatilis* |  |
| Dipsadidae | Xenodontinae | Hydropsini | *Pseudoeryx* | *Pseudoeryx relictualis* |  |
| Dipsadidae | Dipsadinae | exNothopsini | *Pseudoleptodeira* | *Pseudoleptodeira latifasciata* |  |
| Dipsadidae | Xenodontinae | Psomophini | *Psomophis* | *Psomophis genimaculatus* |  |
| Dipsadidae | Xenodontinae | Psomophini | *Psomophis* | *Psomophis joberti* |  |
| Dipsadidae | Xenodontinae | Psomophini | *Psomophis* | *Psomophis obtusus* |  |
| Dipsadidae | Xenodontinae | Tachymenini | *Ptychophis* | *Ptychophis flavovirgatus* |  |
| Dipsadidae | Xenodontinae | Pseudoboini | *Rhachidelus* | *Rhachidelus brazili* |  |
| Dipsadidae | Dipsadinae | Tribenov.3 | *Rhadinaea* | *Rhadinaea bogertorum* | Tribal classification following Sheehy (2012) |
| Dipsadidae | Dipsadinae | Tribenov.3 | *Rhadinaea* | *Rhadinaea calligaster* | Tribal classification following Sheehy (2012) |
| Dipsadidae | Dipsadinae | Tribenov.3 | *Rhadinaea* | *Rhadinaea cuneata* | Tribal classification following Sheehy (2012) |
| Dipsadidae | Dipsadinae | Tribenov.3 | *Rhadinaea* | *Rhadinaea decorata* | Tribal classification following Sheehy (2012) |
| Dipsadidae | Dipsadinae | Tribenov.3 | *Rhadinaea* | *Rhadinaea flavilata* | Tribal classification following Sheehy (2012) |
| Dipsadidae | Dipsadinae | Tribenov.3 | *Rhadinaea* | *Rhadinaea forbesi* | Tribal classification following Sheehy (2012) |
| Dipsadidae | Dipsadinae | Tribenov.3 | *Rhadinaea* | *Rhadinaea fulvivittis* | Tribal classification following Sheehy (2012) |
| Dipsadidae | Dipsadinae | Tribenov.3 | *Rhadinaea* | *Rhadinaea gaigeae* | Tribal classification following Sheehy (2012) |
| Dipsadidae | Dipsadinae | Tribenov.3 | *Rhadinaea* | *Rhadinaea hesperia* | Tribal classification following Sheehy (2012) |
| Dipsadidae | Dipsadinae | Tribenov.3 | *Rhadinaea* | *Rhadinaea laureata* | Tribal classification following Sheehy (2012) |
| Dipsadidae | Dipsadinae | Tribenov.3 | *Rhadinaea* | *Rhadinaea macdougalli* | Tribal classification following Sheehy (2012) |
| Dipsadidae | Dipsadinae | Tribenov.3 | *Rhadinaea* | *Rhadinaea marcellae* | Tribal classification following Sheehy (2012) |
| Dipsadidae | Dipsadinae | Tribenov.3 | *Rhadinaea* | *Rhadinaea montana* | Tribal classification following Sheehy (2012) |
| Dipsadidae | Dipsadinae | Tribenov.3 | *Rhadinaea* | *Rhadinaea myersi* | Tribal classification following Sheehy (2012) |
| Dipsadidae | Dipsadinae | Tribenov.3 | *Rhadinaea* | *Rhadinaea nuchalis* | Tribal classification following Sheehy (2012) |
| Dipsadidae | Dipsadinae | Tribenov.3 | *Rhadinaea* | *Rhadinaea omiltemana* | Tribal classification following Sheehy (2012) |
| Dipsadidae | Dipsadinae | Tribenov.3 | *Rhadinaea* | *Rhadinaea pulveriventris* | Tribal classification following Sheehy (2012) |
| Dipsadidae | Dipsadinae | Tribenov.3 | *Rhadinaea* | *Rhadinaea quinquelineata* | Tribal classification following Sheehy (2012) |
| Dipsadidae | Dipsadinae | Tribenov.3 | *Rhadinaea* | *Rhadinaea sargenti* | Tribal classification following Sheehy (2012) |
| Dipsadidae | Dipsadinae | Tribenov.3 | *Rhadinaea* | *Rhadinaea taeniata* | Tribal classification following Sheehy (2012) |
| Dipsadidae | Dipsadinae | Tribenov.3 | *Rhadinaea* | *Rhadinaea vermiculaticeps* | Tribal classification following Sheehy (2012) |
| Dipsadidae | Dipsadinae | Tribenov.3 | *Rhadinella* | *Rhadinella anachoreta* | Tribal classification following Sheehy (2012) |
| Dipsadidae | Dipsadinae | Tribenov.3 | *Rhadinella* | *Rhadinella donaji* | Tribal classification following Sheehy (2012) |
| Dipsadidae | Dipsadinae | Tribenov.3 | *Rhadinella* | *Rhadinella dysmica* | Tribal classification following Sheehy (2012) |
| Dipsadidae | Dipsadinae | Tribenov.3 | *Rhadinella* | *Rhadinella godmani* | Tribal classification following Sheehy (2012) |
| Dipsadidae | Dipsadinae | Tribenov.3 | *Rhadinella* | *Rhadinella hannsteini* | Tribal classification following Sheehy (2012) |
| Dipsadidae | Dipsadinae | Tribenov.3 | *Rhadinella* | *Rhadinella hempsteadae* | Tribal classification following Sheehy (2012) |
| Dipsadidae | Dipsadinae | Tribenov.3 | *Rhadinella* | *Rhadinella kanalchutchan* | Tribal classification following Sheehy (2012) |
| Dipsadidae | Dipsadinae | Tribenov.3 | *Rhadinella* | *Rhadinella kinkelini* | Tribal classification following Sheehy (2012) |
| Dipsadidae | Dipsadinae | Tribenov.3 | *Rhadinella* | *Rhadinella lachrymans* | Tribal classification following Sheehy (2012) |
| Dipsadidae | Dipsadinae | Tribenov.3 | *Rhadinella* | *Rhadinella lisyae* | Tribal classification following Sheehy (2012) |
| Dipsadidae | Dipsadinae | Tribenov.3 | *Rhadinella* | *Rhadinella montecristi* | Tribal classification following Sheehy (2012) |
| Dipsadidae | Dipsadinae | Tribenov.3 | *Rhadinella* | *Rhadinella pegosalyta* | Tribal classification following Sheehy (2012) |
| Dipsadidae | Dipsadinae | Tribenov.3 | *Rhadinella* | *Rhadinella pilonaorum* | Tribal classification following Sheehy (2012) |
| Dipsadidae | Dipsadinae | Tribenov.3 | *Rhadinella* | *Rhadinella posadasi* | Tribal classification following Sheehy (2012) |
| Dipsadidae | Dipsadinae | Tribenov.3 | *Rhadinella* | *Rhadinella rogerromani* | Tribal classification following Sheehy (2012) |
| Dipsadidae | Dipsadinae | Tribenov.3 | *Rhadinella* | *Rhadinella schistosa* | Tribal classification following Sheehy (2012) |
| Dipsadidae | Dipsadinae | Tribenov.3 | *Rhadinella* | *Rhadinella serperaster* | Tribal classification following Sheehy (2012) |
| Dipsadidae | Dipsadinae | Tribenov.3 | *Rhadinella* | *Rhadinella stadelmani* | Tribal classification following Sheehy (2012) |
| Dipsadidae | Dipsadinae | Tribenov.3 | *Rhadinella* | *Rhadinella tolpanorum* | Tribal classification following Sheehy (2012) |
| Dipsadidae | Dipsadinae | Tribenov.3 | *Rhadinella* | *Rhadinella xerophila* | Tribal classification following Sheehy (2012) |
| Dipsadidae | Dipsadinae | Tribenov.7 | *Rhadinophanes* | *Rhadinophanes monticola* | Tribal classification following Sheehy (2012) |
| Dipsadidae | Xenodontinae | Pseudoboini | *Rodriguesophis* | *Rodriguesophis chui* |  |
| Dipsadidae | Xenodontinae | Pseudoboini | *Rodriguesophis* | *Rodriguesophis iglesiasi* |  |
| Dipsadidae | Xenodontinae | Pseudoboini | *Rodriguesophis* | *Rodriguesophis scriptorcibatus* |  |
| Dipsadidae | Xenodontinae | Saphenophini | *Saphenophis* | *Saphenophis antioquiensis* |  |
| Dipsadidae | Xenodontinae | Saphenophini | *Saphenophis* | *Saphenophis atahuallpae* |  |
| Dipsadidae | Xenodontinae | Saphenophini | *Saphenophis* | *Saphenophis boursieri* |  |
| Dipsadidae | Xenodontinae | Saphenophini | *Saphenophis* | *Saphenophis sneiderni* |  |
| Dipsadidae | Xenodontinae | Saphenophini | *Saphenophis* | *Saphenophis tristriatus* |  |
| Dipsadidae | Dipsadinae | Dipsadini | *Sibon* | *Sibon annulatus* |  |
| Dipsadidae | Dipsadinae | Dipsadini | *Sibon* | *Sibon anthracops* |  |
| Dipsadidae | Dipsadinae | Dipsadini | *Sibon* | *Sibon argus* |  |
| Dipsadidae | Dipsadinae | Dipsadini | *Sibon* | *Sibon ayerbeorum* |  |
| Dipsadidae | Dipsadinae | Dipsadini | *Sibon* | *Sibon bevridgelyi* |  |
| Dipsadidae | Dipsadinae | Dipsadini | *Sibon* | *Sibon carri* |  |
| Dipsadidae | Dipsadinae | Dipsadini | *Sibon* | *Sibon dimidiatus* |  |
| Dipsadidae | Dipsadinae | Dipsadini | *Sibon* | *Sibon dunni* |  |
| Dipsadidae | Dipsadinae | Dipsadini | *Sibon* | *Sibon lamari* |  |
| Dipsadidae | Dipsadinae | Dipsadini | *Sibon* | *Sibon linearis* |  |
| Dipsadidae | Dipsadinae | Dipsadini | *Sibon* | *Sibon longifrenis* |  |
| Dipsadidae | Dipsadinae | Dipsadini | *Sibon* | *Sibon manzanaresi* |  |
| Dipsadidae | Dipsadinae | Dipsadini | *Sibon* | *Sibon merendonensis* |  |
| Dipsadidae | Dipsadinae | Dipsadini | *Sibon* | *Sibon miskitus* |  |
| Dipsadidae | Dipsadinae | Dipsadini | *Sibon* | *Sibon nebulatus* |  |
| Dipsadidae | Dipsadinae | Dipsadini | *Sibon* | *Sibon noalamina* |  |
| Dipsadidae | Dipsadinae | Dipsadini | *Sibon* | *Sibon perissostichon* |  |
| Dipsadidae | Xenodontinae | Pseudoboini | *Siphlophis* | *Siphlophis ayauma* |  |
| Dipsadidae | Xenodontinae | Pseudoboini | *Siphlophis* | *Siphlophis cervinus* |  |
| **Dipsadidae** | **Xenodontinae** | **Tachymenini** | ***"Siphlophis"*** | ***"Siphlophis" compressus*** |  |
| Dipsadidae | Xenodontinae | Pseudoboini | *Siphlophis* | *Siphlophis leucocephalus* |  |
| Dipsadidae | Xenodontinae | Pseudoboini | *Siphlophis* | *Siphlophis longicaudatus* |  |
| Dipsadidae | Xenodontinae | Pseudoboini | *Siphlophis* | *Siphlophis pulcher* |  |
| Dipsadidae | Xenodontinae | Pseudoboini | *Siphlophis* | *Siphlophis worontzowi* |  |
| Dipsadidae | Xenodontinae | Echinantherini | *Sordellina* | *Sordellina punctata* |  |
| Dipsadidae | - | - | *Stichophanes* | *Stichophanes ningshaanensis* |  |
| Dipsadidae | Dipsadinae | Diaphorolepini | *Synophis* | *Synophis bicolor* |  |
| Dipsadidae | Dipsadinae | Diaphorolepini | *Synophis* | *Synophis bogerti* |  |
| Dipsadidae | Dipsadinae | Diaphorolepini | *Synophis* | *Synophis calamitus* |  |
| Dipsadidae | Dipsadinae | Diaphorolepini | *Synophis* | *Synophis insulomontanus* |  |
| Dipsadidae | Dipsadinae | Diaphorolepini | *Synophis* | *Synophis lasallei* |  |
| Dipsadidae | Dipsadinae | Diaphorolepini | *Synophis* | *Synophis niceforomariae* |  |
| Dipsadidae | Dipsadinae | Diaphorolepini | *Synophis* | *Synophis plectovertebralis* |  |
| Dipsadidae | Dipsadinae | Diaphorolepini | *Synophis* | *Synophis zaheri* |  |
| Dipsadidae | Dipsadinae | Diaphorolepini | *Synophis* | *Synophis zamora* |  |
| Dipsadidae | Xenodontinae | Tachymenini | *Tachymenis* | *Tachymenis ocellata* |  |
| Dipsadidae | Xenodontinae | Tachymenini | *Tachymenis* | *Tachymenis peruviana* |  |
| Dipsadidae | Xenodontinae | Tachymenini | *Tachymenis* | *Tachymenis trigonatus* |  |
| Dipsadidae | Xenodontinae | Tachymenini | *Tachymenoides* | *Tachymenoides affinis* | Generic classification following Trevine et al. (2022) |
| Dipsadidae | Xenodontinae | Echinantherini | *Taeniophallus* | *Taeniophallus brevirostris* |  |
| Dipsadidae | Xenodontinae | Echinantherini | *Taeniophallus* | *Taeniophallus nebularis* |  |
| Dipsadidae | Xenodontinae | Echinantherini | *Taeniophallus* | *Taeniophallus nicagus* |  |
| Dipsadidae | Dipsadinae | Tribenov.7 | *Tantalophis* | *Tantalophis discolor* | Tribal classification following Sheehy (2012) |
| Dipsadidae | Xenodontinae | Tachymenini | *Thamnodynastes* | *Thamnodynastes longicauda* |  |
| Dipsadidae | Xenodontinae | Tachymenini | *Thamnodynastes* | *Thamnodynastes pallidus* |  |
| Dipsadidae | Xenodontinae | Tachymenini | *Thamnodynastes* | *Thamnodynastes sertanejo* |  |
| Dipsadidae | Xenodontinae | Tachymenini | *Thamnodynastes* | *Thamnodynastes silvai* |  |
| Dipsadidae | - | - | *Thermophis* | *Thermophis baileyi* |  |
| Dipsadidae | - | - | *Thermophis* | *Thermophis shangrila* |  |
| Dipsadidae | - | - | *Thermophis* | *Thermophis zhaoermii* |  |
| Dipsadidae | Xenodontinae | Tachymenini | *Tomodon* | *Tomodon dorsatus* |  |
| Dipsadidae | Dipsadinae | Tribenov.6 | *Tretanorhinus* | *Tretanorhinus mocquardi* | Tribal classification following Sheehy (2012) |
| Dipsadidae | Dipsadinae | Tribenov.6 | *Tretanorhinus* | *Tretanorhinus nigroluteus* | Tribal classification following Sheehy (2012) |
| Dipsadidae | Dipsadinae | Tribenov.6 | *Tretanorhinus* | *Tretanorhinus taeniatus* | Tribal classification following Sheehy (2012) |
| Dipsadidae | Dipsadinae | Tribenov.6 | *Tretanorhinus* | *Tretanorhinus variabilis* | Tribal classification following Sheehy (2012) |
| Dipsadidae | Dipsadinae | Tribenov.3 | *Trimetopon* | *Trimetopon barbouri* | Tribal classification following Sheehy (2012) |
| Dipsadidae | Dipsadinae | Tribenov.3 | *Trimetopon* | *Trimetopon gracile* | Tribal classification following Sheehy (2012) |
| Dipsadidae | Dipsadinae | Tribenov.3 | *Trimetopon* | *Trimetopon pliolepis* | Tribal classification following Sheehy (2012) |
| Dipsadidae | Dipsadinae | Tribenov.3 | *Trimetopon* | *Trimetopon simile* | Tribal classification following Sheehy (2012) |
| Dipsadidae | Dipsadinae | Tribenov.3 | *Trimetopon* | *Trimetopon slevini* | Tribal classification following Sheehy (2012) |
| Dipsadidae | Dipsadinae | Tribenov.3 | *Trimetopon* | *Trimetopon viquezi* | Tribal classification following Sheehy (2012) |
| Dipsadidae | Dipsadinae | Dipsadini | *Tropidodipsas* | *Tropidodipsas fasciata* |  |
| Dipsadidae | Dipsadinae | Dipsadini | *Tropidodipsas* | *Tropidodipsas fischeri* |  |
| Dipsadidae | Dipsadinae | Dipsadini | *Tropidodipsas* | *Tropidodipsas guerreroensis* |  |
| Dipsadidae | Dipsadinae | Dipsadini | *Tropidodipsas* | *Tropidodipsas papavericola* |  |
| Dipsadidae | Dipsadinae | Dipsadini | *Tropidodipsas* | *Tropidodipsas philippii* |  |
| Dipsadidae | Dipsadinae | Dipsadini | *Tropidodipsas* | *Tropidodipsas repleta* |  |
| Dipsadidae | Dipsadinae | Dipsadini | *Tropidodipsas* | *Tropidodipsas tricolor* |  |
| Dipsadidae | Dipsadinae | Dipsadini | *Tropidodipsas* | *Tropidodipsas zweifeli* |  |
| Dipsadidae | Xenodontinae | Tropidodryadini | *Tropidodryas* | *Tropidodryas serra* |  |
| Dipsadidae | Xenodontinae | Tropidodryadini | *Tropidodryas* | *Tropidodryas striaticeps* |  |
| Dipsadidae | Xenodontinae | Alsophini | *Uromacer* | *Uromacer catesbyi* |  |
| Dipsadidae | Xenodontinae | Alsophini | *Uromacer* | *Uromacer frenatus* |  |
| Dipsadidae | Xenodontinae | Alsophini | *Uromacer* | *Uromacer oxyrhynchus* |  |
| Dipsadidae | Dipsadinae | Tribenov.3 | *Urotheca* | *Urotheca decipiens* | Tribal classification following Sheehy (2012) |
| Dipsadidae | Dipsadinae | Tribenov.3 | *Urotheca* | *Urotheca dumerilli* | Tribal classification following Sheehy (2012) |
| Dipsadidae | Dipsadinae | Tribenov.3 | *Urotheca* | *Urotheca fulviceps* | Tribal classification following Sheehy (2012) |
| Dipsadidae | Dipsadinae | Tribenov.3 | *Urotheca* | *Urotheca guentheri* | Tribal classification following Sheehy (2012) |
| Dipsadidae | Dipsadinae | Tribenov.3 | *Urotheca* | *Urotheca lateristriga* | Tribal classification following Sheehy (2012) |
| Dipsadidae | Dipsadinae | Tribenov.3 | *Urotheca* | *Urotheca multilineata* | Tribal classification following Sheehy (2012) |
| Dipsadidae | Dipsadinae | Tribenov.3 | *Urotheca* | *Urotheca myersi* | Tribal classification following Sheehy (2012) |
| Dipsadidae | Dipsadinae | Tribenov.3 | *Urotheca* | *Urotheca pachyura* | Tribal classification following Sheehy (2012) |
| Dipsadidae | Xenodontinae | Xenodontini | *Xenodon* | *Xenodon dorbignyi* |  |
| Dipsadidae | Xenodontinae | Xenodontini | *Xenodon* | *Xenodon guentheri* |  |
| Dipsadidae | Xenodontinae | Xenodontini | *Xenodon* | *Xenodon histricus* |  |
| Dipsadidae | Xenodontinae | Xenodontini | *Xenodon* | *Xenodon matogrossensis* |  |
| Dipsadidae | Xenodontinae | Xenodontini | *Xenodon* | *Xenodon merremii* |  |
| Dipsadidae | Xenodontinae | Xenodontini | *Xenodon* | *Xenodon nattereri* |  |
| Dipsadidae | Xenodontinae | Xenodontini | *Xenodon* | *Xenodon neuwiedii* |  |
| Dipsadidae | Xenodontinae | Xenodontini | *Xenodon* | *Xenodon pulcher* |  |
| Dipsadidae | Xenodontinae | Xenodontini | *Xenodon* | *Xenodon rabdocephalus* |  |
| Dipsadidae | Xenodontinae | Xenodontini | *Xenodon* | *Xenodon semicinctus* |  |
| Dipsadidae | Xenodontinae | Xenodontini | *Xenodon* | *Xenodon severus* |  |
| Dipsadidae | Xenodontinae | Xenodontini | *Xenodon* | *Xenodon werneri* |  |
| Dipsadidae | Xenodontinae | Insertae sedis in Xenodontinae3 | *Xenopholis* | *Xenopholis scalaris* |  |
| Dipsadidae | Xenodontinae | Insertae sedis in Xenodontinae3 | *Xenopholis* | *Xenopholis undulatus* |  |
| Dipsadidae | Xenodontinae | Insertae sedis in Xenodontinae3 | *Xenopholis* | *Xenopholis werdingorum* |  |
| Dipsadidae | Xenodontinae | Philodryadini | *Xenoxybelis* | *Xenoxybelis argenteus* | Generic classification following Melo-Sampaio et al. (2020) |
| Dipsadidae | Xenodontinae | Philodryadini | *Xenoxybelis* | *Xenoxybelis boulengeri* | Generic classification following Melo-Sampaio et al. (2020) |
| Dipsadidae | Xenodontinae | Tachymenini | *Zonateres* | *Zonateres lanei* | Generic classification following Trevine et al. (2022) |
